# Cardiovascular Outcomes of α-Blockers vs 5-α Reductase Inhibitors for Benign Prostatic Hyperplasia

**DOI:** 10.1001/jamanetworkopen.2023.43299

**Published:** 2023-11-14

**Authors:** Jiandong Zhang, Chase D. Latour, Oluwasolape Olawore, Virginia Pate, David F. Friedlander, Til Stürmer, Michele Jonsson Funk, Brian C. Jensen

**Affiliations:** 1 Division of Cardiology, School of Medicine, University of North Carolina at Chapel Hill; 2 Department of Epidemiology, Gillings School of Global Public Health, University of North Carolina at Chapel Hill; 3 Cecil G. Sheps Center for Health Services Research, University of North Carolina at Chapel Hill; 4 Department of Urology, University of North Carolina at Chapel Hill; 5 Lineberger Comprehensive Cancer Center, University of North Carolina at Chapel Hill; 6 Department of Pharmacology, School of Medicine, University of North Carolina at Chapel Hill

## Abstract

**Importance:**

The most prescribed class of medications for benign prostatic hyperplasia (BPH) is α-blockers (ABs). However, the cardiovascular safety profile of these medications among patients with BPH is not well understood.

**Objective:**

To compare the safety of ABs vs 5-α reductase inhibitors (5-ARIs) for risk of adverse cardiovascular outcomes.

**Design, Setting, and Participants:**

This active comparator, new-user cohort study was conducted using insurance claims data from a 20% random sample of Medicare beneficiaries from 2007 to 2019 to evaluate the 1-year risk of adverse cardiovascular outcomes. Males aged 66 to 90 years were indexed into the cohort at new use of an AB or 5-ARI. Twelve months of continuous enrollment and at least 1 diagnosis code for BPH within 12 months prior to initiation were required. Data were analyzed from January 2007 through December 2019.

**Exposures:**

Exposure was defined by a qualifying prescription fill for an AB or 5-ARI after at least 12 months without a prescription for these drug classes.

**Main Outcomes and Measures:**

Follow-up began at a qualified refill for the study drug. Primary study outcomes were hospitalization for heart failure (HF), composite major adverse cardiovascular events (MACE; hospitalization for stroke, myocardial infarction, or death), composite MACE or hospitalization for HF, and death. Inverse probability of treatment and censoring-weighted 1-year risks, risk ratios (RRs), and risk differences (RDs) were estimated for each outcome.

**Results:**

Among 189 868 older adult males, there were 163 829 patients initiating ABs (mean [SD] age, 74.6 [6.2] years; 579 American Indian or Alaska Native [0.4%], 5890 Asian or Pacific Islander [3.6%], 9179 Black [5.6%], 10 610 Hispanic [6.5%], and 133 510 non-Hispanic White [81.5%]) and 26 039 patients initiating 5-ARIs (mean [SD] age, 75.3 [6.4] years; 76 American Indian or Alaska Native [0.3%], 827 Asian or Pacific Islander [3.2%], 1339 Black [5.1%], 1656 Hispanic [6.4%], and 21 605 non-Hispanic White [83.0%]). ABs compared with 5-ARIs were associated with an increased 1-year risk of MACE (8.95% [95% CI, 8.81%-9.09%] vs 8.32% [95% CI, 7.92%-8.72%]; RR = 1.08 [95% CI, 1.02-1.13]; RD per 1000 individuals = 6.26 [95% CI, 2.15-10.37]), composite MACE and HF (RR = 1.07; [95% CI, 1.03-1.12]; RD per 1000 individuals = 7.40 [95% CI, 2.88-11.93 ]), and death (RR = 1.07; [95% CI, 1.01-1.14]; RD per 1000 individuals = 3.85 [95% CI, 0.40-7.29]). There was no difference in risk for HF hospitalization alone.

**Conclusions and Relevance:**

These results suggest that ABs may be associated with an increased risk of adverse cardiovascular outcomes compared with 5-ARIs. If replicated with more detailed confounder data, these results may have important public health implications given these medications’ widespread use.

## Key Points

**Question:**

What is the comparative cardiovascular safety of α -blockers vs 5-α reductase inhibitors for treatment of benign prostatic hyperplasia?

**Findings:**

In this cohort study of 189 868 older adult males, risk of major adverse cardiovascular outcomes was 8.95% among initiators of α-blockers vs 8.32% among initiators of 5-α reductase inhibitors during a 1-year follow-up period. The difference in risk for heart failure hospitalization was not significant.

**Meaning:**

This study found that treatment of benign prostatic hyperplasia with α-blockers was associated with worse cardiovascular outcomes compared with 5-α reductase inhibitors.

## Teaser

This cohort study compares the cardiovascular safety profile of α-blockers vs 5-α reductase inhibitors for patients with benign prostatic hyperplasia.

## Introduction

Cardiovascular diseases and benign prostatic hyperplasia (BPH) are common conditions with shared risk factors among older men.^1^ The most prescribed class of medications for BPH consists of α-1 blockers (ABs), particularly selective antagonists of the α-1A adrenergic receptor (α1-A-AR) subtype.^2^ Interestingly, the α1-A-AR subtype is expressed in prostate and cardiovascular tissues. Preclinical studies found that the α1-A-AR subtype was associated with cardioprotective outcomes.^3,4^ Furthermore, the landmark Antihypertensive and Lipid-Lowering Treatment to Prevent Heart Attack Trial (ALLHAT)^5,6^ was terminated early because doxazosin (a nonselective AB) was associated with an increased risk of adverse cardiac events, most notably HF, angina, and coronary revascularization. However, previous investigations of the cardiac safety of ABs have produced conflicting results.^3,7^

Our 2021 study^8^ found that use of ABs compared with no AB use was associated with an increased risk of mortality among patients undergoing percutaneous coronary intervention for myocardial infarction (MI). This finding highlights the need to investigate AB safety on a broader scale. In this study, we hypothesized that ABs compared with 5-α reductase inhibitors (5-ARIs), the second most prescribed medication class for BPH, would be associated with increased cardiovascular risks among Medicare beneficiaries with BPH.^2^

## Methods

This cohort study was approved by the University of North Carolina at Chapel Hill (UNC-CH) Institutional Review Board, which granted a waiver of informed consent because the risk to patients was no more than minimal. This report follows the Strengthening the Reporting of Observational Studies in Epidemiology (STROBE) reporting guideline.

### Data Source

This study used insurance claims from a 20% random sample of Medicare beneficiaries in the US (2007-2019). This database contains longitudinal claims on inpatient, outpatient, and prescription drug services and demographic data. We obtained data under a data use agreement with the Centers for Medicare & Medicaid Services as maintained by the UNC-CH Cecil G. Sheps Center for Health Services Research.

### Study Cohort

This was an active comparator study comparing new users of ABs vs 5-ARIs for risk of adverse outcomes.^9^ We constructed a cohort of individuals identified as males who were aged 66 to 90 years at new use; had continuous enrollment in fee-for-service Medicare plans A, B, and D for 12 or more months (with a 45-day gap allowed) prior to new use; and had 1 or more diagnosis codes for BPH at 12 months or less prior to new use (eTable 1 in Supplement 1; eTable 24 in Supplement 2). We excluded patients with chemotherapy within 6 months, hospice care within 12 months, or prostatectomy or prostate cancer at any time prior to new use (eTables 25-27 in Supplement 2).

### Exposure Assessment

**Figure 1.**
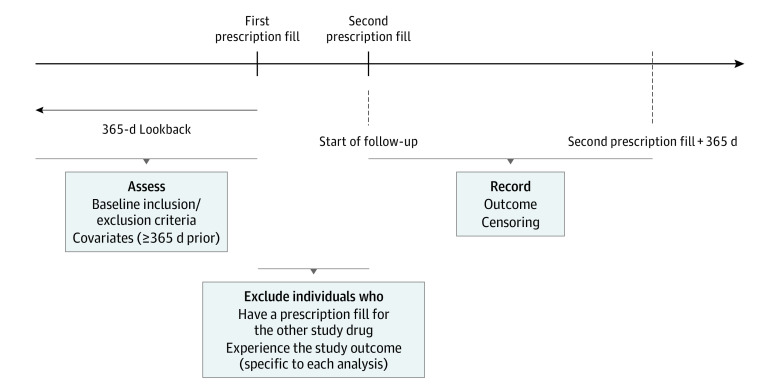
Study Design Diagram With Inclusion and Exclusion Criteria

We identified medications via prescription claims from Medicare part D claims. We considered 2 comparator drug classes (generic or branded): ABs (alfuzosin, doxazosin, tamsulosin, terazosin, silodosin, and prazosin) and 5-ARIs (finasteride and dutasteride) (eTable 2 in Supplement 1 and eTable 28 in Supplement 2). Medication initiation was defined as a prescription fill after January 1, 2008, without fills 12 months or longer prior. To minimize exposure misclassification due to nonadherence, we required individuals to refill the study drug 30 days or less after finishing their first prescription. We excluded individuals who filled a prescription for the other drug class or experienced an outcome between the first and second fill. Follow-up began at the second prescription fill to ensure equal follow-up across treatment groups (Figure 1).

### Outcomes

We identified study outcomes using algorithms with high specificity (93%-98%) or positive predictive value (>95%).^10,11,12,13,14,15,16^ Outcomes were hospitalization for heart failure (HF), composite major adverse cardiovascular events (MACE; hospitalization for stroke, MI, or death), composite MACE or HF hospitalization, and death. Hospitalization outcomes were identified from the in-patient record only (eTable 3 in Supplement 1). Death was identified using the Medicare Master Beneficiary Summary File: National Death Index Segment.^17^ To ensure comparable outcome identification across the transition from *International Classification of Diseases, Ninth Revision *(*ICD-9*) to *International Statistical Classification of Diseases and Related Health Problems, Tenth Revision *(*ICD-10*) codes (October 2015), we visually assessed monthly prevalence estimates for each outcome among all Medicare enrollees from 2013 to 2017 (eFigures 1-3 in Supplement 1).

### Covariates

Confounders were identified through a directed acyclic graph (eFigure 4 and eTable 4 in Supplement 1).^18,19,20^ Variables were identified based on claims 12 months or less prior to new use. They included age, race and Hispanic ethnicity, calendar year of new use, acute urinary retention, tobacco use, obesity, coronary heart disease, HF hospitalization, chronic kidney disease, chronic obstructive pulmonary disease [COPD], hypercholesterolemia, MI hospitalization, stroke hospitalization, diabetes, percutaneous coronary intervention, coronary artery bypass graft, atherosclerosis, antihypertensives (angiotensin-converting enzyme inhibitors, angiotensin receptor blockers, β blockers, calcium channel blockers, and diuretics), antidiabetics (dipeptidyl peptidase IV inhibitors, glucagon-like peptide 1 agonists, long-acting insulin, short-acting insulin, sodium-glucose cotransporter-2 inhibitors, sulfonylureas, and thiazolidinediones), other medications (anticoagulants, opioids, nicotine or varenicline, and statins), and components of the Faurot Frailty Index.^21,22,23^

Medications were identified using National Drug Codes. Other variables were identified using *ICD-9* and *ICD-10* diagnosis and procedure codes, *Current Procedural Terminology* codes, Healthcare Common Procedure Coding System codes, and administrative data (eTables 29-65 in Supplement 2). Race and ethnicity were measured as a single ethnoracial variable via beneficiary enrollment file information; we used a classification derived by the Research Triangle Institute.^24,25,26^ Categories included American Indian or Alaska Native, Asian or Pacific Islander, Black, Hispanic, non-Hispanic White, other (race not within other categories), and unknown. Combined race and ethnicity was included in our analyses as a proxy for processes of marginalization impacting health, not as a biological construct.^18,27,28^

### Statistical Analysis

We conducted the observational analogue of an intention-to-treat analysis.^29,30,31^ Treatment was determined at drug initiation, and patients were retained in that group over follow-up, regardless of treatment changes. Explicitly, we did not censor patients at treatment discontinuation or switching. We thus aimed to estimate the effect of initiating ABs vs 5-ARIs on adverse outcomes; however, because these are nonrandomized, secondary data and we cannot confirm that causal criteria (eg, no confounding) were met, we report associations throughout. For confounding, we calculated propensity scores (PSs) using logistic regression, modeling the probability of treatment with ABs vs 5-ARIs dependent on measured confounders (eTable 5 in Supplement 1). Stabilized inverse probability of treatment weights (IPTWs) were calculated as the marginal probability of a patient’s true treatment divided by the individual’s PS (or 1 minus the patient’s PS among initiators of 5-ARIs).^32,33,34^ We assessed covariate balance before and after IPTW using absolute standardized mean differences, with those ≤0.1 indicating adequate balance.

Patients were censored only at Medicare Parts A or B disenrollment. To account for this, we calculated stabilized inverse probability of censoring weights (IPCWs),^35^ using pooled logistic regression to estimate an individual’s probability of not being censored at each quintile of follow-up, dependent on treatment and factors associated with censoring (using the same variables as the PS model). Stabilized IPCWs were estimated as the probability of not being censored, dependent on treatment, divided by an individual’s probability of not being censored, dependent on treatment and factors associated with censoring.

We estimated cumulative incidence, risk ratios (RRs), and risk differences (RDs) from the second prescription fill through 1 year of follow-up using Kaplan-Meier and Aalen-Johansen estimators for mortality and nonmortality outcomes, respectively. Point estimates and CIs were calculated using nonparametric bootstrapping, drawing 500 random samples with replacement. RR and RD estimates and their SDs were calculated as the mean and standard error of point estimates across 500 samples. We calculated 2-sided 95% CIs, which when compared with the null value, indicate 2-sided statistical significance at *P* < .05. However, the goal of this study was to estimate effect sizes.^36,37,38,39,40^ To understand the magnitude of our exposure-outcome association, we calculated the number needed to harm (NNH) as the inverse of the RD estimate for outcomes with 95% CIs that did not overlap with 0. We plotted cumulative incidence curves in the nonbootstrapped sample. Furthermore, we evaluated treatment discontinuation and switching patterns to understand treatment persistence. Analyses were conducted using SAS statistical software version 9.4 (SAS Institute). Data were analyzed from January 2007 through December 2019; follow-up started January 2008.

#### A Priori Sensitivity Analyses

First, we used asymmetric propensity score trimming to understand the robustness of study results against uncontrolled confounding.^41,42^ Second, to understand residual confounding by hypertension, we repeated primary analyses considering only the ABs tamsulosin and silodosin, which are not indicated to treat hypertension.^43^ Third, to investigate our BPH definition, we required patients have 1 or more BPH diagnosis codes within 180 days prior to new use and compared cohort characteristics with those of the original population. Fourth, to assess confounding by BPH severity, we additionally controlled for days since the first-recorded BPH diagnosis code and number of BPH diagnoses during the 12-month lookback period. Fifth, to assess potential confounding by being a poor candidate for prostatectomy, we repeated analyses among a population of patients without prior anticoagulant use.^44^ Sixth, we limited our study period to start follow-up after October 1, 2015, so that all outcomes would be captured via *ICD-10* codes.

#### Post Hoc Sensitivity Analyses

First, we evaluated hospitalization for injury or poisoning as a negative control outcome (eTable 6 in Supplement 1).^45,46,47^ Second, we conducted a quantitative bias analysis to investigate residual confounding by misclassified obesity and smoking (eMethods in Supplement 1).^48,49,50^ Third, we repeated primary analyses among patients with 2 or more outpatient or 1 or more inpatient claims with a BPH diagnosis. Fourth, to investigate exposure-outcome associations among patients with a poor cardiovascular profile, we repeated the primary analysis among patients with 1 or more inpatient hospitalizations for MI, stroke, or HF within 12 months prior to cohort entry. Finally, to investigate confounding by socioeconomic status, we repeated primary analyses additionally controlling for eligibility for Medicaid dual enrollment^51^ and the Medicare Part D low-income subsidy.^52^

## Results

Among 189 868 older adult males, there were 163 829 initiators of ABs (86.3%; mean [SD] age, 74.6 [6.2] years; 579 American Indian or Alaska Native [0.4%], 5890 Asian or Pacific Islander [3.6%], 9179 Black [5.6%], 10 610 Hispanic [6.5%], and 133 510 non-Hispanic White [81.5%]) and 26 039 initiators of 5-ARIs (13.7%; mean [SD] age, 75.3 [6.4] years; 76 American Indian or Alaska Native [0.3%], 827 Asian or Pacific Islander [3.2%], 1339 Black [5.1%], 1656 Hispanic [6.4%], and 21 605 non-Hispanic White [83.0%]) (Table 1; eFigure 5 in Supplement 1). Tamsulosin was the most frequently filled AB; 141 398 initiators of ABs used a selective-type AB (86.3%) (eFigure 6 in Supplement 1). In the unweighted cohort, initiators of ABs had a higher prevalence than initiators of 5-ARIs of some cardiovascular disease risk factors, such as prior HF hospitalization (12 051 patients [7.4%] vs 1439 patients [5.5%]), prior stroke hospitalization (5595 patients [3.4%] vs 612 patients [2.9%]), and diabetes (54 347 patients [33.2%] vs 7530 patients [28.9%]). Initiators of 5-ARIs were older with a higher prevalence of hypercholesterolemia than initiators of ABs (81 013 patients [49.4%] vs 14 971 patients [57.5%]). Patients were well-balanced on measured covariates after using IPTWs (eFigures 7 and 8 and eTable 7 in Supplement 1). By 1 year, 96 492 initiators of ABs (58.9%) and 14 383 initiators of 5-ARIs (55.2%) had discontinued treatment, 18 223 initiators of ABs (11.1%) had filled a prescription for a 5-ARI, and 4973 initiators of 5-ARIs (19.1%) had filled a prescription for an AB (eFigure 9, eTable 8, and eTable 9 in Supplement 1).

**Table 1.** Distribution of Covariates at Treatment Initiation^a^

Covariate	Unweighted cohort^b^			IPTW cohort^b^		
	Patients initiating treatment, No. (%) (N = 189 868)		Absolute SMD	Patients initiating treatment, % (95% CI)^c^		Absolute SMD
	AB (n = 163 829)	5-ARI (n = 26 039)		AB	5-ARIα-reductase inhibitor initiators	
Age, median (IQR), y	73.0 (69.0-79.0)	74.0 (70.0-80.0)	0.117	74.0 (69.0-79.0)	74.0 (69.0-79.0)	0.016
Calendar year, median (IQR)	2014.0 (2011.0-2017.0)	2013.0 (2010.0-2016.0)	0.344	2014.0 (2011.0-2017.0)	2014.0 (2011.0-2017.0)	0.004
Race and ethnicity						
American Indian or Alaska Native	579 (0.4)	76 (0.3)	0.011	0.3 (0.3-0.4)	0.4 (0.3-0.5)	0.007
Asian or Pacific Islander	5890 (3.6)	827 (3.2)	0.023	3.5 (3.5-3.6)	3.6 (3.4-3.9)	0.005
Black or African American	9179 (5.6)	1339 (5.1)	0.020	5.5 (5.4-5.7)	5.8 (5.4-6.1)	0.010
Hispanic	10 610 (6.5)	1656 (6.4)	0.005	6.5 (6.3-6.6)	6.6 (6.2-6.9)	0.005
Non-Hispanic White	133 510 (81.5)	21 605 (83.0)	0.039	81.7 (81.5-81.9)	81.2 (80.6-81.8)	0.013
Other	1872 (1.1)	250 (1.0)	0.018	1.1 (1.1-1.2)	1.1 (1.1-1.3)	0.002
Unknown	2189 (1.3)	286 (1.1)	0.022	1.3 (1.2-1.4)	1.3 (1.1-1.4)	0.002
Part D cost share eligibility^d^						
Full	4069 (2.5)	591 (2.3)	0.014	2.5 (2.4-2.6)	2.2 (2.0-2.3)	0.023
Partial	32 281 (19.7)	4080 (15.7)	0.106	19.6 (19.4-19.8)	17.3 (16.8-17.8)	0.058
Not eligible	127 479 (77.8)	21 368 (82.1)	0.106	77.9 (77.7-78.2)	80.5 (80.0-81.1)	0.064
Medicaid dual eligibility^d^						
Full	6692 (4.1)	826 (3.2)	0.049	4.1 (4.0-4.1)	3.6 (3.3-3.8)	0.025
Partial	24 367 (14.9)	3103 (11.9)	0.087	14.8 (14.6-14.9)	13.1 (12.7-13.6)	0.047
Not eligible	132 770 (81)	22 110 (84.9)	0.103	81.2 (81.0-81.4)	83.3 (82.8-83.8)	0.055
Acute urinary retention	36 514 (22.3)	4574 (17.6)	0.118	21.6 (21.4-21.8)	21.5 (21-22.1)	0.002
Coronary heart disease	67 225 (41.0)	10 443 (40.1)	0.019	40.9 (40.7-41.2)	41.1 (40.5-41.8)	0.005
Hospitalization due to heart failure	12 051 (7.4)	1439 (5.5)	0.075	7.1 (7.0-7.2)	7.3 (6.9-7.8)	0.009
Prevalent heart failure^d^^,^^e^	20 523 (12.5)	2828 (10.9)	0.052	12.3 (12.1-12.5)	12.8 (12.3-13.2)	0.014
Chronic kidney disease	48 123 (29.4)	6208 (23.8)	0.125	28.6 (28.4-28.8)	28.9 (28.3-29.5)	0.007
COPD	39 662 (24.2)	5385 (20.7)	0.085	23.7 (23.5-23.9)	24.0 (23.4-24.6)	0.005
Hypercholesterolemia	81 013 (49.4)	14 971 (57.5)	0.162	50.6 (50.3-50.8)	50.6 (50.0-51.2)	0.001
Hospitalization due to MI	3339 (2.0)	427 (1.6)	0.030	2.0 (1.9-2.1)	2.1 (1.9-2.3)	0.005
Hospitalization due to stroke	5595 (3.4)	612 (2.4)	0.064	3.3 (3.2-3.4)	3.4 (3.1-3.7)	0.007
Percutaneous coronary intervention	2817 (1.7)	437 (1.7)	0.003	1.7 (1.7-1.8)	1.8 (1.6-2.0)	0.006
Coronary artery bypass graft surgery	1664 (1.0)	152 (0.6)	0.049	1.0 (0.9-1.0)	0.9 (0.8-1.1)	0.001
Tobacco use	30 344 (18.5)	3671 (14.1)	0.120	17.9 (17.7-18.1)	17.9 (17.4-18.4)	0.000
ACE inhibitor, any use	61 533 (37.6)	9258 (35.6)	0.042	37.3 (37.1-37.5)	37.3 (36.6-37.9)	0.000
ARB, any use	33 951 (20.7)	5072 (19.5)	0.031	20.6 (20.4-20.7)	20.7 (20.1-21.3)	0.003
β-blocker, any use	74 043 (45.2)	10 993 (42.2)	0.060	44.8 (44.5-45.0)	44.8 (44.2-45.5)	0.001
Peripheral vasodilator, any use	3029 (1.8)	609 (2.3)	0.034	1.9 (1.9-2.0)	2.0 (1.8-2.1)	0.003
Calcium channel blocker, any use	49 928 (30.5)	6877 (26.4)	0.090	29.9 (29.7-30.2)	29.9 (29.2-30.6)	0.000
Thiazide diuretic, any use	34 827 (21.3)	5250 (20.2)	0.027	21.1 (20.9-21.3)	20.9 (20.3-21.4)	0.006
Combination diuretic, any use	18 052 (11.0)	2920 (11.2)	0.006	11.0 (10.9-11.2)	10.9 (10.5-11.3)	0.004
Potassium sparing diuretic, any use	9191 (5.6)	1391 (5.3)	0.012	5.6 (5.5-5.7)	5.7 (5.3-6.0)	0.004
Loop diuretic, any use	25 547 (15.6)	3593 (13.8)	0.051	15.4 (15.2-15.5)	15.6 (15.2-16.1)	0.008
Other diuretics, any use	3517 (2.1)	453 (1.7)	0.029	2.1 (2.0-2.2)	2.1 (1.9-2.3)	0.000
Anticoagulant use						
No fill	141 194 (86.2)	22 111 (84.9)	0.036	86.0 (85.8-86.2)	85.9 (85.4-86.3)	0.004
1 Fill	4223 (2.6)	532 (2.0)	0.036	2.5 (2.4-2.6)	2.5 (2.3-2.8)	0.000
≥2 Fills	18 412 (11.2)	3396 (13.0)	0.055	11.5 (11.3-11.7)	11.6 (11.2-12.1)	0.005
Opioid use						
No fill	97 222 (59.3)	17 178 (66.0)	0.137	60.3 (60.0-60.5)	60.3 (59.6-61.0)	0.000
1 Fill	26 625 (16.3)	3958 (15.2)	0.029	16.1 (15.9-16.3)	16.0 (15.5-16.6)	0.002
≥2 Fills	39 982 (24.4)	4903 (18.8)	0.136	23.6 (23.4-23.9)	23.7 (23.1-24.3)	0.001
Nicotine or varenicline, any use	758 (0.5)	106 (0.4)	0.008	0.5 (0.4-0.5)	0.5 (0.4-0.6)	0.001
Statin, any use	96 595 (59.0)	14 652 **(**56.3)	0.054	58.6 (58.3-58.8)	58.4 (57.8-59.0)	0.003
Diabetes	54 347 (33.2)	7530 (28.9)	0.092	32.6 (32.4-32.8)	32.8 (32.2-33.5)	0.005
DPP-4i	6876 (4.2)	840 (3.2)	0.051	4.1 (4.0-4.2)	4.0 (3.8-4.3)	0.001
GLP-1	1626 (1.0)	164 (0.6)	0.040	0.9 (0.9-1.0)	1.0 (0.8-1.1)	0.002
Long-acting insulin						
No fill	151 982 (92.8)	24 626 (94.6)	0.074	93.0 (92.9-93.1)	92.9 (92.5-93.2)	0.005
1 Fill	1666 (1.0)	183 (0.7)	0.034	1.0 (0.9-1.0)	1.0 (0.8-1.1)	0.001
≥2 Fills	10 181 (6.2)	1230 (4.7)	0.066	6.0 (5.9-6.1)	6.1 (5.8-6.5)	0.006
Short-acting insulin						
No fill	157 738 (96.3)	25 320 (97.2)	0.054	96.4 (96.3-96.5)	96.3 (96.0-96.6)	0.006
1 Fill	1713 (1.0)	186 (0.7)	0.035	1.0 (1.0-1.0)	1.0 (0.8-1.2)	0.001
≥2 Fills	4378 (2.7)	533 (2.0)	0.041	2.6 (2.5-2.7)	2.7 (2.4-2.9)	0.006
SGLT-2i, any Fill	1144 (0.7)	108 (0.4)	0.038	0.7 (0.6-0.7)	0.7 (0.5-0.8)	0.002
Sulfonylureas, any fill	18 053 (11.0)	2366 (9.1)	0.064	10.8 (10.6-10.9)	10.7 (10.3-11.2)	0.000
TZD, any fill	4952 (3.0)	784 (3.0)	0.001	3.0 (2.9-3.1)	3.0 (2.8-3.3)	0.001
Atherosclerosis or peripheral vascular disease	45 604 (27.8)	8020 (30.8)	0.065	28.3 (28.0-28.5)	28.5 (27.9-29.1)	0.006
Obesity	18 951 (11.6)	1941 (7.5)	0.141	11.0 (10.9-11.2)	11.1 (10.6-11.6)	0.004

Abbreviations: 5-ARI, α-reductase inhibitor initiator; AB, α-blocker; ACE, angiotensin-converting enzyme; ARB, angiotensin receptor blocker; IPTW, inverse probability of treatment weight; COPD, chronic obstructive pulmonary disease; DPP-4i, dipeptidyl peptidase 4; GLP-1, glucagon-like peptide-1; MI, myocardial infarction; SGLT-2i, sodium-glucose cotransporter 2 inhibitor; SMD, standardized mean difference; TZD, thiazolidinedione.

^a^ Distribution is given in the primary cohort after trimming nonoverlapping regions of the propensity score distributions across 2 treatment groups. Results are presented before and after applying IPTWs.

^b^ Patients who experienced heart failure or a major adverse cardiovascular event between their first and second prescription fill are included. The sum of the standardized weight among the AB treatment group was 163 840, and the sum among the 5-ARI group was 26 009.

^c^ CIs were calculated using the proc surveymeans procedure in SAS statistical software using bootstrapping (with 250 resamples) to account for interdependence caused by the use of IPTWs.

^d^ These variables were not included in the primary propensity score model.

^e^ Identified as 2 or more outpatient or 1 or more inpatient claims with a diagnosis code for heart failure in any position.

**Figure 2.**
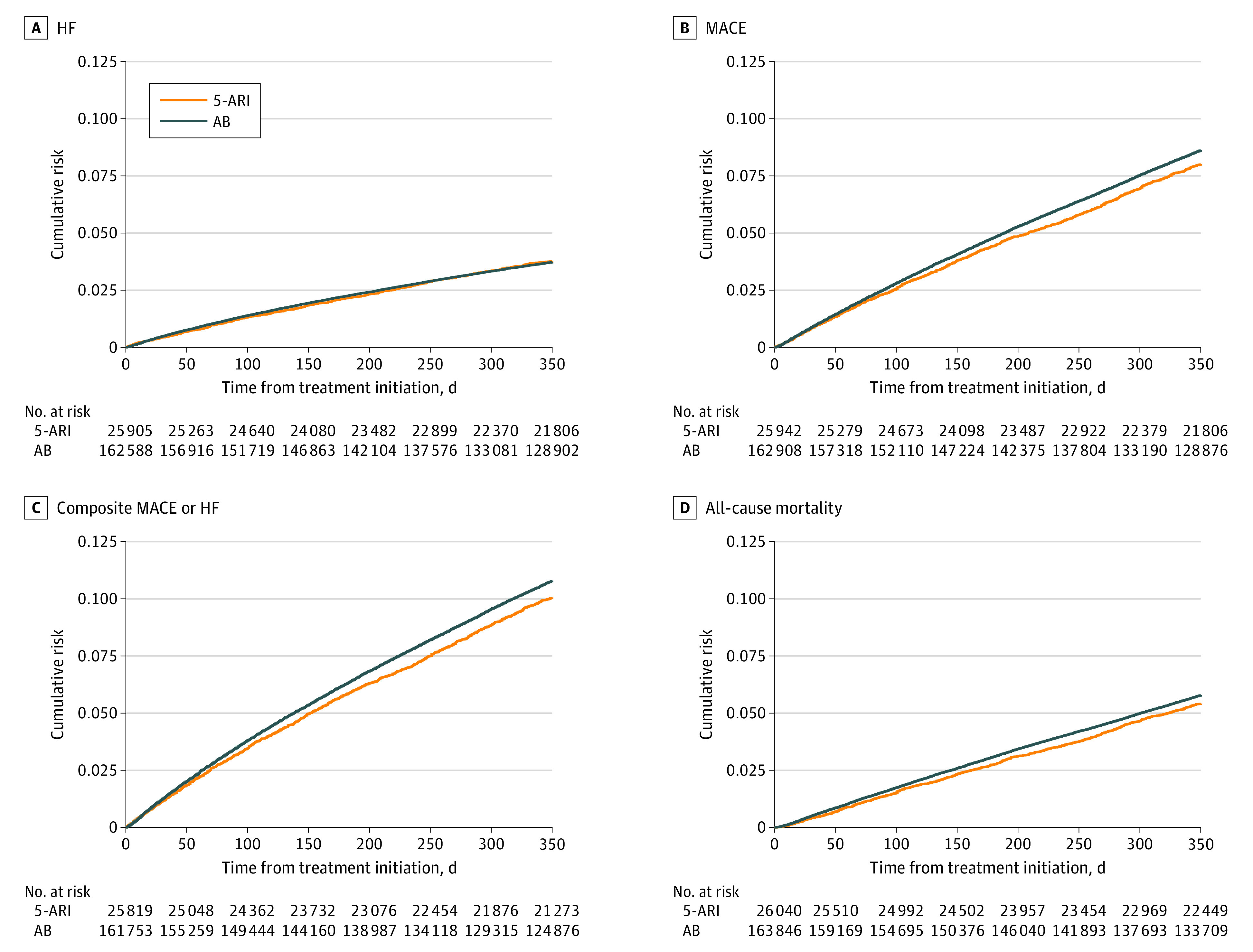
Cumulative Incidence for Primary Outcomes Outcomes were A, hospitalization for heart failure (HF), B, major adverse cardiovascular events (MACE), C, composite MACE or hospitalization for HF, and D, all-cause mortality. 5-ARI indicates 5-α reductase inhibitor; AB, α-blocker.

IPCW-adjusted 1-year risks of primary outcomes were low. Among initiators of ABs and 5-ARIs, 3.90% (95% CI, 3.80% to 4.00%) and 3.29% (95% CI, (3.07% to 3.52%) experienced HF hospitalization, 9.07% (95% CI, 8.93% to 9.21%) and 7.38% (95% CI, 7.04% to 7.72%) experienced MACE, 11.30% (95% CI, 11.14% to 11.45%) and 9.26% (95% CI, 8.89% to 9.63%) experienced composite MACE and HF hospitalization, and 6.11% (95% CI, 5.99% to 6.22%) and 4.95% (95% CI, 4.67% to 5.23%) died, respectively. These risks corresponded to relatively high estimates of RR (range, 1.18 [95% CI, 1.10 to 1.27] for HF hospitalization to 1.23 [95% CI, 1.17 to 1.29] for MACE and 1.23 [95% CI, 1.16 to 1.31] for deaths) and RD per 1000 individuals (range, 6.02 [95% CI, 3.51 to 8.53] for HF hospitalization to 20.38 [95% CI, 16.38 to 24.38] for composite MACE or HF hospitalization) for the AB vs 5-ARI group, but they were attenuated after IPTW. IPTW- and ICPW-adjusted estimates for RR and RD per 1000 individuals for HF hospitalization were 0.99 (95% CI, 0.92 to 1.07) and –0.23 (95% CI, −3.20 to 2.73), respectively, for AB vs 5-ARI groups (Figure 2; Table 2). For MACE, the risk was 8.95% (95% CI, 8.81% to 9.09%) for ABs and 8.32% (95% CI, 7.92% to 8.72%) for 5-ARIs, and the estimated RR and RD per 1000 individuals were 1.08 (95% CI, 1.02 to 1.13) and 6.26 (95% CI, 2.15 to 10.37; NNH = 160 individuals [95% CI, 97 to 466 individuals]), respectively, for AB vs 5-ARI groups. For composite MACE or HF hospitalization, the RR was 1.07 (95% CI, 1.03 to 1.12) and the RD per 1000 individuals was 7.40 (95% CI, 2.88 to 11.93; NNH = 136 individuals [95% CI, 84 to 348 individuals]) for AB vs 5-ARI groups. Finally, for mortality, estimates of RR and RD per 1000 individuals were 1.07 (95% CI, 1.01 to 1.14) and 3.85 (95% CI, 0.40 to 7.29; NNH = 260 individuals [95% CI, 138 to 2500 individuals]), respectively, for AB vs 5-ARI groups. These estimates align with 1024 additional MACE, 1204 composite MACE and HF hospitalizations, and 631 deaths over 1 year among initiators of ABs vs 5-ARIs.

**Table 2.** Association of Treatment With Study Outcomes^a^

Study outcome	Non-IPTW estimates				IPTW estimates			
	Risk, % (95% CI)		RD per 1000 (95% CI)	RR (95% CI)	Risk, % (95% CI)		RD per 1000 (95% CI)	RR (95% CI)
	AB	5-ARI			AB	5-ARI		
Hospitalization for HF	3.90 (3.80 to 4.00)	3.29 (3.07 to 3.52)	6.02 (3.51 to 8.53)	1.18 (1.10 to 1.27)	3.83 (3.74 to 3.93)	3.86 (3.58 to 4.14)	−0.23 (−3.20 to 2.73)	0.99 (0.92 to 1.07)
MACE outcomes	9.07 (8.93 to 9.21)	7.38 (7.04 to 7.72)	16.90 (13.31 to 20.50	1.23 (1.17 to 1.29)	8.95 (8.81 to 9.09)	8.32 (7.92 to 8.72)	6.26 (2.15 to 10.37)	1.08 (1.02 to 1.13)
Composite MACE or hospitalization for HF	11.30 (11.14 to 11.45)	9.26 (8.89 to 9.63)	20.38 (16.38 to 24.38)	1.22 (1.17 to 1.27)	11.14 (10.99 to 11.30)	10.40 (9.97 to 10.84)	7.40 (2.88 to 11.93)	1.07 (1.03 to 1.12)
Death from any cause	6.11 (5.99 to 6.22)	4.95 (4.67 to 5.23)	11.54 (8.48 to 14.61)	1.23 (1.16 to 1.31)	6.03 (5.91 to 6.14)	5.64 (5.31 to 5.97)	3.85 (0.40 to 7.29)	1.07 (1.01 to 1.14)

Abbreviations: 5-ARI, 5α-reductase inhibitor; AB, α-blocker; HF, heart failure; IPTW, inverse probability of treatment weight; MACE, major adverse cardiovascular event; RD, risk difference; RR, risk ratio.

^a^ Results are among the primary study population after trimming nonoverlapping regions of the propensity score distributions comparing initiators of ABs vs 5-ARIs. Results are presented with and without IPTWs; all results incorporate inverse probability of censoring weights.

Associations were consistent after asymmetric propensity score trimming (eTable 10 in Supplement 1) and after restricting ABs to tamsulosin and silodosin (eTables 11 and 12 in Supplement 1). The population identified when requiring 1 or more BPH diagnosis codes within 180 days prior to study drug initiation was comparable to the primary cohort (eTable 13 in Supplement 1), as were treatments (eFigure 10 in Supplement 1). Associations were attenuated when additionally controlling for indicators of BPH severity (eTable 14 in Supplement 1). Associations were consistent with primary results after excluding patients with prior anticoagulant use (eTable 15 in Supplement 1). Similarly, results were comparable, although less precise, when restricting to new-use episodes on or after October 1, 2015 (eTable 16 in Supplement 1).

In a post hoc sensitivity analysis evaluating risk of hospitalization for injury or poisoning, we found an RR of 1.06 (95% CI, 0.99 to 1.13) and an RD per 1000 individuals of 2.87 (95% CI, −0.42 to 6.16) for AB vs 5-ARI groups (eTable 17 in Supplement 1). Estimates from the quantitative bias analysis for misclassified smoking were attenuated from primary results for composite MACE and HF hospitalization (RR range, 0.94 to 1.03 for AB vs 5-ARI groups), although comparable when adjusted for misclassified obesity (RR range, 1.07 to 1.11 for AB vs 5-ARI groups) (eTable 18 in Supplement 1). Results were consistent among a population with 2 or more outpatient or 1 or more inpatient claims with a BPH diagnosis code (eTables 19 and 20 in Supplement 1). Estimates among patients with prior hospitalization for HF, MI, or stroke were too imprecise for meaningful conclusions (eTables 21 and 22 in Supplement 1). Finally, controlling for socioeconomic status yielded generally consistent results (eTable 23 in Supplement 1).

## Discussion

In this large cohort study of Medicare beneficiaries with BPH, we found that initiation of ABs compared with 5-ARIs was associated with an increased risk of death, MACE, and composite MACE and HF hospitalization. We did not identify an increased risk for HF hospitalization alone. IPCW-adjusted risks of these outcomes were low. To our knowledge, this is the largest study assessing cardiovascular risk in patients taking ABs and the first to focus on mortality and MACE among patients with BPH.

Residual confounding could contribute to these results. However, even a small difference in cardiovascular outcomes associated with ABs vs 5-ARIs would have substantial public health implications given how widely ABs are prescribed for BPH. Per our NNH estimate, for every 136 individuals (95% CI, 84-348 individuals) with BPH treated with ABs over 5-ARIs, we would expect 1 additional MACE or HF hospitalization within 1 year after initiation. To provide perspective, 163 829 individuals in our 20% Medicare sample (86.3%) were prescribed ABs over 5-ARIs. When this number is divided by the NNH of 136 individuals, this is potentially associated with 1205 additional events over 1 year. The importance of these findings may also be amplified because most patients with BPH require long-term medical therapy; thus, the risk of AB exposure could be cumulative. Furthermore, more than 5 million people filled a prescription for tamsulosin alone in the US in 2020, underscoring the potential magnitude of outcomes associated with these medications.^53^

Our findings regarding HF potentially contrast with those of ALLHAT, wherein a 2-fold risk of incident HF led to discontinuation of the nonselective AB group. Initial hypotheses regarding adverse effects of doxazosin pointed to less effective blood pressure control in this population compared with individuals receiving chlorthalidone. However, further analyses did not support this.^54^ Apparent differences between the 2 studies also could arise from ALLHAT’s examination of hospital and home-treated HF, whereas we considered only HF hospitalization. Furthermore, our cohort’s mean age was approximately 7 years older than that of participants in ALLHAT and included only male patients with an established diagnosis of BPH, whereas ALLHAT included male and female participants.

Our finding that ABs were not associated with HF hospitalization risk may also appear to contrast with an RR of 1.10 among users of ABs compared with those using 5-ARIs reported by Lusty et al.^55^ However, the Ontario study focused on new diagnosis of HF, whereas we studied HF hospitalization. Furthermore, they indexed patients into the study at the time of BPH diagnosis, introducing potential selection bias that our approach avoids.^9,56^ A meta-analysis of randomized trials among patients with indications for ABs by Sousa et al^57^ found that patients receiving ABs compared with those in active or placebo control groups had increased risk of acute HF (odds ratio = 1.78 [95% CI, 1.46-2.16]). They found no association with mortality (odds ratio = 1.10 [95% CI, 0.84-1.42]), although they did not analyze outcomes with respect to a relevant comparator group as we have done with patients taking 5-ARIs.

In contemporary practice, the most prescribed ABs for BPH are α-1A subtype-selective antagonists that have minimal impact on blood pressure.^58^ We found that increased risks of adverse cardiovascular outcomes were maintained when the exposure was limited to subtype-selective ABs in sensitivity analyses. This finding was unsurprising given that 141 398 ABs (86.3%) prescribed in our study were subtype selective. Collectively, these data align with the concept that activation of endogenous cardiac α1-A-ARs is cardioprotective, as demonstrated in preclinical studies.^3,59,60^

### Strengths and Limitations

This study has important strengths. Our source population was Medicare beneficiaries enrolled in Parts A and B fee-for-service plans. Given that approximately 54% of Medicare beneficiaries were enrolled in these plans in 2019,^61^ our study represents a substantial percentage of US adults aged 65 years or older. Moreover, we adhered to prespecified analyses and used rigorous methodology, such as IPTWs and IPCWs to account for systematic bias. We conducted a new-user, active comparator cohort study that minimized confounding by indication and avoided immortal time bias.^9,62^ Furthermore, we conducted an observational analog of the intention-to-treat analysis, minimizing bias due to informative censoring.^29,30,31^ Finally, our choice of active comparator is clinically relevant: ABs and 5-ARIs are first-line treatments for BPH, but adverse cardiac outcomes are hypothesized only with ABs.^3^ This choice ensured that our study addressed a pertinent clinical question and so may provide valuable insights into the safety of medications commonly prescribed for BPH management.

It is important to acknowledge several limitations in our study. First, like other studies using insurance claims data, this study is susceptible to variable misclassification. In sensitivity analyses, we found that our results were robust against poorly classified obesity but that if smoking operated as a confounder beyond what we have captured, residual confounding may explain our results. However, many studies have found that a large majority of individuals with COPD currently or previously smoked.^63,64,65^ Given that we controlled for COPD, there may be less residual confounding than this sensitivity analysis suggested. Second, despite substantial effort to control for confounding, some confounding may persist.^7^ Our results were consistent across multiple sensitivity analyses (eg, asymmetric PS trimming), although when controlling for indicators of BPH severity, for example, we found attenuated point estimates. Given the sizes of our association estimates, a post hoc negative control outcome analysis was uninformative (eAppendix in Supplement 1). Third, we evaluated only 1 year of follow-up; we were concerned about our ability to capture reasons for treatment changes (eg, BPH symptom severity),^35^ and treatment changes were common. Furthermore, we did not consider medication dosing given that clinicians often instruct patients to take a different number of the same pill dosage according to their BPH symptoms, which cannot be captured in claims. Fourth, this study may not be generalizable to all patients with BPH. We excluded beneficiaries enrolled in Medicare Advantage plans, required 2 or more medication fills, and included claims only through 2019. These limitations should not impact our study’s internal validity; however, any differences in the distribution of variables that modify the effect of ABs vs 5-ARIs on cardiovascular outcomes (ie, effect measure modifiers) will impact the transportability of our results to other populations.^66,67^

## Conclusions

This cohort study is the largest study of the association of ABs with cardiovascular events to date, to our knowledge. We found that new prescription of ABs compared with 5-ARIs was associated with a higher risk of all-cause mortality and MACE among patients with BPH. Although we present the most extensive analysis, to our knowledge, of the cardiovascular safety of ABs heretofore, further investigation with more detailed clinical data is warranted to guide ongoing clinical practice.

## Notes

Supplement 1. **eTable 1.** Diagnosis and Procedure Codes for Inclusion and Exclusion Criteria **eTable 2.** National Drug Codes Used to Identify Study Exposures **eTable 3.** Diagnoses Codes Used to Identify Study Outcomes **eTable 4.** Diagnosis Codes, Procedure Codes, and Drug Names Used to Identify Confounders and Their Operationalization **eFigure 1.** Month-Level Prevalence of Primary Outcome Definition for Inpatient Hospitalization for Heart Failure Over *ICD-9* to *ICD-10* Transition **eFigure 2.** Month-Level Prevalence of Primary Outcome Definition for Inpatient Hospitalization for Stroke Over *ICD-9* to *ICD-10* Transition **eFigure 3.** Month-Level Prevalence of Primary Outcome Definition for Inpatient Hospitalization for Myocardial Infarction Over *ICD-9* to *ICD-10* Transition **eFigure 4.** Directed Acyclic Graph Used to Identify Important Potential Confounders in This Study **eTable 5.** Logistic Regression Model Specification Used to Generate Propensity Scores and Inverse Probability of Censoring Weights in Primary Analyses **eTable 6.** Diagnosis Code List to Identify Hospitalization Due to Injury or Poisoning as Negative Control Outcome **eMethods.** Quantitative Bias Analysis for Smoking and Obesity **eFigure 5.** Diagram Demonstrating Flow of Study Patients and New-Use Episodes Through Inclusion and Exclusion Criteria **eFigure 6.** Annual Estimates of Proportion of New-Use Episodes Attributed to Each Study Drug in Primary Population **eFigure 7.** Propensity Score Distributions Prior to Trimming Patients **eFigure 8.** Propensity Score Distributions After Trimming Nonoverlapping Propensity Scores and Refitting Logistic Regression Model in Included Patient Population Without Bootstrapping **eTable 7.** Descriptive Statistics of Stabilized Inverse Probability of Treatment Weights in Primary Patient Population Without Bootstrapping **eFigure 9.** Histogram of No. Days From Second Fill Date Until Discontinuation, Fill for Other Drug Class, or Censoring **eTable 8.** Counts and Percentages of Medications Attributed to New-Use Episodes by Year in Primary Study Population **eTable 9.** Descriptive Statistics of Days From Second Prescription Fill Until Discontinuation, Filling Prescription for Other Study Drug, or Censoring by Initial Treatment and Amount of Follow-Up **eTable 10.** Primary Study Results After 1-y Follow-Up Among Population After Asymmetric Propensity Score Trimming **eTable 11.** Descriptive Table of Study Population Limited to New-Use Episodes by New Users of α-Blockers Attributed to Tamsulosin or Silodosin **eTable 12.** Primary Study Results After 1-y Follow-Up Limited to New-Use Episodes Among α-Blockers Attributed to Tamsulosin and Silodosin, Selective α-1A Adrenergic Receptor Antagonists **eTable 13.** Descriptive Statistics of Unweighted Patient Population With Benign Prostatic Hyperplasia Diagnosis in 180 d Prior to New-Use Episode **eFigure 10.** Distribution of Medication Fill at an Individual’s Index Date Into the Cohort Across Calendar Years **eTable 14.** Primary Study Results After 1-y Follow-Up Additionally Controlling for Indicators of Severity of Benign Prostatic Hyperplasia **eTable 15.** Study Results After Removing Patients With History of Anticoagulant Fill Within 1-y Prior to New-Use Episode **eTable 16.** Study Results Restricting to New-Use Periods on or After October 1, 2015 **eTable 17.** Study Estimates for Hospitalization for Injury or Poisoning as Negative Control Outcome **eTable 18.** Results from Quantitative Bias Analysis **eTable 19.** Descriptive Statistics of Study Population Restricted to Individuals With ≥2 Outpatient or ≥1 Inpatient Diagnosis Codes for Benign Prostatic Hyperplasia **eTable 20.** Study Results Among Patients With ≥2 Outpatient or ≥1 Inpatient Diagnosis Codes for Benign Prostatic Hyperplasia **eTable 21.** Descriptive Statistics of Study Population Restricted to Individuals With ≥1 Inpatient Hospitalization for Myocardial Infarction, Stroke, or Heart Failure Within 1 y Prior to Cohort Entry **eTable 22.** Study Results Among Patients With History of Hospitalization for Heart Failure, Myocardial Infarction, or Stroke Within 1 y Prior to New-Use of Study Medications **eTable 23.** Study Results Among Primary Study Population Additionally Adjusting for Dual Eligibility for Medicaid and Receipt of Medicare Part D Low-Income Subsidy **eAppendix.** Discussion on Limitations of Hospitalization for Injury or Poisoning as Negative Control Outcome Analysis
eReferences.

Supplement 2. **eTable 24.** Benign Prostatic Hyperplasia **eTable 25.** Chemotherapy **eTable 26.** Prostate Cancer **eTable 27.** Prostatectomy **eTable 28.** Exposure/Benign Prostatic Hyperplasia Treatment **eTable 29.** Acute Urinary Retention **eTable 30.** Diabetes **eTable 31.** Coronary Artery/Heart Disease **eTable 32.** Inpatient Hospitalization for Heart Failure **eTable 33.** Hypercholesterolemia **eTable 34.** Angiotensin II Receptor Blockers **eTable 35.** Calcium Channel Blockers **eTable 36.** β-Blockers **eTable 37.** Peripheral Vasodilators **eTable 38.** Thiazide Diuretic **eTable 39.** Angiotensin-Converting Enzyme Inhibitors **eTable 40.** Combination Diuretic **eTable 41.** Potassium-Sparing Diuretic **eTable 42.** Loop Diuretic **eTable 43.** Other Diuretic **eTable 44.** Inpatient Hospitalization for Myocardial Infarction **eTable 45.** Percutaneous Coronary Intervention Procedure **eTable 46.** Inpatient Hospitalization for Stroke/Brain Injury **eTable 47.** Atherosclerosis **eTable 48.** Chronic Kidney Disease **eTable 49.** Chronic Obstructive Pulmonary Disease **eTable 50.** Tobacco Use **eTable 51.** Anticoagulants **eTable 52.** Obesity **eTable 53.** Dipeptidyl Peptidase 4 Inhibitors **eTable 54.** Glucagon-Like Peptide-1 Agonists **eTable 55.** Long-Acting Insulin **eTable 56.** Short-Acting Insulin **eTable 57.** Sodium/Glucose Cotransporter-2 Inhibitors **eTable 58.** Sulfonylurea **eTable 59.** Thiazolidinediones **eTable 60.** Biguanide **eTable 61.** Opioids **eTable 62.** Statins **eTable 63.** Coronary Artery Bypass Graft Surgery **eTable 64.** Faurot Frailty Index Indicators **eTable 65.** Pneumonia

Supplement 3.
Data Sharing Statement
