# Supplementary material for: Cardiovascular Outcomes of α-Blockers vs 5-α Reductase Inhibitors for Benign Prostatic Hyperplasia
Source: JAMA Netw Open. 2023 Nov 14;6(11):e2343299. doi: 10.1001/jamanetworkopen.2023.43299 (PMC10646730; doi:10.1001/jamanetworkopen.2023.43299)
Supplement: Supplement 1. — eTable 1. Diagnosis and Procedure Codes for Inclusion and Exclusion Criteria eTable 2. National Drug Codes Used to Identify Study Exposures eTable 3. Diagnoses Codes Used to Identify Study Outcomes eTable 4. Diagnosis Codes, Procedure Codes, and Drug Names Used to Identify Confounders and Their Operationalization eFigure 1. Month-Level Prevalence of Primary Outcome Definition for Inpatient Hospitalization for Heart Failure Over ICD-9 to ICD-10 Transition eFigure 2. Month-Level Prevalence of Primary Outcome Definition for Inpatient Hospitalization for Stroke Over ICD-9 to ICD-10 Transition eFigure 3. Month-Level Prevalence of Primary Outcome Definition for Inpatient Hospitalization for Myocardial Infarction Over ICD-9 to ICD-10 Transition eFigure 4. Directed Acyclic Graph Used to Identify Important Potential Confounders in This Study eTable 5. Logistic Regression Model Specification Used to Generate Propensity Scores and Inverse Probability of Censoring Weights in Primary Analyses eTable 6. Diagnosis Code List to Identify Hospitalization Due to Injury or Poisoning as Negative Control Outcome eMethods. Quantitative Bias Analysis for Smoking and Obesity eFigure 5. Diagram Demonstrating Flow of Study Patients and New-Use Episodes Through Inclusion and Exclusion Criteria eFigure 6. Annual Estimates of Proportion of New-Use Episodes Attributed to Each Study Drug in Primary Population eFigure 7. Propensity Score Distributions Prior to Trimming Patients eFigure 8. Propensity Score Distributions After Trimming Nonoverlapping Propensity Scores and Refitting Logistic Regression Model in Included Patient Population Without Bootstrapping eTable 7. Descriptive Statistics of Stabilized Inverse Probability of Treatment Weights in Primary Patient Population Without Bootstrapping eFigure 9. Histogram of No. Days From Second Fill Date Until Discontinuation, Fill for Other Drug Class, or Censoring eTable 8. Counts and Percentages of Medications Attributed to New-Use Episodes by Year in [file jamanetwopen-e2343299-s001.pdf]

Supplemental Online Content

Zhang J, Latour CD, Olawore O, et al. Cardiovascular outcomes of α blockers vs 5-α reductase inhibitors for benign prostatic hyperplasia. *JAMA Netw Open*. 2023;6(11):e2343299. doi:10.1001/jamanetworkopen.2023.43299

eTable 1. Diagnosis and Procedure Codes for Inclusion and Exclusion Criteria

eTable 2. National Drug Codes Used to Identify Study Exposures

eTable 3. Diagnoses Codes Used to Identify Study Outcomes

eTable 4. Diagnosis Codes, Procedure Codes, and Drug Names Used to Identify Confounders and Their Operationalization

eFigure 1. Month-Level Prevalence of Primary Outcome Definition for Inpatient Hospitalization for Heart Failure Over *ICD-9* to *ICD-10* Transition

eFigure 2. Month-Level Prevalence of Primary Outcome Definition for Inpatient Hospitalization for Stroke Over *ICD-9* to *ICD-10* Transition

eFigure 3. Month-Level Prevalence of Primary Outcome Definition for Inpatient Hospitalization for Myocardial Infarction Over *ICD-9* to *ICD-10* Transition

eFigure 4. Directed Acyclic Graph Used to Identify Important Potential Confounders in This Study

eTable 5. Logistic Regression Model Specification Used to Generate Propensity Scores and Inverse Probability of Censoring Weights in Primary Analyses

eTable 6. Diagnosis Code List to Identify Hospitalization Due to Injury or Poisoning as Negative Control Outcome

eMethods. Quantitative Bias Analysis for Smoking and Obesity

eFigure 5. Diagram Demonstrating Flow of Study Patients and New-Use Episodes Through Inclusion and Exclusion Criteria

eFigure 6. Annual Estimates of Proportion of New-Use Episodes Attributed to Each Study Drug in Primary Population

eFigure 7. Propensity Score Distributions Prior to Trimming Patients

eFigure 8. Propensity Score Distributions After Trimming Nonoverlapping Propensity Scores and Refitting Logistic Regression Model in Included Patient Population Without Bootstrapping

eTable 7. Descriptive Statistics of Stabilized Inverse Probability of Treatment Weights in Primary Patient Population Without Bootstrapping

© 2023 Zhang J et al. *JAMA Network Open.*

eFigure 9. Histogram of No. Days From Second Fill Date Until Discontinuation, Fill for Other Drug Class, or Censoring

eTable 8. Counts and Percentages of Medications Attributed to New-Use Episodes by Year in Primary Study Population

eTable 9. Descriptive Statistics of Days From Second Prescription Fill Until Discontinuation, Filling Prescription for Other Study Drug, or Censoring by Initial Treatment and Amount of Follow-Up

eTable 10. Primary Study Results After 1-y Follow-Up Among Population After Asymmetric Propensity Score Trimming

eTable 11. Descriptive Table of Study Population Limited to New-Use Episodes by New Users of α-Blockers Attributed to Tamsulosin or Silodosin

eTable 12. Primary Study Results After 1-y Follow-Up Limited to New-Use Episodes Among α-Blockers Attributed to Tamsulosin and Silodosin, Selective α-1A Adrenergic Receptor Antagonists

eTable 13. Descriptive Statistics of Unweighted Patient Population With Benign Prostatic Hyperplasia Diagnosis in 180 d Prior to New-Use Episode

eFigure 10. Distribution of Medication Fill at an Individual's Index Date Into the Cohort Across Calendar Years

eTable 14. Primary Study Results After 1-y Follow-Up Additionally Controlling for Indicators of Severity of Benign Prostatic Hyperplasia

eTable 15. Study Results After Removing Patients With History of Anticoagulant Fill Within 1-y Prior to New-Use Episode

eTable 16. Study Results Restricting to New-Use Periods on or After October 1, 2015

eTable 17. Study Estimates for Hospitalization for Injury or Poisoning as Negative Control Outcome

eTable 18. Results from Quantitative Bias Analysis

eTable 19. Descriptive Statistics of Study Population Restricted to Individuals With ≥2 Outpatient or ≥1 Inpatient Diagnosis Codes for Benign Prostatic Hyperplasia

eTable 20. Study Results Among Patients With ≥2 Outpatient or ≥1 Inpatient Diagnosis Codes for Benign Prostatic Hyperplasia

eTable 21. Descriptive Statistics of Study Population Restricted to Individuals With ≥1 Inpatient Hospitalization for Myocardial Infarction, Stroke, or Heart Failure Within 1 y Prior to Cohort Entry

eTable 22. Study Results Among Patients With History of Hospitalization for Heart Failure, Myocardial Infarction, or Stroke Within 1 y Prior to New-Use of Study Medications

eTable 23. Study Results Among Primary Study Population Additionally Adjusting for Dual Eligibility for Medicaid and Receipt of Medicare Part D Low-Income Subsidy

© 2023 Zhang J et al. *JAMA Network Open.*

eAppendix. Discussion on Limitations of Hospitalization for Injury or Poisoning as Negative Control Outcome Analysis

eReferences.

This supplemental material has been provided by the authors to give readers additional information about their work.

Table S1. Diagnosis and procedure codes for inclusion and exclusion criteria

Variable	Description	ICD-9-CM	ICD-10-CM	HCPCS/CPT	NDC
Benign Prostatic Hyperplasia	Diagnosis of BPH, ≤12 months prior to initiation of BPH therapy	600; 600.01; 600.11, 600.2; 600.21, 600.9; 600.91	N40; N40.1; N40.2; N40.3; R39.9	N/A	N/A
Prostate Cancer	All available lookback for diagnoses	185; 233.4; 326.5; V10.46	C61; D07.5; D40.0; Z85.46	N/A	N/A
Prostatectomy	All available lookback for occurrence	N/A	N/A	00865; 00908; 52601; 52612; 52614; 55801; 55810; 55812; 55815; 55821; 55831; 55840; 55842; 55845; 55866; 52620; 52630	N/A
Current Chemotherapy	Occurrence ≤ 6 months after initiation of therapy	**ICD-9-DX**: V58.1; V58.11; V58.12;<br><br>**ICD-9-PR**: 00.10; 17.70; 99.25; 99.28	Z51.1; Z51.11; Z51.12;	C8953; C8954; C8955; G0355; G0357; G0358; G0359; G0360; G0361; G0362; G0370; J7150; Q0083; Q0084; Q0085; S5019; S5020; S9329; S9330; S9331; S9425; C1166; C1167; C1178; C9012; C9110; C9127; C9205; C9207; C9213; C9214; C9215; C9217; C9218; C9235; C9257; C9262; C9414; C9415; C9417; C9418; C9419; C9420; C9421; C9422; C9423; C9424; C9425;	4110013; 4110020; 4110022; 4110051; 4110116; 4110150; 5450704; 5450705; 5450707; 5450709; 5450723; 5450791; 15050301; 15050302; 15050401; 15309145; 54412925; 54413025; 54455015; 54455025; 54808925; 54813025; 54855003; 54855005; 54855006; 54855007; 54855010; 54855025; 81004535; 85124401; 85124402; 85124801;

© 2023 Zhang J et al. *JAMA Network Open.*

				C9426; C9427; C9429; C9431; C9432; C9433; C9437; C9440; J0594; J0894; J8510; J8520; J8521; J8530; J8560; J8565; J8600; J8610; J8700; J8705; J8999; J9000; J9001; J9010; J9017; J9020; J9025; J9027; J9033; J9035; J9040; J9041; J9045; J9050; J9055; J9060; J9062; J9065; J9070; J9080; J9090; J9091; J9092; J9093; J9094; J9095; J9096; J9097; J9098; J9100; J9110; J9120; J9130; J9140; J9150; J9151; J9170; J9171; J9178; J9180; J9181; J9182; J9185; J9190; J9200; J9201; J9206; J9207; J9208; J9211; J9230; J9245; J9250; J9260; J9261; J9263; J9264; J9265; J9266; J9268; J9270; J9280; J9290; J9291; J9293; J9300; J9303; J9305; J9307; J9310; J9315; J9320; J9328; J9330; J9340; J9350; J9351; J9355; J9357; J9360; J9370; J9375;	85124802; 85125201; 85125202; 85125901; 85125902; 173004535; 173071325; 182153901; 182153995; 364249901; 364249936; 378001401; 378001450; 378326694; 536399801; 536399836; 555057202; 555057235; 555057245; 555057246; 555057247; 555057248; 555057249; 555092701; 555092801; 555092901; 555094501; 603449921; 677161001; 781107601; 781107636; 904174960; 904174973; 51079067005; 51079096505; 51285050902; 54569571700;

© 2023 Zhang J et al. *JAMA Network Open.*

				J9380; J9390; J9999; Q2017; Q2024; S0087; S0088; S0115; S0116; S0172; S0176; S0178; S0182; 0519F; 36823; 51720;; 61517; 95990; 95991; 96400; 96401; 96402; 96405; 96406; 96408; 96409; 96410; 96411; 96412; 96413; 96414; 96415; 96416; 96417; 96420; 96422; 96423; 96425; 96440; 96445; 96446; 96450; 96520; 96521; 96522; 96523; 96530; 96542; 96545	54868414300; 54868414301; 54868414302; 54868414303; 54868526000; 54868526001; 54868526002; 54868526003; 54868526004; 54868526005; 54868526006; 54868526007; 54868526008; 54868526009; 59911587401; 62701094036; 62701094099

Abbreviations: CPT=Current Procedural Terminology, Dx = Diagnosis; HCPCS=Healthcare Common Procedure Coding System; ICD-9-CM=International Classification of Diseases, 9th Revision, Clinical Modification; ICD-10-CM: International Classification of Diseases, 10th Revision, Clinical Modification; NDC = National Drug Code; N/A=not applicable; PR = Procedure

© 2023 Zhang J et al. *JAMA Network Open.*

Table S2. National Drug Codes used to identify study exposures.

Generic Drug Name	Drug Type	NDC
Alfuzosin	Non alpha-1a selective blocker	13668-021; 29300-155; 43353-945; 57237-114; 64679-738; 63629-2353; 65862-249; 61919-349; 69097-844; 70934-306; 76282-302; 71335-1529
Doxazosin	Non alpha-1a selective blocker	0093-2067; 0093-2068; 0093-2069; 0093-2070; 0378-4021; 0378-4022; 0378-4024; 0378-4028; 0832-0356; 0832-0357; 0832-1358; 0832-1359; 0904-5522; 0904-5523; 0904-5524; 16729-211; 16729-213; 16729-414; 16729-415; 23155-092; 23155-093; 23155-094; 23155-095; 42291-258; 42291-259; 42291-260; 43063-739; 43353-646; 43353-739; 43353-740; 50090-0706; 50090-2153; 50090-3927; 50090-3987; 50090-4000; 50090-4004; 51079-958; 51079-959; 53808-1107; 55154-7996; 55289-022; 55289-600; 59762-2310; 59762-2320; 59762-2340; 59762-2380; 60429-953; 60429-954; 60429-955; 60429-956; 60505-0093; 60505-0094; 60505-0095; 60505-0096; 63629-7891; 66267-377; 67253-381; 67253-382; 68071-3298; 68071-4272; 68071-4273; 68071-4892; 68071-5153; 68084-836; 68084-851; 68084-862; 68382-783; 68382-784; 68382-785; 68382-786; 68788-7149; 68788-7328; 70518-0839; 70518-1560; 70518-1804; 70518-2183; 70771-1112; 70771-1113; 70771-1114; 70771-1115; 70934-362; 70934-366; 70934-728; 71335-0002; 71335-0309; 71335-0349; 71610-043; 71610-110; 71610-120; 71610-153; 71610-160; 71610-251; 71610-420; 71610-482; 72189-117
Terazosin	Non alpha-1a selective blocker	71610-510; 71610-503; 71610-478; 71335-0787; 70934-552; 70518-1875; 70518-0342; 70518-0074; 68788-9817; 68788-9816; 68788-9815; 67544-581; 67544-564; 67544-563; 67544-562; 67296-1264; 66267-389; 66267-325; 63629-5237; 63629-4525; 63629-2439; 63629-1414; 63187-620; 63187-456; 60760-316; 59746-386; 59746-385; 59746-383; 55700-750; 53002-1728; 53002-1727; 53002-1726; 53002-1250; 50268-767; 50268-766; 50268-765; 50268-764; 50090-2623; 50090-0659; 50090-0657; 49999-227; 42291-823; 42291-822; 42291-821; 42291-820; 24689-961; 24689-882; 24689-855; 24689-792; 0781-2054; 0781-2053; 0781-2052; 0781-2051; 0378-2268; 0378-2264; 0378-2260; 0378-1570
Prazosin	Non alpha-1a selective blocker	0093-4067; 0093-4068; 0093-4069; 0378-1101; 0378-2302; 0378-3205; 0904-7020; 0904-7021; 0904-7022; 43063-248; 43063-260; 50090-0379; 50090-1948; 50090-2845; 51079-630; 51079-631; 51079-632; 55154-8181; 55289-536; 59762-5310; 59762-5320; 59762-5350; 60429-971; 60429-972; 60429-973; 60687-572; 61919-910; 62559-580; 62559-581; 62559-582; 63629-6873; 63629-8325; 67046-208; 67046-209; 68084-996; 68084-997; 68788-7335; 68788-7447; 68788-7824;

© 2023 Zhang J et al. *JAMA Network Open.*

		68788-7849; 70518-1884; 70518-1895; 70518-2065; 70518-2177; 70518-2178; 70518-2705; 70954-019; 70954-020; 70954-021; 71335-1085; 71335-1401; 71335-1556; 71335-1724; 71610-046
Tamsulosin	Alpha-1a selective blocker	0228-2996; 0615-8055; 0781-2076; 0904-6401; 10370-169; 16714-713; 33342-159; 43063-264; 43063-725; 43063-947; 43353-013; 43353-019; 50090-3646; 50090-4906; 50268-740; 55154-7649; 55700-670; 57237-014; 58118-0598; 60723-056; 60760-450; 60760-598; 61919-298; 61919-703; 62756-160; 62756-160; 63187-358; 63187-371; 63187-469; 63629-6807; 63629-8135; 63739-567; 64679-516; 65841-695; 65862-598; 67046-721; 67296-0802; 67296-1155; 67296-1319; 67296-1646; 67296-1739; 67877-450; 68071-1840; 68071-4443; 68071-4872; 68071-5118; 68084-299; 68382-132; 68788-7050; 68788-7780; 70518-0179; 70518-1999; 70518-2052; 70518-2541; 70934-092; 70934-328; 70934-771; 71205-217; 71335-0398; 71335-0711; 71335-1538
Silodosin	Alpha-1a selective blocker	0781-2623; 0781-2624; 27241-144; 27241-145; 31722-635; 31722-636; 33342-384; 33342-385; 46708-405; 46708-406; 59651-095; 59651-096; 62332-405; 62332-406; 68180-740; 68180-741; 69238-1420; 69238-1421; 69539-052; 69539-053; 72205-009; 72205-010; 72789-090
Finasteride	5-alpha reductase inhibitor	0093-7355; 0904-6830; 16729-089; 16729-090; 17856-0090; 31722-525; 31722-526; 35573-400; 43598-303; 43598-390; 50090-1718; 50090-1936; 50090-3942; 50090-4648; 50090-4697; 50268-314; 55111-171; 55111-172; 55154-8083; 57237-061; 57237-062; 60687-428; 61919-733; 63187-265; 63187-863; 63629-8180; 65862-149; 65862-927; 67877-288; 67877-455; 68071-3094; 68071-3306; 68071-3354; 68071-3380; 68071-4167; 68071-4541; 68071-4808; 68382-074; 68645-541; 68788-6875; 68788-6976; 69097-112; 70518-0397; 70518-2287; 70518-2363; 70771-1152; 71335-0235; 71335-0281; 71335-1304; 71335-1530; 71335-1634; 71610-515; 71610-520; 71713-096; 71713-099; 76282-412; 76420-074
Dutasteride	5-alpha reductase inhibitor	0093-7355; 0904-6830; 16729-089; 16729-090; 17856-0090; 31722-525; 31722-526; 35573-400; 43598-303; 43598-390; 50090-1718; 50090-1936; 50090-3942; 50090-4648; 50090-4697; 50268-314; 55111-171; 55111-172; 55154-8083; 57237-061; 57237-062; 60687-428; 61919-733; 63187-265; 63187-863; 63629-8180; 65862-149; 65862-927; 67877-288; 67877-455; 68071-3094; 68071-3306; 68071-3354; 68071-3380; 68071-4167; 68071-4541; 68071-4808; 68382-074; 68645-541; 68788-6875; 68788-6976; 69097-112; 70518-0397; 70518-2287; 70518-2363; 70771-1152; 71335-0235; 71335-0281; 71335-1304; 71335-1530;

© 2023 Zhang J et al. *JAMA Network Open.*

		71335-1634; 71610-515; 71610-520; 71713-096; 71713-099; 76282-412; 76420-074

Abbreviations: ICD-9-CM=International Classification of Diseases, 9th Revision, Clinical Modification; ICD-10-CM: International Classification of Diseases, 10th Revision, Clinical Modification; NDC =National Drug Code
Explicitly, we included both branded and generic drugs of the same medication.

© 2023 Zhang J et al. *JAMA Network Open.*

Table S3. Diagnoses codes used to identify study outcomes.

Outcome	Description	ICD-9-CM	ICD-10-CM
In-patient hospital admission for heart failure	≥1 diagnosis code in the 1st or 2nd position of the discharge codes on an inpatient claim.	398.91; 428.xx; 402.01; 402.11; 402.91; 404.01; 404.03; 404.11; 404.13; 404.91; 404.93	I09.81; I11.0; I13.0; I13.2; I50; I50.1; I50.2; I50.20; I50.21; I50.22, I50.23, I50.3; I50.30; I50.31; I50.32; I50.33; I50.4; I50.40; I50.41; I50.42; I50.43; I50.9
In-patient hospital admission for stroke or brain injury	≥1 diagnosis code in the 1st or 2nd position of the discharge codes on an inpatient claim	430.xx; 431.xx; 433.xx; 434.xx; 436.xx; 437.xx; 438.xx	I60.xx; I61.xx; I63.xx; I67.xx; I69.xx
In-patient hospital admission for myocardial infarction	≥1 diagnosis code in the 1st or 2nd position of the discharge codes on an inpatient claim	410.xx	I21.xx; I22.x

Abbreviations: CPT=Current Procedural Terminology, HCPCS=Healthcare Common Procedure Coding System; ICD-9-CM=International Classification of Diseases, 9th Revision, Clinical Modification; ICD-10-CM: International Classification of Diseases, 10th Revision, Clinical Modification; N/A=not applicable

© 2023 Zhang J et al. *JAMA Network Open.*

**Table S4.** Diagnosis codes, procedure codes, and drug names used to identify confounders for the study as well as their operationalization.		
Variable	Description	Codes and Drug Names
Acute urinary retention	≥1 code, inpatient or outpatient claims	ICD-9-CM Dx: 788.2x<br><br>ICD-10-CM Dx: R33.x
Diabetes mellitus	≥2 codes on inpatient outpatient claims	ICD-9-CM Dx: 249.*; 250.*; 357.2; 362.01-362.06; 366.41<br><br>ICD-10-CM Dx: E08.*; E09.*; E10.*; E11.*; E13.*
Coronary artery/heart disease[1]	≥1 code, inpatient or outpatient claims	ICD-9-CM Dx: 410.*; 411.*; 412; 413.x; 414.*; 429.2; V45.81<br><br>ICD-10-CM Dx: I20.*; I21.*; I22.*; I24.*; I25.*; Z95.1
Hypercholesterolemia	≥1 code, inpatient or outpatient claims	ICD-9-CM Dx: 398.91; 402.x1; 404.x1; 404.x3; 428.*<br><br>ICD-10-CM Dx: I09.81; I11.0; I13.0; I13.2; I50.*
Percutaneous coronary intervention[2]	≥1 occurrence on an inpatient or outpatient claim in the CARRIER file	CPT/HCPCS: 92920; 92924; 92928; 92933; 92937; 92941; 92943
Atherosclerosis or peripheral vascular disease	≥2 codes on inpatient or outpatient claims	ICD-9-CM Dx: 411.*; 412; 413.1, 413.9; 414.0x; 414.1x; 414.2-414.9; 433.x0; 435.*; 437.0; 440.*; 441.*; 785.9; V12.54<br><br>ICD-10-CM Dx: I24.1; I20.0; I24.0; I24.1; I20.0; I24.0; I24.8; I25.2; I20.1; I20.8; I20.9; I25.10; I25.10; I25.41; I25.42; I25.3; I25.82; I25.83; I25.84; I25.5; I25.89; I25.9; I25.9; I65.1; I65.29; I65.09; I65.8; I65.8; I65.9; G45.0; G45.0; G45.8; G45.0; G45.1; G45.8; G45.9; I67.848; I67.2; I70.0; I70.1; I70.209; I70.219; I70.229; I70.25; I70.269; I70.90; I70.91; I71.00; I71.01; I71.02; I71.03; I71.2; I71.4; I71.6; I71.9; R09.89; Z86.73
Chronic obstructive pulmonary disease	≥1 code on inpatient or outpatient claims	ICD-9-CM Dx: 490; 491.*; 492.*; 494.*; 496<br><br>ICD-10-CM Dx: J40; J41.*; J42; J43.*; J44.*; J47.*
Obesity[3]	≥1 code on inpatient or outpatient claims	ICD-9-CM Dx: V85.3x; 278.0x; V85.4x<br><br>ICD-10-CM Dx: E66.0x; E66.2; E66.9; Z68.3x; Z68.4x
Angiotensin II receptor blockers	≥1 prescription fill	Drug names: Edarbi; Edarbyclor; Atacand; Atacand HCT; candesartan-hydrochlorothiazid; candesartan; Teveten; Teveten HCT; eprosartan; Avapro; Avalide; irbesartan; irbesartan-hydrochlorothiazide; Hyzaar; Cozaar; losartan; losartan-hydrochlorothiazide; olmesartan-amlodipin-hcthiazid; amlodipine-olmesartan; olmesartan; olmesartan-hydrochlorothiazide; Benicar; Benicar HCT; Azor; Tribenzor; telmisartan; telmisartan-hydrochlorothiazid; telmisartan-amlodipine; Micardis; Micardis HCT; Twynsta; Byvalson; Diovan HCT; Diovan; Exforge; Exforge HCT; Valturna; Entresto; amlodipine-valsartan-hcthiazid; valsartan; amlodipine-valsartan; valsartan-hydrochlorothiazide

© 2023 Zhang J et al. *JAMA Network Open.*

Calcium channel blockers	≥1 prescription fill	Drug names: Lotrel; Lexxel; amlodipine; Norvasc; Caduet; Exforge; Exforge HCT; Tekamlo; Amturnide; olmesartan-amlodipin-hcthiazid; amlodipine-olmesartan; amlodipine-valsartan-hcthiazid; amlodipine-benazepril; amlodipine-valsartan; telmisartan-amlodipine; amlodipine-atorvastatin; amlodipine besylate (bulk); Twynsta; Katerzia; Azor; Prestalia; Tribenzor; Consensi; Vascor; Cleviprex; diltiazem HCl; Cardizem LA; Dilacor XR; Tiamate; Cardizem; Cardizem SR; Cardizem CD; Tiazac; diltiazem HCl (bulk); Cartia XT; Diltia XT; diltiazem in dextrose 5 %; Matzim LA; DILT-XR; DILT-CD; Diltzac ER; Taztia XT; diltiazem HCl in 0.9% NaCl; Tiadylt ER; Plendil; felodipine; DynaCirc; DynaCirc CR; isradipine; Posicor; Cardene SR; Cardene; Cardene IV; nicardipine; nicardipine in NaCl (iso-os); Cardene IV in sodium chloride; Cardene IV in dextrose; nicardipine in 0.9 % sod chlor; nicardipine in 5 % dextrose; Adalat; Adalat CC; nifedipine; Procardia; Procardia XL; Nifedical XL; Nifediac CC; nifedipine, micronized (bulk); Afeditab CR; nifedipine (bulk); Nimotop; nimodipine; Nymalize; nimodipine (bulk); Sular; nisoldipine; verapamil; Verelan; Calan; Calan SR; Covera-HS; Isoptin; Isoptin SR; Tarka; Verelan PM; verapamil (bulk); trandolapril-verapamil
Beta-blockers	≥1 prescription fill	Drug names: Sectral; acebutolol; acebutolol (bulk); atenolol; Tenormin; Tenormin Calendar Pak; Tenoretic 50; Tenoretic 100; atenolol-chlorthalidone; atenolol (bulk); Senormin; Kerlone; Betoptic; Betoptic S; betaxolol; Ziac; Zebeta; bisoprolol-hydrochlorothiazide; bisoprolol fumarate; Cartrol; carteolol; Ocupress; Coreg CR; Coreg; carvedilol; carvedilol phosphate; Brevibloc; esmolol; Brevibloc in NaCl (iso-osm); esmolol in NaCl (iso-osm); esmolol in sterile water; labetalol; Normodyne; Trandate; labetalol (bulk); labetalol in dextrose 5 %; Optipranolol; metipranolol; metoprolol tartrate; Lopressor HCT; Lopressor; metoprolol succinate; Toprol XL; Dutoprol; metoprolol ta-hydrochlorothiaz; Kapspargo Sprinkle; metoprolol tartrate (bulk); Hypertensolol; metoprolol su-hydrochlorothiaz; Corgard; Corzide; nadolol; nadolol-bendroflumethiazide; nadolol (bulk); Byvalson; Bystolic; Levatol; pindolol; Visken; pindolol (bulk); propranolol; propranolol-hydrochlorothiazid; Inderal; Inderide LA; Inderal LA; Inderide; Inderide-40/25; Inderide-80/25; InnoPran XL; Betachron; Procard; Lorol; propranolol (bulk); Pronol; Inderal XL; Hemangeol; sotalol; Sotalol AF; Sorine; Sotylize; Betapace; Betapace AF; Blocadren; Timolide; Timoptic; Timoptic Ocudose (PF); Timoptic-XE; Cosopt; Cosopt (PF); Combigan; timolol maleate; dorzolamide-timolol; Istalol; timolol maleate (bulk); dorzolamide-timolol (PF); Betimol; timolol-latanoprost(PF); timolol-brimonidi-dorzolam(PF); timolol-dorzolamid-latanop(PF); timol-brimon-dorzo-latanop(PF)
Peripheral vasodilators	≥1 prescription fill	Drug names: Seratab; Ethatab; Isovex; Ethaquin; ethaverine; Cebral; Ethavex-100; Rotab; Pasmol; Rothan-100; buflomedil (bulk); nylidrin (bulk); cyclandelate (bulk); Trigot; ergoloid; Hydergine; Hydergine LC; Deapril-St; Circanol; Trigine; Niloric; Hybalergine; Tri-Ergone; Gerimal; Spengine; Hydrogin; Uni-Gine; Hydroloid-G; ergoloid (bulk); Alkergot; Trihydrogen; Codamine; H.E.A.; Ergotrate; Wigraine; Wigrettes Sublingual; Wigraine-PB; Ergostat; Cafergot; Cafergot PB; D.H.E. 45; D.H.E.45; Migranal; Regergot PB; Migergot PB; ergotamine-caffeine; dihydroergotamine; Migracet-PB; Ergotamine Tartrate W/Caffeine; Gotamine; Cafermine PB; Bel-Phen-Ergot; Bel-Phen-Ergot S; Ergocaff-PB; ergot-pentobarb-bella-caf; Cafermine; Micomp P.B.; Cafetrate; Cafetrate PB; Phenobarbital/Ergotamine/Bell; Lanatrate; Ergo-Caff-Pentobarbital; Ergo-Caff Pb; Cafagen PB; Ergomar; Ergo-Comp-Pb; Migergot; Bio-Got PB; ergotamine-phenobarb-belladon; Ercaf; Micomp-PB; Cafatine PB; Cafatine; Caffeine-Ergotamine-Pentobarb; ergotamine tartrate (bulk); dihydroergotamine mesylt(bulk); Caffer-Tab; Dhe; Belcomp-PB; Micomp PB; Ercatab; Micomp P-B; Bel/Phen/Ergot SR; Phenobarbital/Ergota/Bell/Caff; Urkaf-PB; E-Caff-PB; isoxsuprine; Vasodilan; Vasorex; Vasodigen; Icn-Isox; Vasoprine; Voxsuprine; Kendilan 10; Kendilan 20; isoxsuprine HCl (bulk); Vaso-Aca-10; Vaso-Aca-20; Valan; moxisylyte (bulk); chromium polynicotinate (bulk); Inositol Hexanicotinate; niacin; Advicor; Niaspan Extended-Release; Nicobid; Nicolar; Nico-400; Nb3; Nicocap; Tega-Span; Tega Span-250; Tega Span-400; Tega Span-125; Niacor B3; Slo-Niacin; Niacor; Nia-Bid; Nicotinex; Nico-Span; Niacels; Niac; Nicotym; Niacin Flush Free; Niacin-Time; Na-500; niacin (inositol niacinate); Endur-Acin; Endur-Thine; niacin (bulk); Nicotinic Acid; B-3-50; B3-500-GR; Niaplus; Niatab; Niacinol; Niacin No Flush; Ridicin; Niaspan Starter Pack; NiaDelay; B-3 Niacin; Trental; pentoxifylline; Pentoxil; pentoxifylline (bulk); PentoPAK; Dibenzyline; phenoxybenzamine; phenoxybenzamine (bulk); Regitine; phentolamine; phentolamine mesylate (bulk); Priscoline; tolazoline (bulk); Ginkgo Vin

© 2023 Zhang J et al. *JAMA Network Open.*

Thiazide diuretics	≥1 prescription fill	Drug names: Naturetin; Naturetin-5; bendroflumethiazide-rauwolfia; Rauwolfia/Bendroflumethiazide; bendroflumethiazide (bulk); methyldopa-hydrochlorothiazide; chlorothiazide; Diuril; Diuril IV; Sk-Chlorothiazide; losartan-hydrochlorothiazide; irbesartan-hydrochlorothiazide; captopril-hydrochlorothiazide; lisinopril-hydrochlorothiazide; enalapril-hydrochlorothiazide; moexipril-hydrochlorothiazide; olmesartan-hydrochlorothiazide; reserpine-chlorothiazide; reserpine-hydrochlorothiazide; Dithirex; benazepril-hydrochlorothiazide; Diurigen; methyldopa-chlorothiazide; fosinopril-hydrochlorothiazide; quinapril-hydrochlorothiazide; valsartan-hydrochlorothiazide; chlorothiazide sodium; Chlorulan; Hydrochlorothiazide Plus; Kenuril 500; Kenuril 250; L-Dopres-Hydrochlorothiazide; triamterene-hydrochlorothiazid; hydrochlorothiazide; amiloride-hydrochlorothiazide; spironolacton-hydrochlorothiaz; Maxzide; Maxzide-25mg; HydroDiuril; Moduretic; Sk-Hydrochlorothiazide; Dyazide; Aldactazide; Oretic; Tekturna HCT; Exforge HCT; Amturnide; Esidrix; olmesartan-amlodipin-hcthiazid; amlodipine-valsartan-hcthiazid; Tri-Zide; Aldarex Plus; Hydrorex; Hydromal; Icn-Hythide; Hydro-Z-50; Hydro-Chlor; Hydrozide-50; Spironazide; Tenzide; Hydrochlorulan; Aqua-Cen; Hydoril; Altexide; Spirozide; Hyazide; Hydro-D; Hydro-T; Decozide; J-Zide; Loqua-50; Loqua-25; Spirochlor; Diurazide; Kenazide H-50; Kenazide E; Kenazide H-25; M-Zide; Ezide; hydrochlorothiazide (bulk); Diaqua; Aquazide; Aquamet; Carozide; Hydroaca; Hyclar 50; Hyclar 25; Hychlor; Hydrozide; Microzide; Aquazide-H; Hydrotone; Zide-50; Hydroben; Triamtazide; Trimax; Tribenzor; Saluron; Diucardin; reserpine-hydroflumethiazide; Sonazide; hydroflumethiazide; methyclothiazide; Aquatensen; Enduron; Methyclothiazide W/Deserpidine; methyclothiazide (bulk); Renese; Corzide; propranolol-hydrochlorothiazid; Ziac; Timolide; Lopressor HCT; Inderide LA; Inderide; Inderide-40/25; Inderide-80/25; bisoprolol-hydrochlorothiazide; nadolol-bendroflumethiazide; Dutoprol; metoprolol ta-hydrochlorothiaz; metoprolol su-hydrochlorothiaz; Metahydrin; trichlormethiazide; Naqua; Marazide Ii; Mono-Press; Trichlorex; Spenzide; Diurese; Trichlormas; trichlormethiazide (bulk); Met-4; Re-Aqua; Ro-Methiazide
ACE inhibitors	≥1 prescription fill	Drug names: Lotrel; Lexxel; Lotensin; Lotensin HCT; benazepril; amlodipine-benazepril; benazepril-hydrochlorothiazide; benazepril HCl (bulk); Capozide; Capoten; captopril; captopril-hydrochlorothiazide; captopril (bulk); Vasotec; Vaseretic; enalaprilat; enalapril maleate; enalapril-hydrochlorothiazide; enalapril maleate (bulk); Epaned; Monopril; Monopril HCT; fosinopril; fosinopril-hydrochlorothiazide; Prinivil; Prinzide; Zestril; Zestoretic; lisinopril-hydrochlorothiazide; lisinopril; lisinopril (bulk); Qbrelis; Lytensopril; Lytensopril-90; Univasc; Uniretic; moexipril; moexipril-hydrochlorothiazide; Aceon; perindopril erbumine; Prestalia; Accuretic; Accupril; quinapril; quinapril-hydrochlorothiazide; Quinaretic; Altace; ramipril; Mavik; Tarka; trandolapril; trandolapril-verapamil

© 2023 Zhang J et al. *JAMA Network Open.*

Combination diuretics	≥1 prescription fill	Drug names: apozide; methyldopa-hydrochlorothiazide; Rauzide; hydralazine-reserpin-hcthiazid; Hydropres-25; Hydropres-50; Prinzide; Vaseretic; Diupres-250; Diupres-500; Aldoril-15; Aldoril-25; Aldoclor; Aldoril D30; Hyzaar; Aldoril D50; Salutensin; Salutensin-Demi; Avalide; Unipres; Diutensen-R; Tenoretic 50; Tenoretic 100; Zestoretic; hydralazine-hydrochlorothiazid; clonidine-chlorthalidone; losartan-hydrochlorothiazide; irbesartan-hydrochlorothiazide; telmisartan-hydrochlorothiazid; Hydroplus; Minizide; Renese-R; Accuretic; Teveten HCT; Enduronyl; Enduronyl Forte; Oreticyl; Oreticyl 25; Oreticyl Forte; Oreticyl 50; Diovan HCT; Lotensin HCT; Esimil; Ser-Ap-Es; Apresoline-Esidrix; Apresazide; Monopril HCT; Uniretic; captopril-hydrochlorothiazide; lisinopril-hydrochlorothiazide; enalapril-hydrochlorothiazide; moexipril-hydrochlorothiazide; olmesartan-hydrochlorothiazide; Hydroserpine; reserpine-chlorothiazide; Methyclothiazide W/Deserpidine; reserpine-hydroflumethiazide; reserpine-hydrochlorothiazide; Hydropine; Sae; Cam-Ap-Es; Hydro-Reserp; Salazide; Salazide Demi; H-R 50; H-R 25; HHR; Mallopress; Serpex; benazepril-hydrochlorothiazide; Diurigen W/Reserpine; Hydralazide; methyldopa-chlorothiazide; atenolol-chlorthalidone; fosinopril-hydrochlorothiazide; Atacand HCT; Sterapres; Hydrotensin-50; Hydro-Reserpine-25; Hydro-Reserpine-50; Seragen; Apresodrex; Hydroserp; Hydroserpazine; MCTZ with Deserpidine Forte; Flumezide; Hydrap-Es; Cherapas; Chloroserpine-500; Chloroserpine-250; Hydro-Fluserpine #1; Hydro-Fluserpine #2; Methy-Deserpidine Forte; Clorpres; quinapril-hydrochlorothiazide; candesartan-hydrochlorothiazid; valsartan-hydrochlorothiazide; Diu-Scrip R; Aquapres-R; Panpres; Seralazide; Hydroserpalan; Apresodex; Chloroserpine; Hydropine Hp; Hydralazine Plus 50/50; Methyclodine; Methyclodine Forte; Methydine; bendroflumethiazide-rauwolfia; Tri-Hydroserpine; Aquaserp 25; Aquaserp 50; Thiaserp 250; Serapine; Hydroprin; Hydrazide; Combipres-1; Combipres; Combipres-2; Combipres-3; Micardis HCT; Q-Pres; Minizide 2; Hydrochlorothiazide Plus; Chloroserp; Uni-Serp; Rauwolfia/Bendroflumethiazide; Marpres; Hy-Es; Hy-Zide; Chloro-Res; Serathide; Nu-Serp-D; Diuretic-Ap-Es; Diaserp W/Reserpine; Hydrosine; Serpazide; Rondameth; D-Pres; Chlorserp-250; Hrh; Benicar HCT; 3H W/R; L-Dopres-Hydrochlorothiazide; Meserpidine; Meserpidine Plus; Hydra-Zide; Genutensin; Hyserp; Serge; Hydroap-Es; Bart-Serp 25; Bart-Serp 50; Quinaretic; Hydroplus-50; Hydroserpine Plus; Diupress-500; Ser-A-Gen; Edarbyclor
Potassium sparing diuretic	≥1 prescription fill	Drug names: spironolacton-hydrochlorothiaz; Maxzide; Maxzide-25mg; Moduretic; Dyazide; Aldactazide; Aldarex Plus; Spironazide; Altexide; Spirozide; Decozide; Spirochlor; Aquazide; Hydrotone; Triamtazide; Trimax; amiloride-hydrochlorothiazide; Midamor; amiloride; amiloride HCl (bulk); Inspra; eplerenone; spironolactone; Aldactone; spironolactone (bulk); Altex; Spirazone; CaroSpir; spironolactone micro (bulk); dapsone-spironolactone-niacin; spironolactone-niacinamide; triamterene-hydrochlorothiazid; Dyrenium; triamterene (bulk); triamterene
Loop diuretics	≥1 prescription fill	Drug names: Bumex; bumetanide; bumetanide (bulk); Edecrin; Sodium Edecrin; ethacrynic acid; ethacrynate sodium; ethacrynic acid (bulk); furosemide; Lasix; Lo-Aqua; Lasaject-2; Lasaject-10; Lasimide; Fumide; furosemide in 0.9 % NaCl; furosemide (bulk); Diaqua-2; Rose-40; Furoben 40; Delone; Detue; furosemide in dextrose 5 %; Demadex; torsemide
Other diuretics	≥1 prescription fill	Drug names: chlorthalidone; Hygroton; Thalitone; Biogroton; Hylidone; Hydone; indapamide; Lozol; indapamide (bulk); Zaroxolyn; Mykrox; Diulo; metolazone

© 2023 Zhang J et al. *JAMA Network Open.*

Chronic kidney disease	≥1 code on inpatient or outpatient claims	ICD-9-CM: 60.0; 160.x; 954; 189.0; 189.9; 223.0; 236.91; 249.40; 249.41; 250.40; 250.41; 250.42; 250.43; 271.4; 274.10; 283.11; 403.01; 403.11; 403.91; 404.02; 404.03; 404.12; 404.13; 404.92; 404.93; 440.1; 442.1; 572.4; 580.0; 580.4; 580.81; 580.89; 580.9; 581.0; 581.1; 581.2; 581.3; 581.81; 581.89; 581.9; 582.0; 582.1; 582.2; 582.4; 582.81; 582.89; 582.9; 583.0; 583.1; 583.2; 583.4; 583.6; 583.7; 583.81; 583.89; 583.9; 584.5; 584.6; 584.7; 584.8; 584.9; 585.1; 585.2; 585.3; 585.4; 585.5; 585.6; 585.9; 586.; 587.; 588.0; 588.1; 588.81; 588.89; 588.9; 591.; 753.12; 753.13; 753.14; 753.15; 753.16; 753.17; 753.19; 753.20; 753.21; 753.22; 753.23; 753.29; 794.4; 160.; 249.4; 250.4; 581.8; 582.8; 583.8; 584; 585<br><br>ICD-10-CM: R94.4; N00.x; N14.0; I72.2; I70.1; N26.1; N15.0; D30.02; D30.01; D30.00; Q62.32; Q62.0; Q62.2; Q61.02; Q62.10; Q62.11; Q62.12; Q62.31; Q61.11; E08.21; E08.65; E08.22; E08.29; N25.9; E09.21; E09.22; E09.29; N18.6; M32.14; N08.; M10.3*; D59.3; K76.7; N13.1; N13.2; I12.0; I13.0; I13.2; I13.10; I13.11; I12.9; C64.2; C64.1; C64.9; C68.9; Q61.5; D41.02; D41.12; D41.22; D41.01; D41.11; D41.21; D41.00; D41.10; D41.20; N25.1; E13.22; E13.29; N25.89; Q61.8; N13.39; Q62.39; Q61.19; E13.21; E74.8; N15.8; C64.; D30.0; D41.0; D41.1; D41.2; E08.2; E09.2; E10.2; E11.2; E13.2; I12.; I13.; I13.1; M10.3; M10.31; M10.32; M10.33; M10.34; M10.35; M10.36; M10.37; N00; N01.*; N02.*; N03.*; N04.*; N05.*; N06.*; N07.*; N13.3; N14.*; N17.*; N18.*; N25.; N25.8; Q61.1; Q62.1; Q62.3; B52.0; Q61.2; Q61.3; Q61.4; N25.0; N26.9; N15.9; N16.; N25.81; M35.04; A52.75; N14.4; A18.11; M32.15; E10.22; E10.29; E10.21; E10.65; E11.22; E11.29; E11.21; E11.65; N13.30; N19.
Tobacco use[4]	≥1 code on inpatient or outpatient claims	ICD-9-CM Dx: 305.1x; 649.0x; 989.84<br><br>ICD-10-CM Dx: V15.82; F17.*; F18.209; O99.333x; Z71.6; Z72.0; Z87.891<br><br>CPT/HCPCS: 99406; 99407; G0436; G0437; G9016; S9453; S4995; G9276; G9458; 1034F; 4004F; 4001F<br><br>Drug names: Prostep; Nicotrol NS; Nicotrol; nicotine; Habitrol; Thrive Nicotine; Nicorette; Nicorette DS; Nicoderm; nicotine (polacrilex); Nicoderm CQ; Nicorette Refill; Commit; Nicotinex; Nicorette Starter Kit; Nicorelief; Stop Smoking Aid; nicotine polacrilex (bulk); NTS Step 1; NTS Step 3; NTS Step 2; Nicotine Tartrate; Quit 2; Quit 4; Chantix; Chantix Continuing Month Box; Chantix Continuing Month Pak; Chantix Starting Month Box; Chantix Starting Month Pak
Anticoagulants	0, 1, or ≥2 prescription fills	Drug names: Eliquis; Eliquis DVT-PE Treat 30D Start; Pradaxa; Savaysa; heparin (porcine); heparin lock flush (porcine); Heparin Flush; heparin (porcine) in 0.9% NaCl; heparin flush(porcine)-0.9NaCl; heparin (bovine); heparin lock flush; Hep-Pak CVC; Hep-Pak; Hep-Pak 2; Liquaemin Sodium; heparin, porcine (PF); Heparin Lock; heparin (porcine) in 5 % dex; heparin (porcine) in NaCl (PF); heparin(porcine) in 0.45% NaCl; Hep-Lock; Hep Flush-10 (PF); Hep-Lock U/P PF; Hep-Lock Flush; Hep-Lock U/P; Vasceze; Heparin LockFlush(Porcine)(PF); Monoject Prefill Advanced (PF); Monoject Prefill (PF); heparin (porcine) in D5W (PF); Lok-Pak-N; Lok-Pak-N Heparin Flush; heparin (porc)-0.45% NaCl (PF); heparin (porcine) (bulk); Heparin Combination; Lok-Pak Heparin Flush; AA 3.5% no.2 ped-D10W-heparin; Xarelto; Athrombin-K; Coumadin; Panwarfin; Sofarin; warfarin; Coufarin; Jantoven; warfarin (bulk)
Dipeptidyl peptidase IV inhibitors	≥1 prescription fill	Drug names: alogliptin; alogliptin-metformin; alogliptin-pioglitazone; Oseni; Nesina; Kazano; Tradjenta; Jentadueto; Glyxambi; Jentadueto XR; Onglyza; Kombiglyze XR; Qtern; Janumet XR; Januvia; Juvisync; Janumet; Steglujan
Glucagon-like peptide-1 receptor agonists	≥1 prescription fill	Drug names: Tanzeum; Trulicity; Byetta; Bydureon; Bydureon BCise; Xultophy 100/3.6; Victoza 2-Pak; Victoza 3-Pak; Adlyxin; Soliqua 100/33; Ozempic; Rybelsus

© 2023 Zhang J et al. *JAMA Network Open.*

Long-acting insulin	0, 1, or ≥2 prescription fills	Drug names: Humulin N NPH U-100 Insulin; Humulin N Pen; Humulin N NPH Insulin KwikPen; Insulatard Human N; Novolin N (Semi-Synthetic); Novolin N NPH U-100 Insulin; Novolin N InnoLet; Novolin N Flexpen; Novolin N PenFill; Iletin II Protamine,Zinc(Beef); insulin isophane beef pure; insulin isophane beef; insulin protamine zinc beef; Iletin II NPH (Pork); Insulatard N; insulin isophane pork pure; Insulin L Pork Purified; Insulin R Pork Purified; Iletin I Protamine,Zinc; Iletin I NPH; Humulin 70/30 U-100 Insulin; Humulin 70/30 Insulin Pen; Humulin 70/30 U-100 KwikPen; Humulin 50/50; Mixtard Human 70-30; Novolin 70/30 U-100 Insulin; Novolin 70/30 (Semi-synthetic); Novolin 70/30 InnoLet Insulin; Novolin 70-30 FlexPen U-100; Novolin 70/30 PenFill; Mixtard 70-30; Humalog Mix 75-25(U-100)Insuln; Humalog Mix 50-50 Insuln U-100; insulin lispro protamin-lispro; Humalog Mix 75-25 KwikPen; Humalog Mix 50-50 KwikPen; Novolog Mix 70-30 U-100 Insuln; Novolog Mix 70-30FlexPen U-100; insulin asp prt-insulin aspart; Humulin L; Humulin U Insulin; Monotard Human Lente; Novolin L (Semi-Synthetic); Novolin L; Iletin II Lente(Beef); insulin zinc beef purified; insulin zinc beef; Insulin Lente Beef; Iletin II Lente (Pork); insulin zinc pork purified; Basaglar KwikPen U-100 Insulin; Toujeo SoloStar U-300 Insulin; Toujeo Max U-300 SoloStar; Lantus Solostar U-100 Insulin; Lantus U-100 Insulin; Levemir U-100 Insulin; Levemir FlexTouch U-100 Insuln; Levemir Flexpen; Tresiba FlexTouch U-200; Tresiba FlexTouch U-100; Tresiba U-100 Insulin; Iletin I Lente; Iletin I Semilente; Iletin I Ultralente; Lentard Insulin Purified; Soliqua 100/33; Xultophy 100/3.6
Short-acting insulin	0, 1, or ≥2 prescription fills	Drug names: Humulin R Regular U-100 Insuln; Humulin BR; Humulin R U-500 (Conc) Insulin; Humulin R U-500 (Conc) Kwikpen; Velosulin Human R; Novolin R Regular U-100 Insuln; Novolin R Penfill (semi-synth); Velosulin BR (rDNA); Velosulin BR; Novolin R InnoLet; Novolin R Flexpen; Novolin R PenFill; Myxredlin; Velosulin Human Br; Velosulin Human; Iletin II Regular(Beef); Iletin II Regular (Pork); Iletin II Regular(Pork)Conc; Velosulin R; Insulin R Purified Pork; Purified Pork Insulin; insulin regular pork; Insulin Purified Regular(Pork); Insulin Regular Purified Pork; Humalog U-100 Insulin; Humalog KwikPen Insulin; Humalog Junior KwikPen U-100; insulin lispro; Humalog Pen; Admelog U-100 Insulin lispro; Admelog SoloStar U-100 Insulin; Fiasp U-100 Insulin; Fiasp FlexTouch U-100 Insulin; Fiasp Penfill U-100 Insulin; Novolog PenFill U-100 Insulin; Novolog Flexpen U-100 Insulin; Novolog U-100 Insulin aspart; insulin aspart U-100; Apidra U-100 Insulin; Apidra SoloStar U-100 Insulin; Iletin I Regular
Sodium-glucose cotransporter-2 inhibitors	≥1 prescription fill	Drug names: Invokana; Invokamet; Invokamet XR; Farxiga; Xigduo XR; Qtern; Jardiance; Synjardy; Glyxambi; Synjardy XR; Steglatro; Steglujan; Segluromet
Sulfonylureas	≥1 prescription fill	Drug names: Dymelor; acetohexamide; chlorpropamide; Diabinese; Glucamide; Dibatrol; Insulase; Chlorabetic 250; Micronase; Glynase; Diabeta; glyburide micronized; glyburide; glyburide (bulk); Glycron; Avandaryl; Amaryl; glimepiride; pioglitazone-glimepiride; DUETACT; glipizide; Glucotrol XL; Glucotrol; glipizide (bulk); Glucovance; Metaglip; glyburide-metformin; glipizide-metformin; tolazamide; Tolinase; Ronase; Tolamide; tolbutamide; Orinase; Orinase Diagnostic; Icn-Tolam; Oribetic
Thiazolidinedione	≥1 prescription fill	Drug names: pioglitazone; pioglitazone-metformin; pioglitazone-glimepiride; Actos; Actoplus MET; alogliptin-pioglitazone; Oseni; DUETACT; Actoplus Met XR; Avandaryl; Avandamet; Avandia; Rezulin
Biguanides	≥1 prescription fill	Drug names: Kombiglyze XR; Janumet XR; Janumet; Segluromet; Avandamet; Glucophage; Glucophage XR; Glucovance; Metaglip; metformin; pioglitazone-metformin; glyburide-metformin; glipizide-metformin; Prandimet; Xigduo XR; Jentadueto; Synjardy; Jentadueto XR; Synjardy XR; Riomet ER; Riomet; Glumetza; Actoplus MET; metformin (bulk); alogliptin-metformin; Invokamet; Invokamet XR; Fortamet; Actoplus Met XR; Kazano; repaglinide-metformin; Appformin; Appformin-D; DM2

© 2023 Zhang J et al. *JAMA Network Open.*

Opioids	≥1 prescription fill	Drug names: Levo-Dromoran; levorphanol tartrate; levorphanol tartrate (bulk); buprenorphine HCl; buprenorphine-naloxone; buprenorphine; Buprenex; Sublocade; Suboxone; Subutex; Butrans; buprenorphine HCl (bulk); Probuphine; Zubsolv; Bunavail; Belbuca; Stadol; butorphanol tartrate; Stadol NS; butorphanol tartrate (bulk); codeine sulfate; Codeine Phosphate Soluble; codeine phosphate; acetaminophen-codeine; Promethazine VC-Codeine; promethazine-codeine; Brompheniramine DC-Codeine; Sk-APAP-Codeine; Phenergan-Codeine; Phenergan VC-Codeine; codeine phosphate (bulk); Fiorinal-Codeine #3; Panadol-Codeine No.3; Panadol-Codeine No.4; Phenaphen W/Codeine; Phenaphen/Codeine #3; Phenaphen/Codeine #4; Soma Compound with Codeine; Tylenol-Codeine Elixir; Tylenol-Codeine Solution; Tylenol-Codeine #2; Tylenol-Codeine #3; Tylenol-Codeine #4; Anacin-3 with Codeine (acetam); Anacin-3-Codeine (acetaminoph); codeine-butalbital-ASA-caff; aspirin-codeine; Codaphen; Proval No.3; Fioricet with Codeine; Actifed With Codeine; Empirin W/Codeine; Empracet-Codeine No.3; Empracet-Codeine No.4; Capital with Codeine; ABC Compound with Codeine #3; butalbital-acetaminop-caf-cod; Butalbital Compound-Codeine; Mallergan-VC with Codeine; Copan; Aspirin-Codeine #3; Aspirin-Codeine #4; bromodiphenhydramine-codeine; carisoprodol-aspirin-codeine; Phrenilin w/Caffeine-Codeine; Steraphen W/Codeine; Cophene-S (dihydrocodeine); Decongestant-DH with Codeine; Isobutal with codeine; Triafed With Codeine; chlorphen-pseudoephed-codeine; codeine-brompheniramine-PPA; pseudoephedrine-codeine; Aceta-Codeine; Butalbital Compound W/Codeine; Esgic with Codeine; promethazine-phenyleph-codeine; Coastaldyne; Allerfrim with Codeine; Bromotuss-Codeine; Phenhist DH with Codeine; Isollyl W/Codeine; Isolin W/Codeine; Fiortal/Codeine; Atridine with Codeine; Idenal W/Codeine; Fiormor W/Codeine #3; Triposed with Codeine; Ty-Pap W/Codeine; Ty-Tab-Codeine; Farbital W/Codeine #3; Emcodeine No.4; Emcodeine No.3; Rid-A-Pain W/Codeine; Tricodene with Codeine; Tri-Act with Codeine; Amaphen with Codeine No.3; Codeine Antitussive Cough; Mygranol W/Codeine #3; M-Gesic; Endal CD (cpm-p.ephrn-codeine); Tylagesic 3; Codaca No.3; Codaca No.4; Vitapap-Codeine; brompheniram-PE-dihydrocodeine; Chemergan-Codeine; Chemergan VC with Codeine; Stopayne; Cocet Plus; Cocet; Ascomp with Codeine; Butinal W/Codeine #3; chlorpheniramine-codeine; Umi-APAP; Carisoprodol Compound-Codeine; An-Abate; Mi-Code; Bromphen DC-Codeine; Ascomp W/Codeine # 3; Statuss Green (codeine); Maxiflu CDX; Phenflu CDX; Cotabflu; Vopac; Darvocet-N 50; Darvon-N; Darvocet-N 100; Darvon; Darvon Compound 32; Darvon Compound-65; propoxyphene; propoxyphene N-acetaminophen; Dolene Compound-65; Dolene; Dolene Ap-65; Sk-65; Sk-65 Compound; Sk-65 Apap; Wygesic; propoxyphene-acetaminophen; Propacet 100; Propoxyphene Compound-65; Bexophene; Genagesic; Margesic (propoxyphene); Margesic Compound; propoxyphene napsylate; propoxyphene napsylate (bulk); Pancet; Doraphen-65; Lorcet (propoxyphene); E-Lor; Balacet 325; Trycet; Pro Pox 65; Dolacet; Doxapap N-100; J-Cet; Propoxacet-N 100; Dolagesic; PP-Cap; PC-Cap; Ro-Cet-N 100; Darvocet A500; Doxaphene Compound; Dalgan; Synalgos-DC; Norco; Anexsia; DHC Plus; Vicodin HP; Vicodin; Vicodin ES; hydrocodone-acetaminophen; Zydone; Hydrocet; Panasal; Panacet; Zolvit; Lortab Elixir; aspirin-caffeine-dihydrocodein; Co-Gesic; Azdone; Hycodaphen; Hy-Phen; Lortab; Dihydroxcodeine Compound; Lorcet HD; Lorcet Plus; Duradyne Dhc; Bancap HC; Anodynos-Dhc; Hydrogesic; Zamicet; Dolo-Pap; Panlor DC; Lorcide; Panlor (hydrocodone-acetamin); Sinodeine; Ceta Plus; Margesic-H; Hycet; Dihydrocodeine Comp Mod; Lorcet 10/650; Hy-5; Dolphen; Dolfen; Apadaz; benzhydrocodone-acetaminophen; Hyco-Pap; Amacodone; Propain Hc; Norcet; Norcet 7.5mg; Dolorex Forte; Damason-P; Duocet; Trezix; Xodol 10/300; Xodol 5/300; Xodol 7.5/300; Liquicet; Rogesic #3; acetaminophen-caff-dihydrocod; Megamor; Ultragesic; Ugesic; Senefen Iii; Syngesic DC; Megagesic; Hba; Lortab ASA; Lortab 5-325; Lortab 7.5-325; Lortab 10-325; Allay; Polygesic; T-Gesic Forte; T-Gesic; Adol (hydrocodone-acetaminoph); dihydrocodeine bitartrat(bulk); Anolor Dh-5; Lorcet (hydrocodone); Hycomed; Maxidone; Oncet 5; Oncet 7; Gesic-HD; Gesic-5; Hycotab; Dihydrocodeine Comp; Hycogesic; Oncet; Stagesic-10; Stagesic; Vanacet; Medicap Hd; Medipain 5; Alor; Vendone; Procet; Verdrocet; Panlor(acetam-caff-dihydrocod); Dvorah; fentanyl citrate (PF); Onsolis; fentanyl-droperidol; Fentanyl Oralet; Actiq; fentanyl citrate; fentanyl; fentanyl citrate (PF)-0.9%NaCl; fentanyl (PF)-bupivacaine-NaCl; fentanyl-ropivacaine-NaCl (PF); fentanyl citrate (bulk); Sublimaze (PF); Lazanda; Duragesic; Fentora; Subsys; fentanyl (bulk); Abstral; Innovar; Ionsys; fentanyl citrate in D5W (PF); hydrocodone bitartrate (bulk); pseudoephedrine-hydrocodone; Ru-Tuss/Hydrocodone; Ru-Tuss W/Hydrocodone; Hycomine (hydrocodone-PPA); hydrocodone-ibuprofen; hydrocodone-homatropine; phenylpropanolamin-hydrocodone; Hydrocodone Compound; pyrilam-

© 2023 Zhang J et al. *JAMA Network Open.*

		phenyleph-hydrocodone; hydrocodone-cpm-pseudoephed; hydrocodone-carbinox-pseudoep; Hydrocodone Pediatric; Rolatuss/Hydrocodone; dexbromphenirmn-PE-hydrocodone; hydrocodone-chlorpheniramine; Zohydro ER; hydrocodone bitartrate; Hydrocodone PA; Hydrocodone CP; Hysingla ER; Hydrocodone HD; Hydrocodone PA Pediatric; hydrocodone-bromphen-pseudoeph; hydromorphone; Dilaudid (PF); Dilaudid-HP (PF); Dilaudid; Dilaudid-5; hydromorphone (PF); hydromorphone-bupiv (PF)-NaCl; hydromorphone in 0.9 % NaCl; hydromorphone (bulk); hydromorphone (PF)-0.9 % NaCl; Exalgo ER; Palladone; Hydrostat; hydromorph(PF)-ropiv-0.9% NaCl; hydromorphone in D5W (PF); hydromorphone (PF) in water; Dolophine; methadone; Methadose; methadone (bulk); Methadone Intensol; Diskets; methadone in 0.9 % sod.chlorid; morphine; morphine (bulk); Kadian; MS Contin; MSir; MSir Concentrate; morphine concentrate; Roxanol Concentrate; Roxanol-T Concentrate; Roxanol SR; Oramorph SR; Roxanol; Rescudose; morphine (PF); morphine (PF) in dextrose 5 %; morphine in dextrose 5 %; Astramorph-PF; RMS; OMS Concentrate; morphine in 0.9 % sodium chlor; Duramorph (PF); Infumorph 200 P/F; Infumorph 500 P/F; Infumorph P/F; APOKYN; morphine (PF) in 0.9 % sod chl; Avinza; DepoDur (PF); Embeda; apomorphine (bulk); morphine(PF)in iso sod chlorid; MS/S; MS/L; MS/L-C; Kynmobi; MorphaBond ER; Mitigo (PF); Arymo ER; nalbuphine; Nubain; nalbuphine (bulk); paregoric; opium tincture; dextromethorphan-guaifenesin; belladonna alkaloids-opium; Cheracol D; Naldecon Senior DX; Naldecon-Cx Adult; Naldecon-DX Pediatric; Naldecon-DX; Naldecon Dx; Cough Formula Expectorant DM; Pediacof; Nucofed Pediatric Expectorant; Nucofed; Dimacol; Dimetapp Cold-Congestion; Robitussin Cold and Cough; Robitussin Cough-Cold CF Max; Robitussin Cough and Cold CF; Robitussin Cough-Congestion; Robitussin DM Max; Robitussin A-C; Robitussin-CF; Robitussin Ped (with p-ephed); Robitussin CF Infant; Robitussin DAC; Robitussin DM Infant Drops; Robitussin-DM; Robitussin-Cough-Chest-Cong; Adult Robitussin Cough-Cold D; Robitussin DM To Go; Chld Robitussin Cough-Chest DM; Robitussin Cough-Chest Cong DM; Robitussin M-S Cold CF Max; Adult Robitussin M-S Cold; Adult Robitussin Peak Cold DM; Adt Robitussin Peak Cld DM Max; Adult Robitussin Peak Cold M-S; Ryna-CX; TUSSI-ORGANIDIN DM-S NR; TUSSI-ORGANIDIN DM NR; TUSSI-ORGANIDIN-S NR; TUSSI-ORGANIDIN NR; Triaminic DH Expectorant; Triaminic With Codeine; Dorcol; Vicodin Tuss; Dilaudid Cough; Ru-Tuss Expectorant; Medi-Tuss Dac; Medi-Tuss DM; Ru-Tuss; Hycotuss Expectorant; Child Triaminic Cough-Congest; Novahistine DMX; Tussend; Novahistine; Novahistine Expectorant; 2/G-DM; Benylin Multi-Symptom; Benylin; Calcidrine; Tussar-SF; Tussar-2; Sudafed Cough; Formula D; Rome-Tuss DM; Cough Control DM; Cough Control CF; Romex 'Jr'; Isoclor; Pneumotussin HC; Pneumotussin; Tussin Cold And Cough; Decongestant D Cough; Tussin DM; Tussin DM Cough and Chest; Tussin CF (PPA-DM-guaif); Child Mucus Relief Cough; Tussin CF (PE-DM-guaif); Tussin CF; Tussin DM Max; Child's Mucus Relief M-S Cold; codeine-guaifenesin; Hydrocodone-GG Expectorant; hydrocodone-guaifenesin; Guaifenesin DM; Qual-Tussin DC; Qual-Tussin; Cough Formula-DM; Cough Formula CF; Dextro-Tuss GG; CodiCLEAR DH; Iofen-C NF; Iofen-DM NF; Vortex; Guaispan DM; Rex-A-Hist; Brontex; Guiatuss DAC; Detussin; Murtuss GG; Guiatuss DM; Codafed; Hycofenix; Flowtuss; Guiatuss AC; phenylprop-codeine-GG; pseudoephedrine-codeine-GG; Guiatuss; Cough Syrup; Tussgen; Dihistine Expectorant-Codeine; Dihistine; Genatuss DM; Guiatuss CF; Dihistine Dmx; Pediacon DX; Clear Tussin 30; Guiatuss Clear DM; Guiatuss Clear; Pediacon DX Childrens; Cophene-S; Cophene-Xp; Cophene Xp; Detussin Expectorant; Kwelcof; Iophen DM-NR; Donatussin DC; Bowtussin DM; Naldelate DX Childrens; Guaibid DM; Cheratussin AC; Cheratussin DAC; Codafed Expectorant; Codafed Pediatric Expectorant; Dritus HD; Guaifen-C; Iophen C-NR; Phenylhistine Expectorant; Quindal Expectorant; Vi-Q-Tuss; Anatuss DM; Anatuss; Anatuss Wtih Codeine; Tolu-Sed DM; Cough Suppressant-Expectorant; Myci-GC; Donatussin (with guaifenesin); Codistan No.1; Noratuss Ii; Decongestant Expectorant; Decophed; Tussex Cough; Ceritussin-DM; Hydone; Tuss-DM; Partuss DM; G-Tussin Dac; G-Tussin DM; Decongest; Paragoric; Mucus Relief DM Max; Mucus Relief Congestion-Cough; Wal-Tussin DM; Wal-Tussin Cold and Congestion; Cough-Chest Congestion DM; Children's Cough; Wal-Tussin Cough and Cold CF; Mucus Relief DM; Wal-Tussin CF; Mucus DM Max ER; Adult Wal-Tussin DM Max; Wal-phed; Child Multi-Symptom Cold/Cough; Guiacough-CF; Guiacough DM; Tussirex; Tussirex Sugar Free; Scot-Tussin DM2; Scot-Tussin Senior; Romilar AC; Glycotuss-DM; Atrovent; Guiadrine DM; Phenylhistine; DM Cough Formula; Antitussive DM; Decongestant Cough; Cough Syrup DM; Antitussive CF; Mytussin AC; Mytussin DM; Mytussin DAC; Decohistine; Anti-Tuss DM; C-Tussin;

© 2023 Zhang J et al. *JAMA Network Open.*

		Conex with Codeine; Ambenyl-D; Broncot; Hycosin Expectorant; Eff Str Cough W/Decongestant; Tussex; Naldelate Dx; Naldelate Senior Dx; Phenadex; Phenadex Children's; Phenadex Senior; Nucotuss Expectorant; Cough Formula W/Decongestant; Deconamine CX; Src; Tussex DM; Benylin Expectorant; Children's Sudafed Cold-Cough; Amquintussin DM; Pancof XP; PanMist DM; Pancof EXP; Tussafin; Guiatussin DAC; Guiatussin W/Codeine; Guaitussin with Codeine; Diabetic Dm; Mucus DM; Primatuss 4d; Pseudoephedrine G w/Codeine; Phenhist; Tri-Dec; Tri-Dec Pediatric; Tri-Dec Dx Senior; Hydrotuss; Guiatussin DM; Spen-Histine; Orahist; G-Tuss; Gua C; Gua PC; Gua HC; Tuss-AX; Phanatuss DM; Children's Formula; Tusscidin DM; Tusscidin-D; Guaifenesin DAC; Tusscidin CF; Cough Formula Expectorant; Hytussin; Guaifenesin AC; Broncopectol; Sortuss; Guiatuss-Bertuss DM; Halotussin AC; Halotussin DAC; Spiriva with HandiHaler; Atrovent Refill; Atrovent HFA; Spiriva Respimat; Stiolto Respimat; Cough Formula D; Naldelate DX Pediatric; Q-Tussin CF; Q-Tussin DM; Q-Tussin CF (PE); Drituss DM; Pro-Cof; Pro-Cof D; Quintex HC; Tussiden C; Tussiden DM; Tussiclear DH; Q-Bid DM; Guiamid DM; V-Tuss; Tusso-HC; Tussafed-HCG; Tussafed HC; Tusso-C; Tusso-DF; Tusso-XR; Tusso-DM; Tusso-DMR; Tussafed-LA; TriTuss-ER; TriTuss; Tussafed EX; QUALA-TLA; QUALA-HC; Gutatuss A/D; Gutatuss-DM; Uni-Tussin DM; Uni-Tussin CF; Uni-Formula 88-D; pseudoephedrine-DM-guaifenesin; hydrocodone-pseudoephed-guaif; hydrocodone-potassium guaiaco; Guai-DM HB-P-Ephed; Guaifen-Dextrom-Pseudoephedrin; Uni-Hist DM; phenylephrine-hydrocodone-GG; GFN600-PSE60-DM30; UNI-COF EXP; Uni-Hist PDX; Uni-Lev; Marcof Expectorant; Marcof; Mar-Cof CG; Mycotussin; Myhistine; Tussin Dac; Pediatric Cough; Cheratussin DM; Polytuss-DM; Cotuss DM; Deproist-Codeine; Glydeine; Glydm; Endal Expectorant; Lotussin DM; Nuco-Tuss; Nalphen DX; Nalphen DX Pediatric; Halotussin-DM; Phenilar Cough; Dexatuss; Halotussin DM; Halotussin-CF; Guaifenesin-DM; Cough Syrup W/Decongestant; Robafen DAC; Robafen DM; Robafen DM Cough-Chest Congest; Robafen AC; Robafen CF; Tussaphen-DM; Novagest DEX; Novagest; Novagest Expectorant/Codeine; Rolatuss; Pedituss Cough; Formula-Dm Cough Syrup; Pseudogest; Dieter's Cough Syrup; Efasin; Iophen; Robafen Cold and Cough; Robafen DM Clear; Robafen CF (phenylephrine); Robafen DM Max; Robafen DM Cough; Mucus Relief DM Cough; Robafen DM Peak Cold; Rolatuss Expectorant; Antitussive/Decongestant; Nolamine DEX Infants Drops; Nolamine DEX Childrens; Nolamine Dex; Codotuss; Dia-Tuss; Kolephrin-GG-DM; W/W-Histine; B and O 15-A Supprette; B and O 16-A Supprette; Tonecol Cough; Albatussin NN; Albatussin Pediatric; Albatussin DM; Albatussin DM Pediatric; Albatussin CF Pediatric; Albatussin CF; Albatussin SR; Humibid CS; Robelene Dm; Troutman's DM; Quiecoff; Pharm-A-Tussin w/DM; Guai 800-DM 30; DM-Guai; HC-Guai; phenylephrine-DM-guaifenesin; Chest Congestion Relief DM; Mucus Relief Cough; Cough-Head Congestion Relief; Expectorant CF; Tussin DM Clear; Tussin Cough-Cold; Pediatric Formula; Suda-Tussin DM; Recofen D; Recofen DX-Redacon DX; Redacon DX; Recofen Caps; Recofen Plus; Double-Tussin DM; Refenesen DM; Tussilan; Broncotron-D; Broncotron; Broncotron-S; Panatuss; Panatuss DX; Broncotron PED; Expectorant DM; Ultra DM Free and Clear; G-Fenesin DM; Coricidin HBP Chest Cong-Cough; Tenar DM; Tuss-DA NR; Despec-DM (pseudoeph-DM-guaif); Nectatussin Sf Y Af; Despec; Despec-DM (phenyleph-DM-guaif); Despec DM-G; Despec-EXP; Despec NR; Despec EDA Cough-Cold Drops; Mucus and Cough Relief; Cough Formula DM; Tussin DM Cough; Cough and Cold; Fast Mucus Rlf Congest-Cough; Adult Cough Formula DM Max; Dilituss; Ortac DM; Tussin Cold-Congestion; Tussin Cough-Chest Congestion; Cough and Cold Mucus Relief CF; Aquabid-DM; Wal-Tussin DM Clear; Iso; Bronkisan; Bronkisan A/C; MonteFlu HC; Monte-G HC; Baytussin DM; Bayhistine; Guaifenex DM; Baycotussend; Baytussin Dac; DPH-brdphn-codein-AmCl-Kguaiac; Super Tussin DM Clear; Super Tussin DM; Super Tussin CF; Super Tussin; Monatuss-DM Cough; Vanex; Ken Tuss DM; Ken Tuss S/F; Neo-Tuss; NeoTuss-D; NeoTuss-D (chlorpheniramine); NeoTuss-D (Improved Formula); Benylin-DME; M-CLEAR; M-Clear WC; M-CLEAR JR; Mardrops DM; Mardrops-DX; AllanHist PDX; Z-DEX Pediatric; Z-Dex; TL-Dex DM; Extendryl HC; Levall 5.0; Gentex HC; Cough Control DM Max; Tab Tussin DM; Formula D Cough Medicine; Cough Control CF (PE); Ru-Tuss DM; Ru-Tuss 800 DM; Donatussin DM; Donatuss XP; Donatuss DC; Alka-Seltzer Plus Mucus-Conges; AQUATAB DM; AQUATAB-C; Glyatuss CF; Glyatuss-DM; Glyatuss Dac; Decongestant Formula; Mucinex DM; Rydex DM; Trymine CG; Ninjacof-XG; Chlordex GP; Recofen Dx; Entuss; Creo-Terpin (DM-guaifenesin); Tidafen DM; Vicks 44 CustCare Chesty Cough; Vicks Nature Fusion Cough-Cong; Vicks Dayquil Mucus Control DM; Vicks Pediatric 44E; Vicks Formula 44E; Chest Congestion-Cough Relief; Nalphen DX

© 2023 Zhang J et al. *JAMA Network Open.*

		Childrens; Bidex-A; Bidex-DMI; Medent-DMI; Endacon-DM; Endacon; Duravent DM; 30PSE-150GFN-15DM; TG 45PSE-400GFN-15DM; TG 10PEH-380GFN-15DM; Pedicon D.M.; Aquanaz; Capmist DM; Mucus Relief ER DM-MAX; Hydro-Coff; Zhist; Intense Cough Reliever; RelCof C; Relhist DMX; Nortedril; Tussin CF MAX; Tussin CF Cough-Cold; Tussin (guaif-dm-pseudoephed); Cough Formula DM Clear; Tussin Cough DM; Severe Congestion and CoughMax; DM Max; Tussin Cough-Cold-Chest; BP 8 Cough; Entre-Cough; Entre-Cough Tannate; Ultra Tuss Safe; Mucosa DM; Zodryl DEC 25; Zodryl DEC 30; Zodryl DEC 35; Zodryl DEC 40; Zodryl DEC 50; Zodryl DEC 60; Zodryl DEC 80; Humigen DM; Entuss-D Jr; Histafed-C; Entuss Pediatric; Entuss-D; Syracol-Cf; Dexafed; G-Bronco-D; Rantussin-N; Biogil; Bionel; Bionel Pediatric; Biocotron-D; Biocotron; Biospec DMX; Biobron SF; Biodesp DM; Bio T Pres; Bio T Pres Pediatric; Bio-S-Pres Dx; BioGtuss NF; Biobron DX; Clorfed; Medent C; Bidex-DM; Medent DM; Daytime Mucus Relief DM; Liquitussin-DM; Pseudocodone; Lemotussin-DM; Acatuss DM; Acahistine; Guaificon DM; Guaicon DM; Guaicon DMS; Medikoff Sugar-Free; MediFirst Cold Relief; T-Tussin DM; T-Tussin CF; Albapec CF; Vita-Numonyl NR; Conpec DM; Broncopectol CF; Broncopectol CF Pediatric; Numonyl DX Pediatric; Numonyl DX; Tussibid; Broncopectol NN SR; Albatussin SR F; Albatussin SR Senior; Medi-Tussin DM; Intense Cough; Medi-Tussin CF; Medi-Tussin DM Diabetic; Medi-Tussin; Decongestant D; Medi-Tussin Cough and Cold; Vihistine; Tusibron DM; PE-CP-K GUAIACOL; chlorpheniramine-PE-DM-guaifen; Brontuss LA; Brontuss SF; Brontuss DX; Amibid DM; Diabetic Siltussin-DM; Tussin DM Cough Medicine; Pulmovital; Novatuss; Hydro-Tuss; Diabetic Tussin DM; Diabetic Formula Supress-Expec; Co-Histine Expectorant; Guaiatussin AC; Guaiatussin DM; Guaiasorb DM(guai-dm-pot-citr); Guiatussin CF; Guaifenesin CF; HT-Tuss DM; Guaiasorb; Tussaphen; Cough Formula 4-D; Severe Cough-Congestion; Child Chest Congestion-Cough; Child Cough-Chest Congest DM; Tussin DM Day-Night; Nuprin Cold Rel Cough, Chest; Nuprin Cold Rel Cough, Nasal; Virtussin AC; Duratuss HD; Duratuss DM; Promist; Chem-Tuss Ne; Chem-Tuss Dme; Novadyne; Hydrophed Expectorant; Novadyne Dmx; Chemdal; Nucochem Expectorant; Nuchochem Pediatric; Luchem Cough Mixture Dpg; Robichem DM; Propatuss; Robichem Ac; Robichem CF; Robichem DAC; Sudachem Cough; Deconex DM; Poly-Vent DM; Poly-Tussin XP; Lortuss EX; Poly-Tussin EX; Deconex DMX; Poly-Tussin XP Expectorant; Mastussin DM; Mastussin CF; Vita-Numonyl Pediatric; Vita-Numonyl AD; Numobid DX; Vita-Numonyl DM; S-Pak DM; MedTuss HD; MedCodin; Duradal; DuraGanidin NR; DuraGanidin DM; Codegest; Rhinosyn-DMX; Harber Hist DMX; Harbertussin CF; Harbertussin DM; Fenesin DM; Sorbutuss; Nalex Expectorant; Prolex-Dh; Prolex DH; Nortuss-DE; Nortuss-D.E.; Nortuss-Ex; Cleartuss DH; Guiadex DH; Guia-D; Coldmist DM; Histacol DM; Dynatuss DF; Spantuss HD; ColdCough XP; Dynatuss-EX; Dynatuss HC; Dynatuss HCG; Coldcough EXP; Crantex HC; Mindal DM; Hydro Pro; Hydro Pro D; Hydro Pro DM; Mintuss NX; Mintuss EX; Mintuss G; Mintab DM; Mintab C; Fentuss Expectorant; Guaifen DM; Guiaplex HC; Liquicough DM; GUIAFEN II DM; Demi-Cof; Quartuss; Pseudatex HC; Guiadrine DX; Deka; Pediatuss; Supress DX; Supress DM; Tussi-Pres; Tussi-Pres Pediatric; Tussi Pres-B (guaif-dm-bromph); Tusicof; Zyncof; G-Zyncof; G-Tron; Pecgen DMX; Tusslin; Pecgen PSE; Pres Gen; G-Tusicof; Desgen; Desgen DM; Desgen DM (pseudoephedrine); Pres Gen Pediatric; G-Supress DX; Sorbugen NR; G-Tron PED; Med-Hist; Med-Hist Expectorant; Quindal; Naldecold DX; Adult Tussin Multi-Symp Cold; Adult Tussin DM; Adult Tussin Cough Congest DM; Tussid DM; Liquitussin; No Cough Plus; Peditussl; Novadyne Expectorant; Iophen DM; Humibid DM; Deconsal C; Deconsal; Syn-Rx DM; Safe Tussin DM; Altipres Pediatric; Altipres; DM; Ri-Tussin DM; Dometuss Dm; Dometuss-DMX; Entuss (guaifenes/hydrocodone); Cheracol Cough; Endal; codeine-calcium iodide; Cough Formula; Hycotuss; phenylpropanolamine-DM-GG; Protuss; Protuss-DM; Muco-Fen 800 DM; Profen II DM; Atuss EX; Kita La Tos-DM; Dex-Con Childrens; Guaifenesin-DM NR; Siltussin DM DA; Siltussin-DM; Siltussin DM DAS; Diabetic Siltussin-DM Max Str; Siltussin CF; Tusnel New Formula; Tusnel Diabetic; Tusnel DM; Tusnel C; Tusnel Pediatric; Tusnel Ped-C; Tusnel-HC; Tusnel DM Pediatric(pseudoeph); Tusnel DM Pediatric(phenyleph); Phenhist Expectorant; GFN 1200/DM 60; EndaCof; DuraDEX; Maxifed DMX (IR); Children's Mucinex Cough; Orituss-Dx; Mucobid Dm; Bitex; Pediatric Cough Liquid; Nucochem; Hydro-Tussin HD; Tussidin Dm NR; Sudatuss-2; Sudatuss-SF; Genexpect DM; Genetuss; Genantuss; Genecof-XP; Genetuss-2; Pharmatuss DM; Jaycof; Jaycof-XP; Phanatuss HC; Medatussin; Meda Forte; Safe Tussin 30; Extussive; Tussin Ac; Dacol; Alatuss; Tussin Cough; Guaidex-TR; Clear Cough DM; Clear Cough; Geri-Tussin DM; Guaiasorb DM; G Tussin AC; Endituss in; Decotuss-HD; Vicotuss; Tussi-Phen;

		Lartus; Guaifenex-Rx DM; Pseudovent DM; Hydro-Tussin DM; Hydro-Tussin XP; Tri-Vent DM; Hydro-Tussin EXP; Hydro-Tussin HG; Trispec-DM; Trispec-SF; Trispec-PE; TRISPEC DMX; TRISPEC DMX Pediatric; TRISPEC PSE; TRISPEC PSE Pediatric; Trispec SFX; CGU WC; Codemine; MC Cough; MC JR Cough Syrup; ZTUSS; Z-Tuss E; Statuss; Statuss Expectorant; Ztuss Expectorant; Ztuss ZT; Z-Tuss 2; Z-Tuss DM; Giltuss Pediatric; Giltuss; Giltuss HC; Suttar-SF; Suttar-2; Biotuss; Giltuss Ped-C; Biotuss Pediatric; Exactuss; Giltuss Cough-Cold; Childrens Giltuss Cough-Cold; Giltuss HBP; Giltuss Diabetic; Giltuss CR; Giltuss TR; Exactuss TR; Allfen CDX; Maxifed DM; Maxiphen DMX; Allfen DM; Allfen DMA; Allfen CD; Maxifed DM (IR); Maxifed CD; Maxifed CDX; Maxifed-G CD; Maxifed-G CDX; Ambifed DM; Ambifed-G DM (IR); Ambifed-G CD; Ambifed-G CDX; Ambifed CD; Ambifed CDX; Maxiphen DM; Maxiphen CD; Maxiphen CDX; Maxifed DMX; Maxi-Tuss DM; Maxi-Tuss HCG; Endotuss Expectorant; Nucodine EXP; Nucodine PED; Bron-Tuss; Orgadin-Tuss; Orgadin-Tuss DM; Ameritussin DM; Co-Tussin; Ameritussin DM Pediatric; VanaTab DM; Vanacof DM; Carbatuss-CL; Vanacof DMX; Vanacon; Touro CC; Touro DM; Touro CC-LD; Touro HC; Tussin Cough/Cold Liquid Caps; SINUtuss DM; Sinuvent II DM; Sympak DM; Muco-Fen DM; Profen Forte DM; Altarussin DM; Altarussin CF; Seudafaire Cough; Fenasyn DM; Fenasyn Dm Plus; Fenex DM; Antitussin DM; Protuss-D; Atuss G; Atuss NX; Sudal DM; Atuss-12 DX; Atuss HX; Biotussin AC; Biotuss CF; Biotussin DAC; Biotuss-DM; Cotuss-V; Codafen; Tussidin Nr; Tussadur-HD; Alphen Expectorant; Guai-Co; Guai-Dex; Phenydex; Anextuss; DACEX-DM; DEXCON-DM; Su-Tuss DM; Relacon-DM; Dacex-PE; GFN 1200-DM 20-PE 40; GDP-EX; Gani-Tuss NR; Gani-Tuss DM NR; GFN 1000/DM 60; GFN-DM-PSE; GFN 595-PSE 48-DM 32 Tab; GFN 500-DM 30; GFN 550-PSE 60-DM 30; GFN 1000-DM 50; GFN 800-PSE 60-DM 30; GFN 800-DM 30; Extuss LA; Dexcon-PE; Dex-Tuss DM; Phlemex-PE; Phlemex; Phlemex Forte; Cofex-DM; Relacon LAX; Relasin DM; Relacon-DM NR; Simuc-DM; Bromhist-PDX; Bromhist-DM; Dex-Tuss; Cycofed Expectorant; Cycofed Pediatric Expectorant; Canges-XP; Relasin-HCX; Cyndal Expectorant; Su-Tuss HD; Narcof; Vitussin; Hydron KGS; Hydron EX; De-Chlor NX; KGS-HC; Hy-KXP; Dihydro-GP; C-Cof XP; Hydro GP; De-Chlor G; Simuc-HD; G Bid DM; Mytussin CF; Nalspan Senior DX; Hydrocodone GF; Decohistine Expectorant; Santuss; Diabetic Tussin; Respa DM; Tricode GF; Trikof-D; Codar GF; Tussedyn; KG-Tuss HD Expectorant; KG-Fed Expectorant; KG-FED Pediatric Expectorant; Cough DM; Supressin Dm; Pediapressin; Tussend Expectorant; Nucofed Expectorant; Monafed DM; Iobid DM; TUSSITAB; Vicoclear; Med-Rx DM; Diabetic Tussin DM Max Str; Diabetic Tussin C; Diabetic Tussin Max St; Kosher Care DM; Gripex DM; Gripex CF; Entex HC; Guaifenesin NR; Komatussin DM; Komatussin CF; Fenesin DM IR; Numobid CF; Actinel; Actinel Pediatric; Actidom DMX; Pulexn DM; Xpect-HC; Nariz HC; Children's Mucinex Multi-Symp; Mucinex Fast-Max Congest-Cough; Mucinex Fast-Max DM Max; Mucinex Cough; Delsym Cough-Chest Congest DM; Child Delsym Cough-Chest DM; Child Mucinex Cough Mini-Melts; Child Mucinex Congestion-Cough; Child Mucinex Cough-Congest; Child Mucinex Freefrom Day Cgh; Tussidex; Dex GG; Pseudo Max DMX; G-BID DM TR; G PHEN DM; PDM GG; Pseudo DM GG; PediaHist DM; Pseudo Cough; Tussi-bid; Certuss-D; J-MAX DHC; Cough-Ex; Cough Suppressant; Entab DM; TUSSIGEN-N.F; LANZATUSS-N.F.; Tussilan-N.F.; Tussilan-G; Zyrphen-HC; A-COF DH; Z-Cof LA; Z-Cof LAX; Aldex GS DM; Z-Cof HCX; Z-Cof 12 DM; Z-Cof 8 DM; Z-Cof DM; Z-Cof I; Z-Cof DMX; Accuhist DM; GeneBronco-D; GenePatuss; Genelan; Genedotuss-DM; Genexpect-SF; Genexpect-PE; Guapetex HC; Guaitussin AC; Guaitussin DAC; Protex; Protex D; Guaimist DM; Tridal; Tricof EXP; Cotuss EX; AccuHist PDX; Pro-Clear Caps; Pro-Clear; Dynex HD; DuraDEX Forte; DURAPHEN DM; DuraMAX; Duraphen II DM; Duraphen Forte; Ambifed-G DM; Maxiphen-G DM; AMBI 45-800-30; AMBI 5-15-100; AMBI 80-700-40; AMBI 1000-55; AMBI 60-580-30; AMBI 80-780-40; Ambi 60PSE-400GFN-20DM; Ambi 40PSE-400GFN-20DM; Ambi 10PEH-400GFN-20DM; Ambitussin AC; AMBI 60-1000-30; AMBI 40-1000-60 (PE); FluTuss XP; Entex PAC; WellTuss EXP; P-Tuss; P-Tuss D; P-Tuss DM; Guaifenesin/P-Ephedrine/Cod; Zotex; Zotex-LA; Zotex-G; Zotex-DM; Zotex LAX; Zotex DMX; Zotex-EX; Zotex-D (with DM-PE); Guaphen FORTE; Guaphen II DM; Condasin; EndaCof-XP; ExeFen-DM; ExeFen-DMX; ExeFen DMX; ExeCof; ExeCof-XP; Exetuss-HC; Exetuss-DM; DroTuss; HydroFed; ExeClear; ExeClear-C; ExeClear-DM; SudaTex-DM; Phendacof HC; Nazarin HC; Duratuss DM12; Obredon; Virtussin DAC; Coditussin AC; Coditussin DAC; Pertussin Am; Nivanex DMX; oxycodone HCl-oxycodone-ASA; oxycodone-acetaminophen; Tylox; oxycodone; Roxicodone; Roxicodone Intensol; Roxicet; Roxiprin; oxycodone-aspirin;

© 2023 Zhang J et al. *JAMA Network Open.*

		Percocet; Percodan; Percodan Demi; ibuprofen-oxycodone; oxycodone (bulk); Combunox; OxyContin; Magnacet; Endocet; Endodan; Xolox; Xartemis XR; Primlev; Xtampza ER; ETH-Oxydose; OxyIR; Oxaydo; Oxyfast; Primalev; Oxecta; Endodan (Old Formula); Endocodone; Percolone; Dazidox; RoxyBond; Perloxx; ALCET; Lynox; Narvox; Nalocet; Prolate; oxymorphone; Numorphan; Opana ER; Opana; Talwin; Talwin Compound; Talacen; Talwin NX; pentazocine-naloxone; pentazocine-acetaminophen; Mepergan; meperidine (PF); meperidine; Mepergan Fortis; Atropine-Demerol; Demerol; Demerol (PF); meperidine-promethazine; Meprozine; meperidine in 0.9 % NaCl; meperidine (bulk); meperidine (PF) in 0.9 % NaCl; Meperitab; Nucynta; Nucynta ER; Ultracet; Ultram; Ultram ER; tramadol; tramadol-acetaminophen; tramadol hydrochloride (bulk); Ryzolt; ConZip; Rybix ODT; Theratramadol-60; Theratramadol-90
Statins	≥1 prescription fill	Drug names: Simcor; Caduet; Lipitor; atorvastatin; amlodipine-atorvastatin; Liptruzet; Baycol; Lescol; Lescol XL; fluvastatin; Mevacor; Advicor; lovastatin; Altoprev; Altocor; Livalo; Zypitamag; Pravachol; PRAVIGARD PAC; pravastatin; rosuvastatin; Crestor; Ezallor Sprinkle; Juvisync; Zocor; simvastatin; ezetimibe-simvastatin; Vytorin 10-40; Vytorin 10-20; Vytorin 10-10; Vytorin 10-80; FloLipid; simvastatin (bulk)
Coronary artery bypass grafting	≥1 code on the inpatient record	CPT/HCPCS: 33510; 33511; 33512; 33513; 33514; 33516; 33517; 33518; 33519; 33521; 33522; 33523; 33533; 33534; 33535; 33536; S2205; S2206; S2207; S2208; S2209

Abbreviations: CPT=Current Procedural Terminology, HCPCS=Healthcare Common Procedure Coding System; ICD-9-CM=International Classification of Diseases, 9th Revision, Clinical Modification; ICD-10-CM: International Classification of Diseases, 10th Revision, Clinical Modification; N/A=not applicable

National Drug Codes are included in the code list posted on GitHub (https://github.com/chasedlatour/AB-v-5ARI-Medicare).

© 2023 Zhang J et al. *JAMA Network Open.*

Figure S1. Month-level prevalence of our primary outcome definition for in-patient hospitalization for heart failure over the ICD-9 to ICD-10 transition. Estimates were calculated in the entire Medicare enrollee population from 2013-2017.

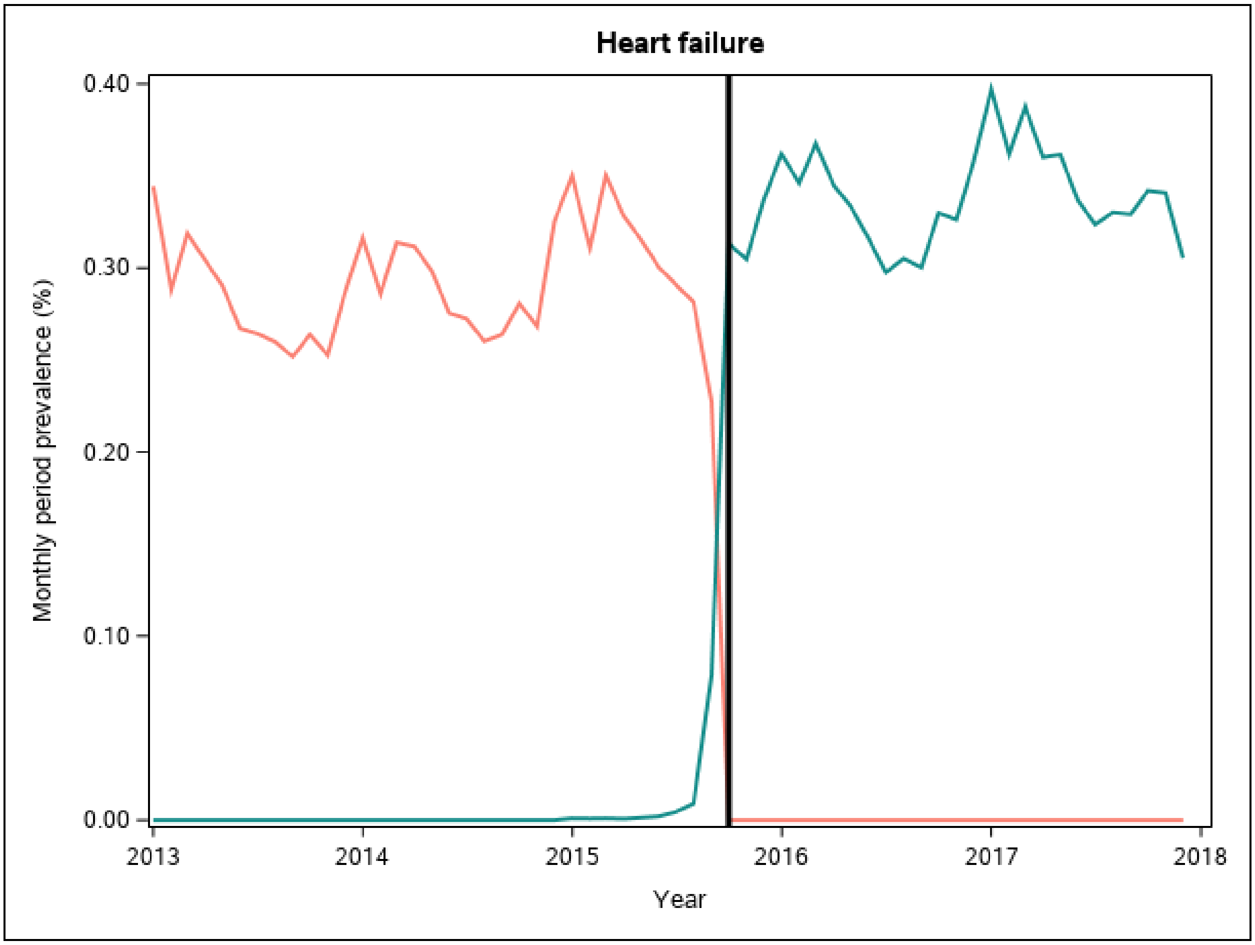

Monthly prevalence estimates were calculated across the entire 20% random sample of Medicare enrollees from 2013-2017. An individual was considered at-risk within a given month if they were enrolled in Medicare Parts A and B during that month. If an individual had ≥1 claim meeting an outcome definition within that given month, they were counted as having an outcome event during that month. Prevalence estimates were then calculated by dividing the number of individuals who experienced ≥1 qualifying event within a month by the number of individuals identified as being at-risk during that month.

Figure S2. Month-level prevalence of our primary outcome definition for in-patient hospitalization for stroke over the ICD-9 to ICD-10 transition. Estimates were calculated in the entire Medicare enrollee population from 2013-2017.

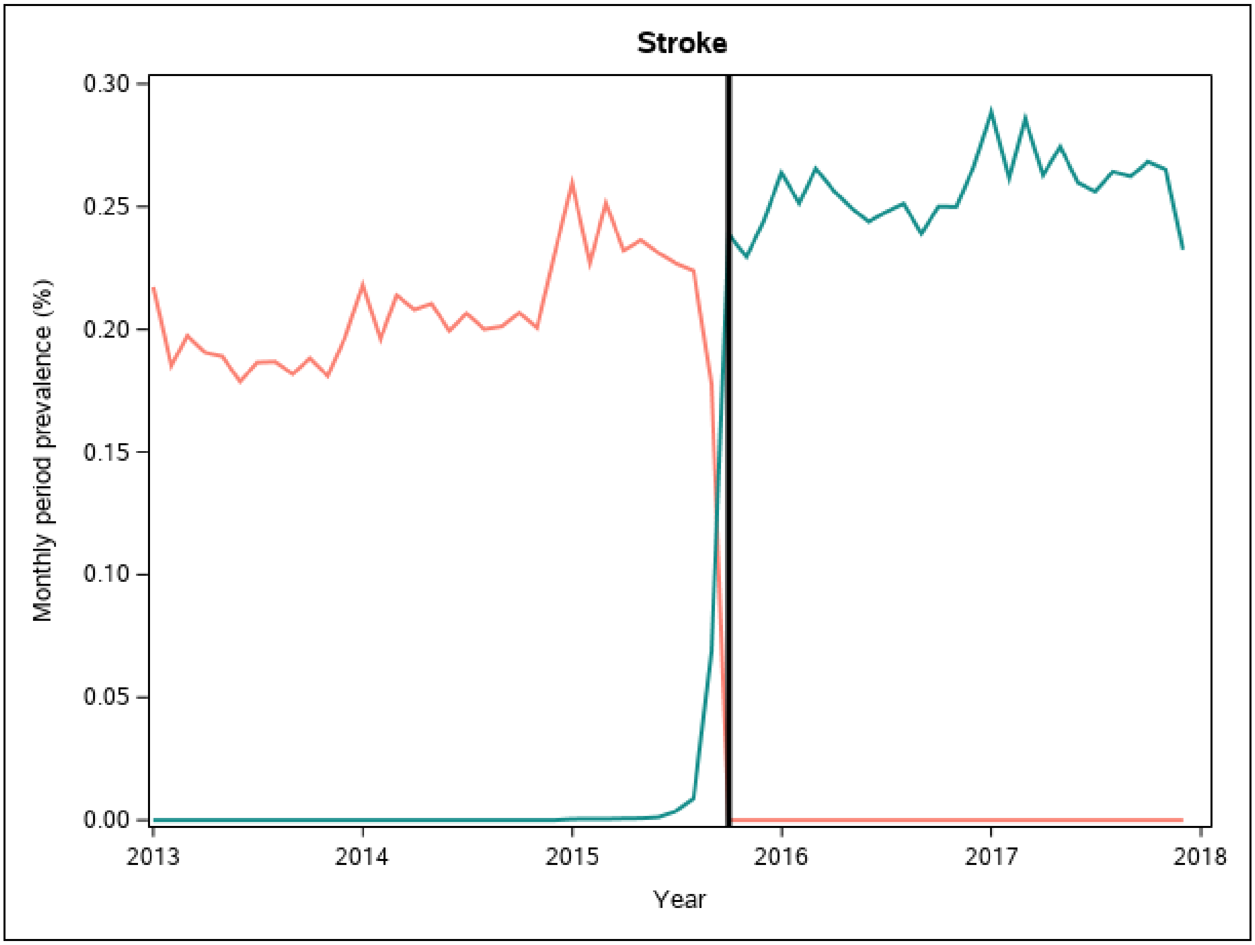

Monthly prevalence estimates were calculated across the entire 20% random sample of Medicare enrollees from 2013-2017. An individual was considered at-risk within a given month if they were enrolled in Medicare Parts and B during that month. If an individual had ≥1 claim meeting an outcome definition within that given month, they were counted as having an outcome event during that month. Prevalence estimates were then calculated by dividing the number of individuals who experienced ≥1 qualifying event within a month by the number of individuals identified as being at-risk during that month.

© 2023 Zhang J et al. *JAMA Network Open.*

Figure S3. Month-level prevalence of our primary outcome definition for in-patient hospitalization for myocardial infarction over the ICD-9 to ICD-10 transition. Estimates were calculated in the entire Medicare enrollee population from 2013-2017.

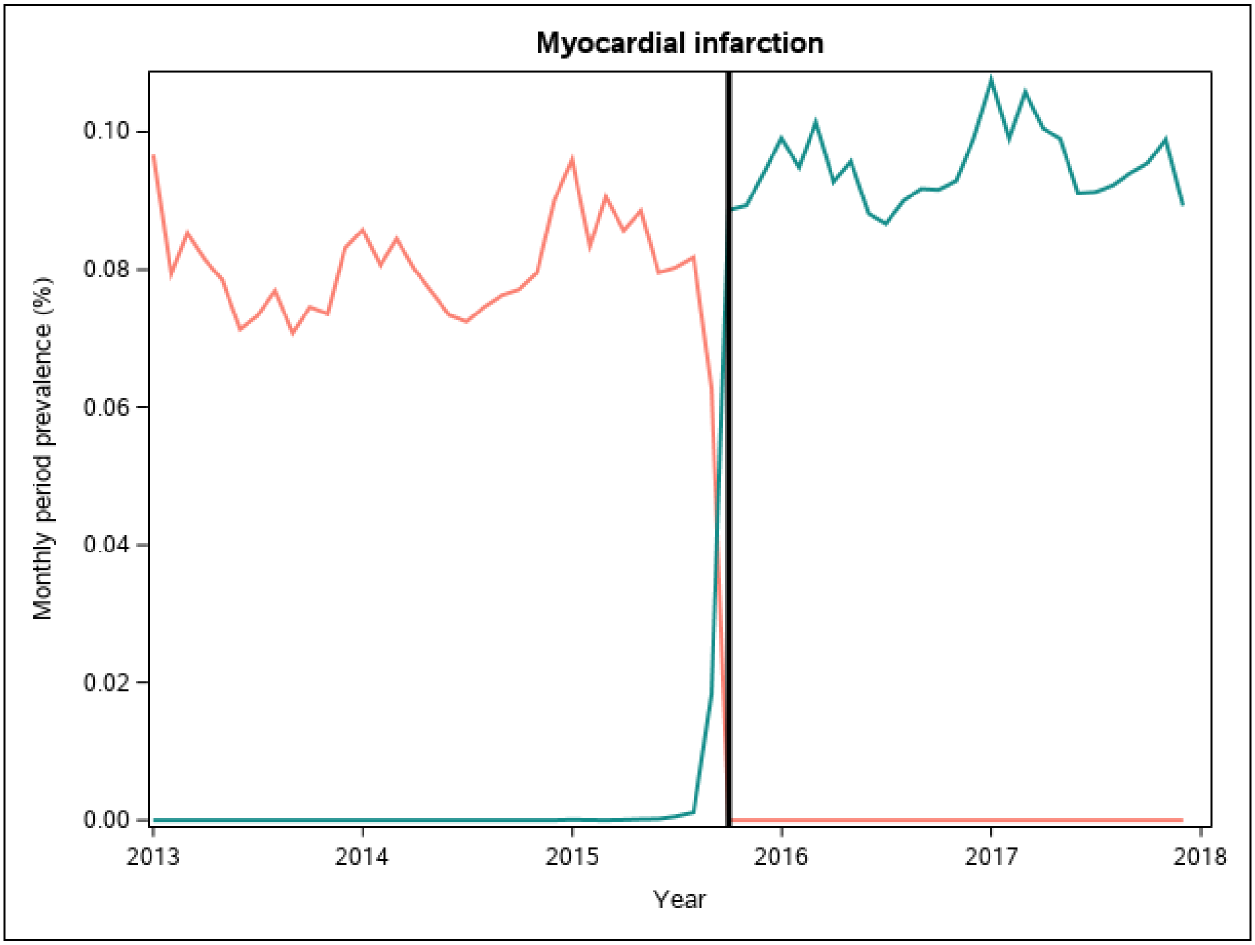

Monthly prevalence estimates were calculated across the entire 20% random sample of Medicare enrollees from 2013-2017. An individual was considered at-risk within a given month if they were enrolled in Medicare Parts and B during that month. If an individual had ≥1 claim meeting an outcome definition within that given month, they were counted as having an outcome event during that month. Prevalence estimates were then calculated by dividing the number of individuals who experienced ≥1 qualifying event within a month by the number of individuals identified as being at-risk during that month.

© 2023 Zhang J et al. *JAMA Network Open.*

Figure S4. Directed acyclic graph used to identify important potential confounders in this study.[5,6] We have explicitly included race as a proxy for systemic and interpersonal racism.

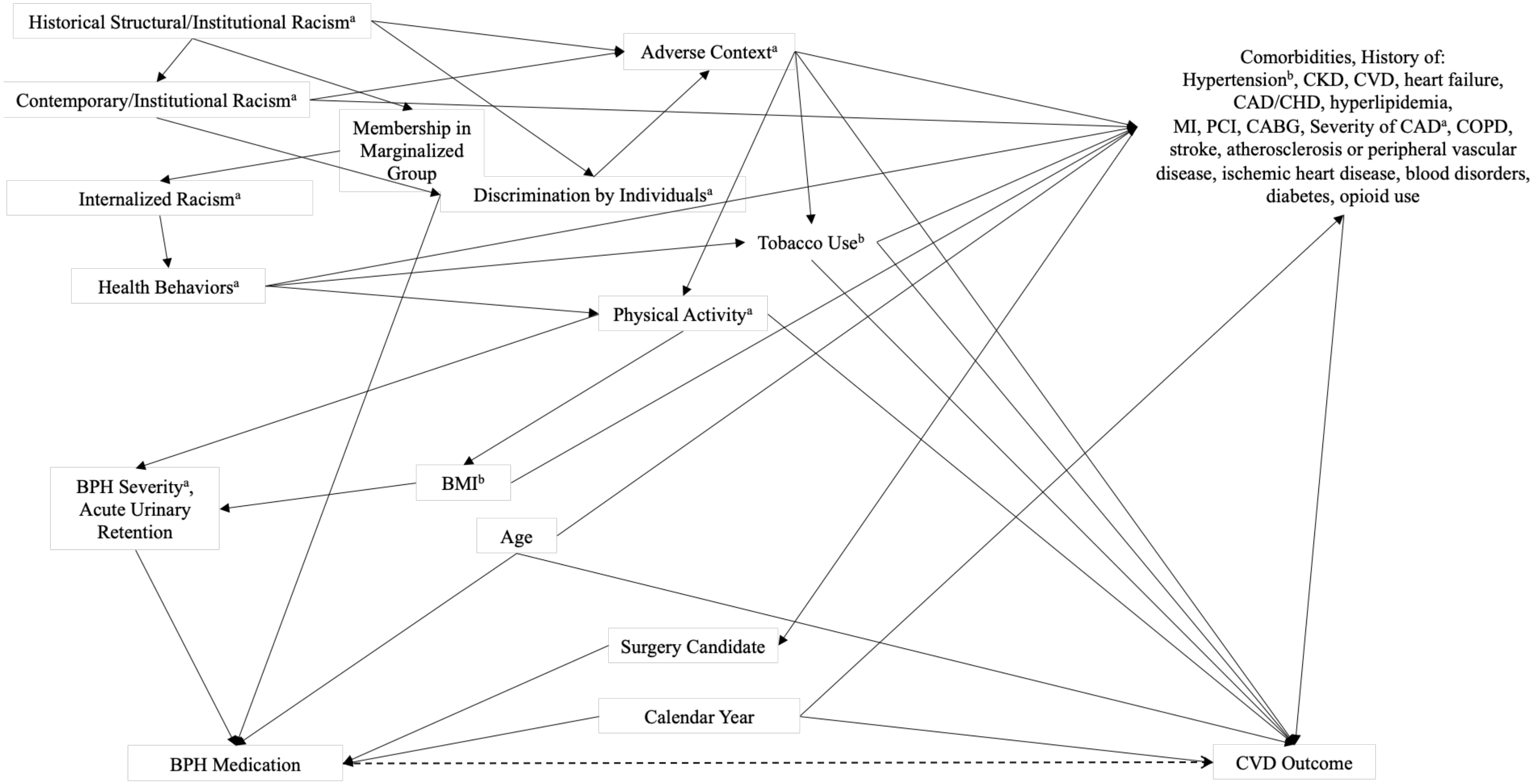

CAD/CHD = coronary artery disease/coronary heart disease. MI = myocardial infarction. PCI = percutaneous coronary intervention. COPD = chronic obstructive pulmonary disease. CKD = chronic kidney disease. CVD = cardiovascular disease.

[a] These variables are not measured in the Medicare insurance claims data.

[b] These variables are known to be poorly measured in insurance claims data.

© 2023 Zhang J et al. *JAMA Network Open.*

Table S5. Logistic regression model specification used to generate propensity scores and inverse probability of censoring weights in the primary analyses.

Variable	Specification in Model	Levels
Age	Linear	66-90 years
Calendar year	Categorical	2008, 2009, 2010, 2011, 2012, 2013, 2014, 2015 2016, 2017, 2018
Acute urinary retention	Categorical	Yes, No
Tobacco Use	Categorical	Yes, No
Coronary heart disease	Categorical	Yes, No
Inpatient hospitalization for heart failure (HF)	Categorical	Yes, No
Chronic Kidney Disease (CKD)	Categorical	Yes, No
Coronary Obstructive Pulmonary Disease (COPD)	Categorical	Yes, No
Hypercholesterolemia (HCHL)	Categorical	Yes, No
Hospitalization due to Myocardial Infarction (MI)	Categorical	Yes, No
Hospitalization due to Stroke	Categorical	Yes, No
Diabetes mellitus (DM)	Categorical	Yes, No
Interaction: HF * CKD * COPD * HCHL * MI * Stroke * DM	Categorical	Yes, No
Percutaneous coronary intervention	Categorical	Yes, No
Coronary Artery Bypass Graft Surgery	Categorical	Yes, No
Atherosclerosis	Categorical	Yes, No
ACE Inhibitors	Categorical	Yes, No
ARBs	Categorical	Yes, No
Beta blockers (BBs)	Categorical	Yes, No
Calcium channel blockers (CCBs)	Categorical	Yes, No
Thiazide diuretics	Categorical	Yes, No
Combination diuretics	Categorical	Yes, No
Potassium sparing diuretics	Categorical	Yes, No
Loop diuretics	Categorical	Yes, No
Other diuretics	Categorical	Yes, No
Interaction: ACEIs * ARBs * BBs * CCBs * Thiazide diuretics * Combination diuretics * Potassium sparing	Categorical	All combinations, Yes/No

© 2023 Zhang J et al. *JAMA Network Open.*

diuretics * Loop diuretics * Other diuretics		
Prior anticoagulant use	Categorical	Yes, No
Opioid Use	Categorical	No fill, 1 fill, ≥2 fills
Nicotine or Varenicline	Categorical	Yes, No
Statins	Categorical	Yes, No
DPP-4 inhibitors	Categorical	Yes, No
GLP-1	Categorical	Yes, No
Long-acting insulin	Categorical	No fill, 1 fill, ≥2 fills
Short-acting insulin	Categorical	No fill, 1 fill, ≥2 fills
SGLT-2 inhibitors	Categorical	Yes, No
Sulfonylureas	Categorical	Yes, No
TZD	Categorical	Yes, No
Obesity	Categorical	Yes, No
Race	Categorical	Non-Hispanic, White; Black or African American; Asian, Pacific Islander; Hispanic; American Indian/Alaska Native; Other; Unknown
Faurot Frailty Index[7,8]		
Arthritis	Categorical	Yes, No
Bladder incontinence	Categorical	Yes, No
Stroke/Brain injury	Categorical	Yes, No
Skin ulcer	Categorical	Yes, No
Dementias	Categorical	Yes, No
Hypotensive shock; sepsis	Categorical	Yes, No
Lipid abnormalities	Categorical	Yes, No
Paralysis	Categorical	Yes, No
Parkinson's Disease	Categorical	Yes, No
Podiatric care	Categorical	Yes, No
Psychiatric illness	Categorical	Yes, No
Cancer Screening	Categorical	Yes, No
Vertigo	Categorical	Yes, No
Weakness	Categorical	Yes, No
Ambulance transport	Categorical	Yes, No
Home hospital bed	Categorical	Yes, No
Outpatient visit	Categorical	Yes, No
Rehabilitation care	Categorical	Yes, No
Home Oxygen	Categorical	Yes, No
Wheelchair	Categorical	Yes, No

© 2023 Zhang J et al. *JAMA Network Open.*

Table S6. Diagnosis code list to identify hospitalization due to injury or poisoning as a negative control outcome.

**Variable**	**Implementation**	**ICD-9-CM Diagnosis Codes**	**ICD-10-CM Diagnosis Codes**
Hospitalization for injury or poisoning[9]	≥1 code in the first or second position of the discharge diagnoses on an inpatient claim	800-999	Forward-backward general equivalence mapping (https://github.com/alankinlaw/Easy_ICD9-to-10_GEMs_mapping),[10] full code list on GitHub (https://github.com/chasedlatour/AB-v-5ARI-Medicare).

Abbreviations: ICD-9-CM=International Classification of Diseases, 9th Revision, Clinical Modification; ICD-10-CM: International Classification of Diseases, 10th Revision, Clinical Modification

© 2023 Zhang J et al. *JAMA Network Open.*

Method S1. Quantitative bias analysis for smoking and obesity.

We saw substantial imbalance on obesity and tobacco use in our crude cohort. Further, we know that these variables tend to be poorly classified in insurance claims data; this is illustrated by the low sensitivity for these variables in the algorithms that we used.

For diagnosis codes for obesity or severe obesity, Suissa et al. 2021 found a sensitivity of 40.4% and 97.0% in Medicare claims data that they linked to the Mass General Brigham Research Patient Data Repository.[3] In this study, they constructed a cohort of patients who had ≥1 measurement of their body mass index (BMI) between January 1, 2014 – June 30, 2014 or January 1, 2016 – June 30, 2016. Further, patients were required to have ≥1 year of continuous enrollment in Medicare before and after their BMI measurement in the electronic health record (EHR) data. Finally, the investigators excluded patients who had implausible BMI measurements in the EHR data or were pregnant prior to cohort entry. These data are not directly comparable to our cohort; however, age-stratified bias parameters represent our best estimates of the misclassification in our overweight or obesity variable: sensitivity = 36.0%, specificity = 97.3%.

For our algorithm for tobacco use (incorporating both diagnosis codes and prescription claims), Desai et al. 2016 found a sensitivity of 16.4% and specificity of 100% among a cohort of patients who were part of the Brigham and Women's Hospital Rheumatoid Arthritis Sequential Study (BRASS) and who were enrolled in Medicare between 2006 and 2010. To be included in the study, patients had to be BRASS participants and have ≥365 days of continuous enrollment in Medicare prior to a valid measure of smoking status. These patients are clearly not representative of the

© 2023 Zhang J et al. *JAMA Network Open.*

study cohort, but at least use the same claims data source. The investigators did not provide bias parameters stratified by patient demographic characteristics. As a result, we planned to use the aforementioned bias parameters as our best estimates of the misclassification of our smoking/tobacco use variable. However, that low of sensitivity bias parameters could not be supported by the software; instead, we used a sensitivity value, among those with MACE, that was as low as was allowed allowed by the methodology to get valid results.

We use methodology by Lash et al. 2009 to investigate how residual confounding by these variables may impact our estimate of the effect of ABs versus 5ARIs on MACE outcomes (https://sites.google.com/site/biasanalysis/Home).[11] We completed these analyses for each variable individually, as we did not have estimates of bias parameters stratified by these variables. To do this, we first calculated the study effect estimates stratified by the confounder without bootstrapping. We then prespecified the bias parameters. In one scenario, we assumed non-differential misclassification by MACE status. In a second scenario, we assume that individuals with MACE outcomes had a higher sensitivity for the confounders than individuals without MACE. This is based in the assumption that individuals with additional risk factors for MACE may be more likely to have these variables recorded in Medicare claims data than individuals with fewer risk factors for MACE.

© 2023 Zhang J et al. *JAMA Network Open.*

Figure S5. Diagram demonstrating the flow of study patients and new-use episodes through inclusion and exclusion criteria.

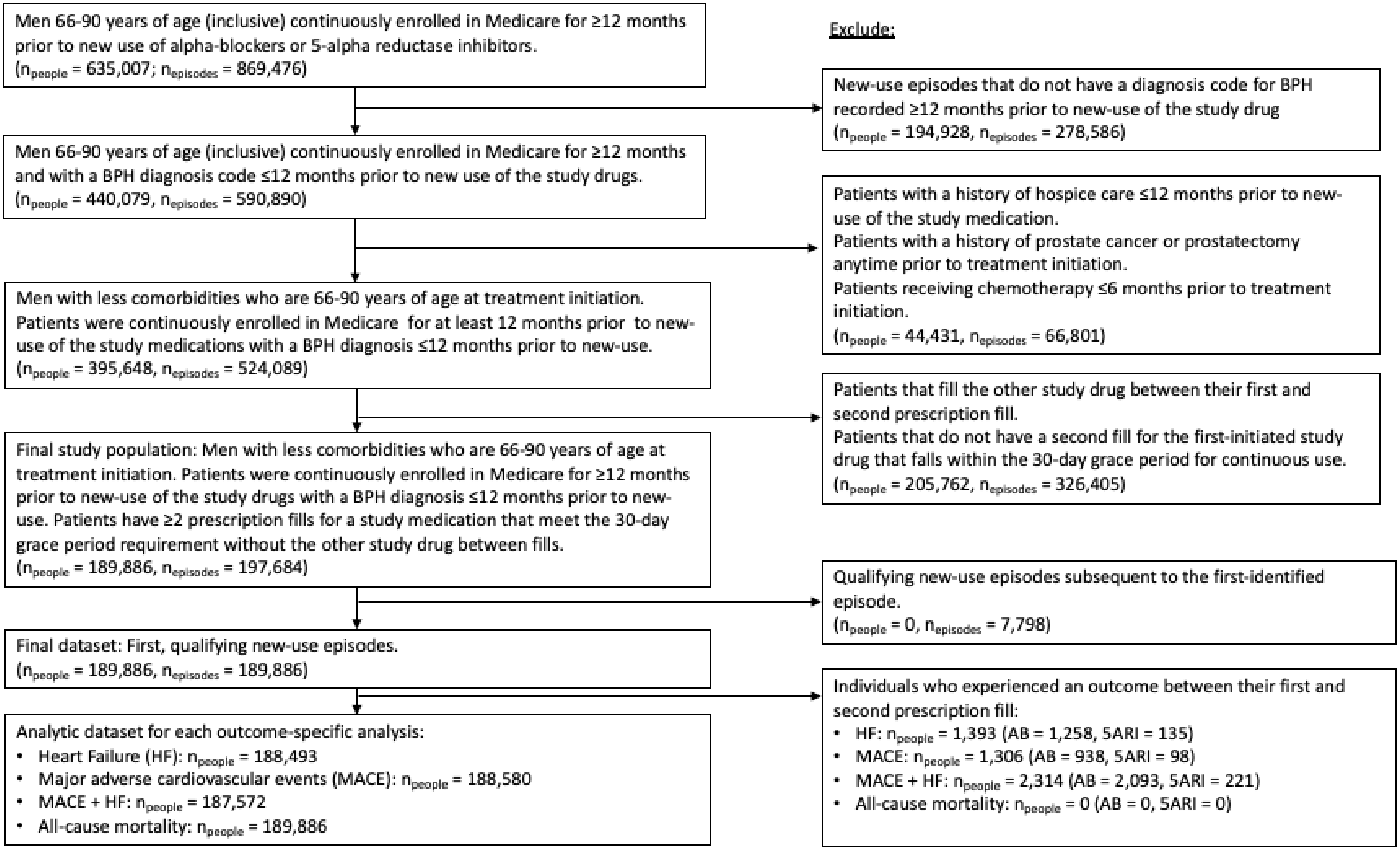

© 2023 Zhang J et al. *JAMA Network Open.*

Figure S6. Annual estimates of the proportion of new-use episodes attributed to each of the study drugs in the primary study population.

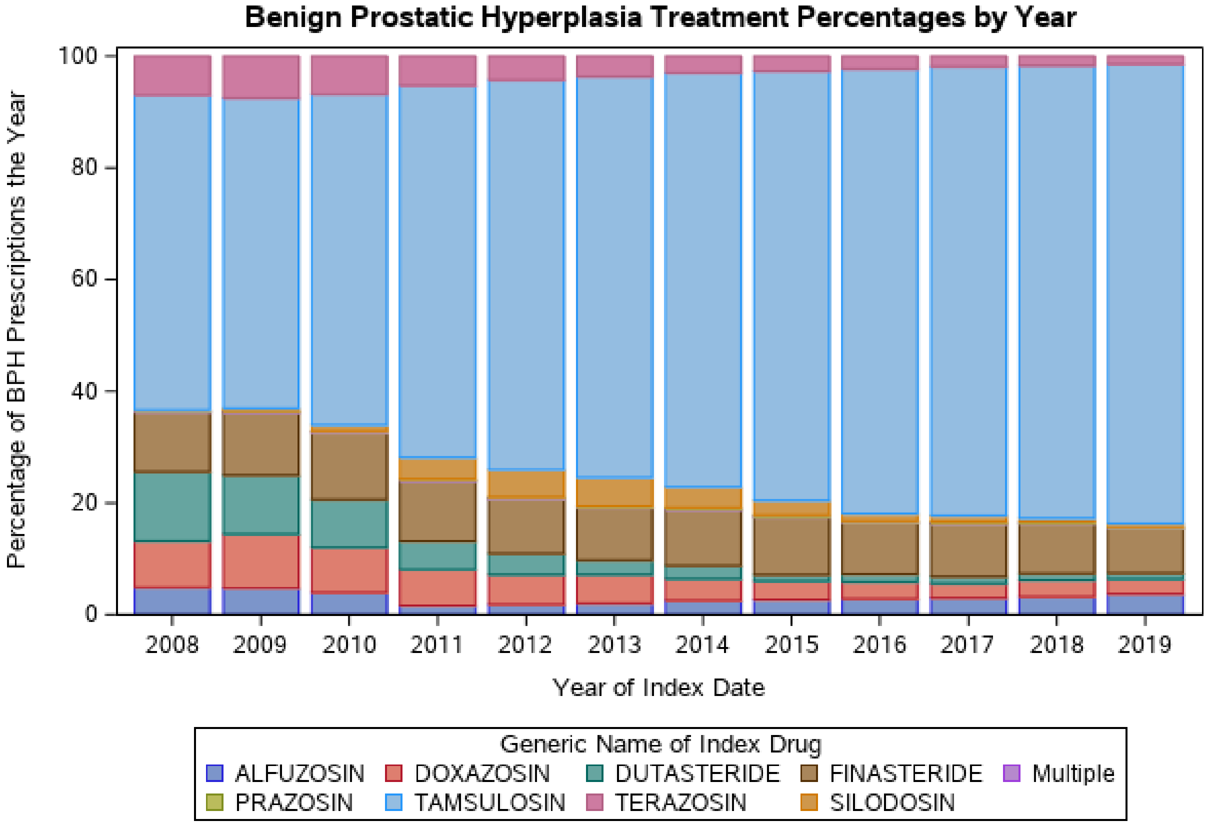

© 2023 Zhang J et al. *JAMA Network Open.*

Figure S7. Propensity score distributions prior to trimming patients. Data includes patient population without bootstrapping.

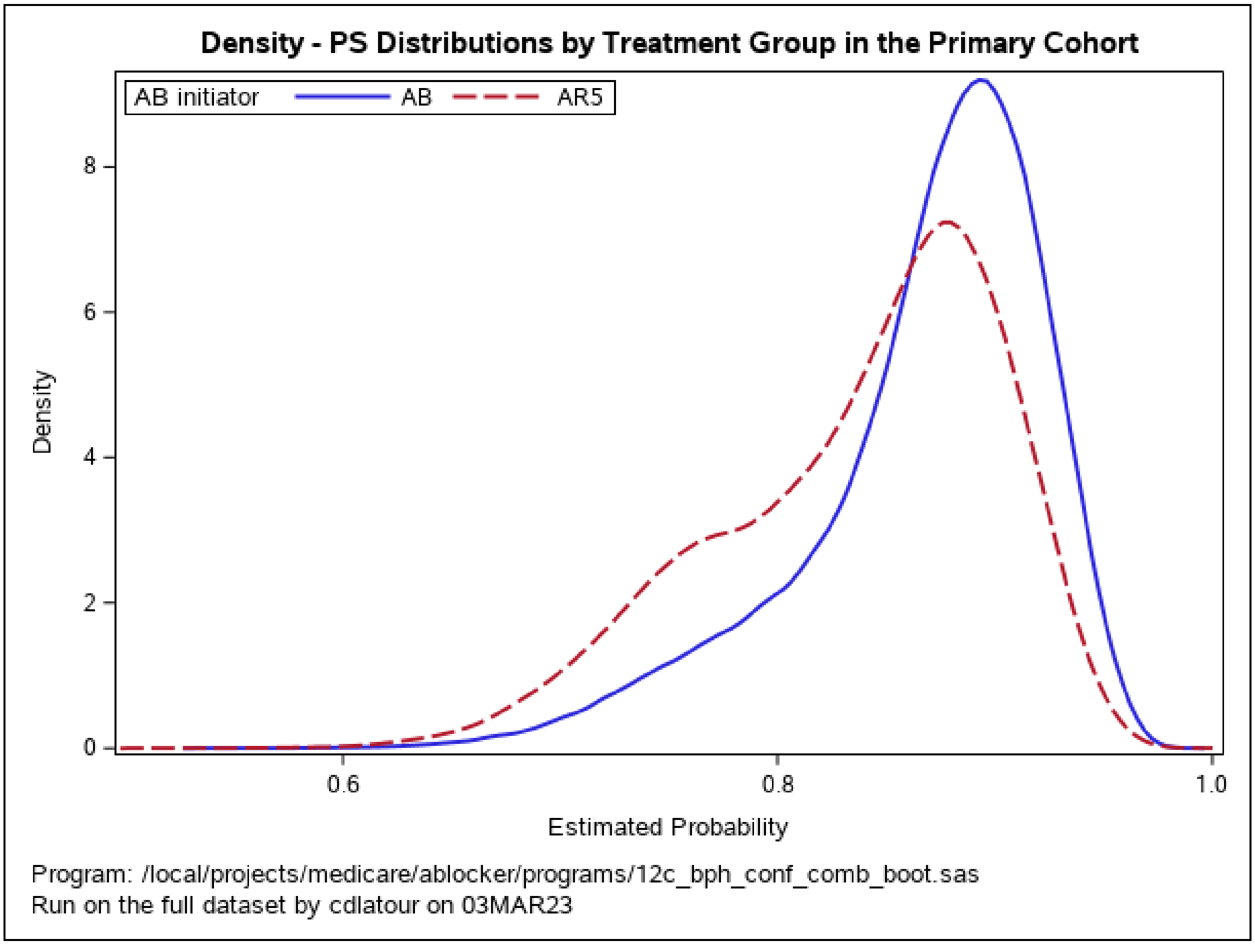

© 2023 Zhang J et al. *JAMA Network Open.*

Figure S8. Propensity score distributions, after trimming non-overlapping propensity scores and re-fitting the logistic regression model, in the included patient population without bootstrapping.

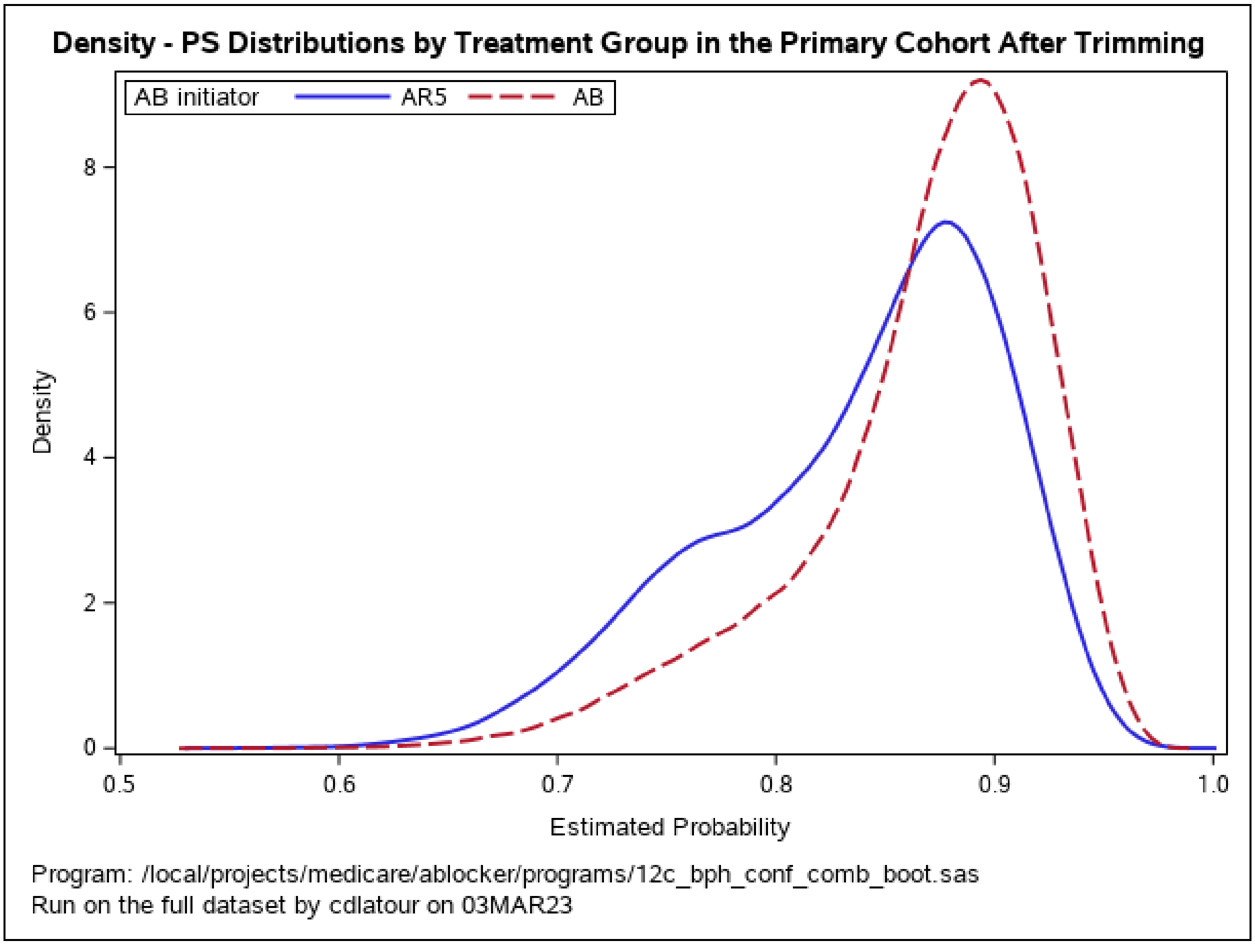

© 2023 Zhang J et al. *JAMA Network Open.*

Table S7. Descriptive statistics of the stabilized inverse probability of treatment weights in the primary patient population without bootstrapping.

Treatment Group	Mean	Minimum	Maximum
Alpha-Blockers	1.0000655	0.8849867	1.5962461
5-Alpha Reductase Inhibitors	0.9988374	0.3084562	5.5334686
Overall	0.9998971	0.3084562	5.5334686

© 2023 Zhang J et al. *JAMA Network Open.*

Figure S9. Histogram of the number of days from the second fill date until an individual (1) discontinued (with a 30-day grace period to define continuous use), (2) filled a prescription for the other drug class, or (3) were censored from the analysis, whichever occurred first.

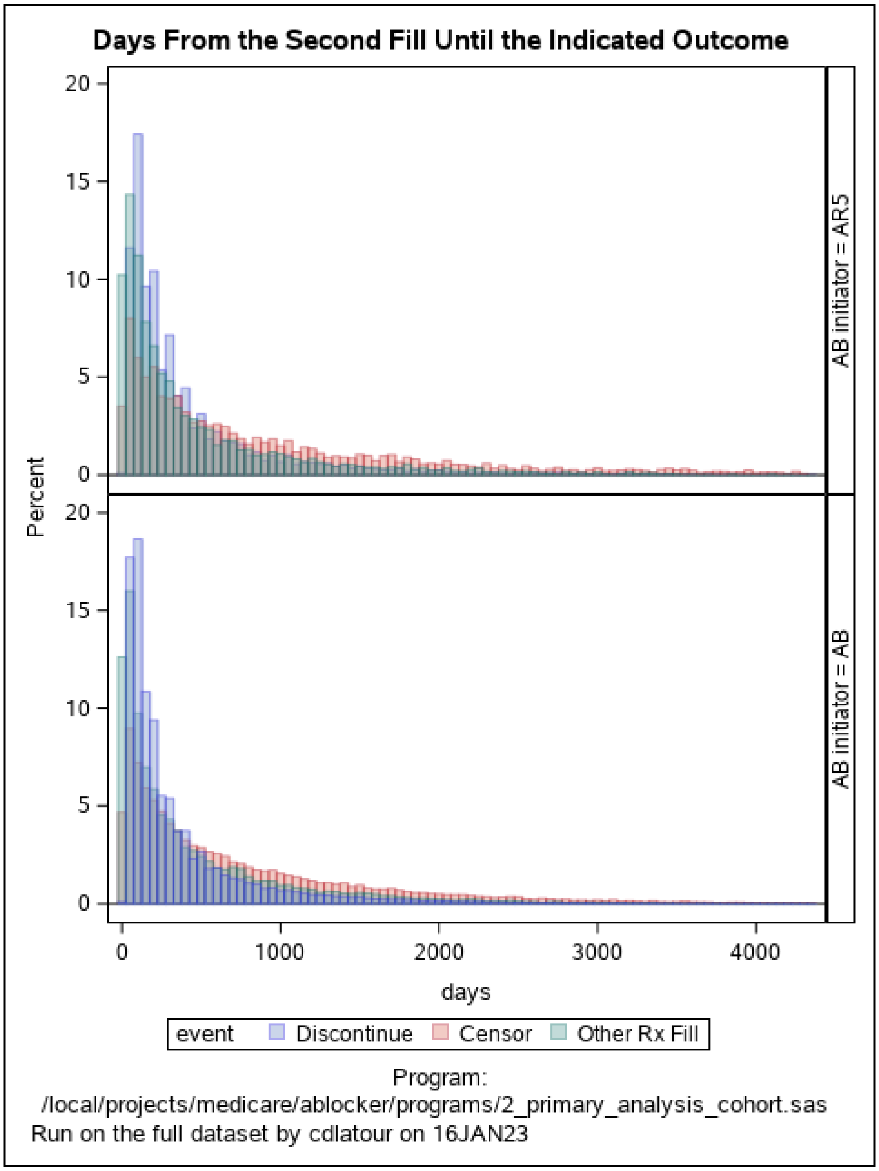

© 2023 Zhang J et al. *JAMA Network Open.*

Table S8. Counts and percentages of medications attributed to new-use episodes, stratified by year in the primary study population.

Medication	Year of the Index Visit Number of Visits Attributed to Each Medication											
	**2008**	**2009**	**2010**	**2011**	**2012**	**2013**	**2014**	**2015**	**2016**	**2017**	**2018**	**2019**
5-Alpha Reductase Inhibitors												
Dutasteride	1561	1387	1173	650	513	405	412	206	256	230	210	169
Finasteride	1356	1477	1634	1381	1328	1464	1720	1938	1751	1831	1662	1310
Multiple	4	2	2	0	2	0	1	0	0	4	0	1
Alpha-Blockers												
Alfuzosin	620	629	543	215	255	322	444	509	541	580	594	605
Doxazosin	1052	1268	1110	823	719	787	665	618	572	503	550	433
Multiple	24	21	25	23	23	16	32	19	15	18	10	17
Prazosin	22	19	16	16	28	20	38	41	23	38	40	35
Silodosin[a]	0	65	155	492	665	786	636	491	248	215	109	90
Tamsulosin[a]	7123	7299	8108	8517	9483	11098	12783	14354	14984	15462	14880	13355
Terazosin	879	987	930	668	579	565	506	491	422	353	304	228

[a] Selective antagonists of the alpha-1A adrenergic receptor subtype.

© 2023 Zhang J et al. *JAMA Network Open.*

Table S9. Descriptive statistics of days from the second prescription fill until discontinuation, filling a prescription for the other study drug, or censoring, by initial treatment and amount of follow-up.

		Number of Persons at Baseline	Number of Days of Follow-up	Number and Percentage of Events that Occurred by 1 Year of Follow-up	Number and Percentage of Events that Occurred by 2 Years of Follow-up	Number and Percentage of Events that Occurred by 3 Years of Follow-up
Treatment Arm	First Event (Discontinuation, Other Study Drug, or Censoring) To Occur During Follow-up	N (% of Treatment Arm)	Median (IQR)	N (% of People in Row)	N (% of People in Row)	N (% of People in Row)
Alpha-Blockers (N = 163,846)	Discontinuation	96,492 (59%)	189 (99, 424)	68,207 (71%)	83,467 (87%)	89,481 (93%)
	Prescription Fill for the Other Study Drug	18,223 (11%)	213 (60, 605)	11,498 (63%)	14,492 (80%)	16,063 (88%)
	Censored	49,131 (30%)	460 (159, 1,056)	21,625 (44%)	31,326 (64%)	37,363 (76%)
5-Alpha Reductase Inhibitors (N = 26,040)	Discontinuation	14,383 (55%)	229 (120, 512)	9,350 (65%)	11,978 (83%)	13,019 (91%)
	Prescription Fill for the Other Study Drug	4,973 (19%)	222 (76, 600)	3,131 (63%)	3,950 (79%)	4,343 (87%)
	Censored	6,684 (26%)	552 (196, 1,254)	2,630 (39%)	3,911 (59%)	4,739 (71%)

IQR = Interquartile range.
This table includes individual who are later removed from the analysis during propensity score trimming.

© 2023 Zhang J et al. *JAMA Network Open.*

Supplemental Table S10. Primary study results after 1-year of follow-up, conducted among the patient population included after asymmetric propensity score trimming.

	Inverse Probability of Treatment Weighted			
**Study Outcome**	**Risk Among α-Blocker Initiators**	**Risk Among 5α-Reductase Inhibitors**	**Risk Difference Per 1,000 (95% CI)**	**Risk Ratio (95% CI)**
Hospitalization for Heart Failure	3.41<br>(3.29, 3.52)	3.28<br>(3.00, 3.55)	0.13<br>(-1.59, 4.22)	1.04<br>(0.95, 1.14)
MACE Outcomes	8.23<br>(8.05, 8.42)	7.68<br>(7.27, 8.10)	5.47<br>(1.24, 9.71)	1.07<br>(1.01, 1.13)
Composite MACE or Hospitalization for Heart Failure	10.23<br>(10.02, 10.43)	9.50<br>(9.06, 9.94)	7.28<br>(2.72, 11.84)	1.08<br>(1.03, 1.13)
Death from Any Cause	5.55<br>(5.41, 5.70)	5.13<br>(4.79, 5.48)	4.17 pe<br>(0.61, 7.73)	1.08<br>(1.01, 1.16)

© 2023 Zhang J et al. *JAMA Network Open.*

Table S11. Descriptive table of the study population, limited to those new-use episodes by new-users of alpha-blockers that are attributed to tamsulosin or silodosin only.

	Pre-Weighting Cohort After PS Trimming of Non-Overlapping Regions			Cohort After Applying Inverse Probability of Treatment Weights		
	Prevalence Among α-Blocker Initiators (N = 141,382)	Prevalence Among 5α-Reductase Inhibitor Initiators (N = 26,039)		Prevalence Among α-Blocker Initiators	Prevalence Among 5α-Reductase Inhibitor Initiators	
**Confounder Variable**	**N (%)**	**N (%)**	**SMD**	**%**	**%**	**SMD**
Age, Median (IQR)	74.0 (69.0-79.0)	74.0 (70.0-80.0)	0.107	74.0 (69.0-79.0)	74.0 (70.0-79.0)	0.020
Calendar Year, Median (IQR)	2015.0 (2012.0-2017.0)	2013.0 (2010.0-2016.0)	0.414	2015.0 (2011.0-2017.0)	2014.0 (2011.0-2017.0)	0.003
Race/Ethnicity[a]						
Unknown	1,932 (1.4%)	286 (1.1%)	0.024	1.3%	1.3%	0.002
Non-Hispanic, White	115,759 (81.9%)	21,605 (83.0%)	0.029	82.0%	81.5%	0.013
Black or African American	7,794 (5.5%)	1,339 (5.1%)	0.016	5.5%	5.7%	0.010
Other	1,630 (1.2%)	250 (1.0%)	0.019	1.1%	1.1%	0.002
Asian/Pacific Islander	4,947 (3.5%)	827 (3.2%)	0.018	3.5%	3.5%	0.005
Hispanic	8,890 (6.3%)	1,656 (6.4%)	0.003	6.3%	6.4%	0.006
American Indian/Alaska Native	430 (0.3%)	76 (0.3%)	0.002	0.3%	0.3%	0.004
Acute Urinary Retention	33,130 (23.4%)	4,574 (17.6%)	0.146	22.5%	22.6%	0.001
Coronary Heart Disease	58,625 (41.5%)	10,443 (40.1%)	0.028	41.3%	41.7%	0.008
Hospitalization due to Heart Failure	10,889 (7.7%)	1,439 (5.5%)	0.088	7.4%	7.7%	0.014
Chronic Kidney Disease	42,329 (29.9%)	6,208 (23.8%)	0.138	29.0%	29.4%	0.010
COPD	34,662 (24.5%)	5,385 (20.7%)	0.092	23.9%	24.3%	0.008
Hypercholesterolemia	67,988 (48.1%)	14,971 (57.5%)	0.189	49.6%	49.6%	0.001
Hospitalization due to myocardial infarction	3,018 (2.1%)	427 (1.6%)	0.036	2.1%	2.2%	0.008
Hospitalization due to Stroke	4,974 (3.5%)	612 (2.4%)	0.069	3.3%	3.5%	0.010
Percutaneous Coronary Intervention	2,523 (1.8%)	437 (1.7%)	0.008	1.8%	1.9%	0.007
Coronary Artery Bypass Graft Surgery	1,516 (1.1%)	152 (0.6%)	0.054	1.0%	1.0%	0.001
Tobacco Use	26,917 (19.0%)	3,671 (14.1%)	0.133	18.3%	18.3%	0.001
ACE Inhibitor, Any Use	52,883 (37.4%)	9,258 (35.6%)	0.038	37.1%	37.0%	0.003
ARB, Any Use	29,151 (20.6%)	5,072 (19.5%)	0.028	20.4%	20.6%	0.004

© 2023 Zhang J et al. *JAMA Network Open.*

Beta-Blocker, Any Use	64,162 (45.4%)	10,993 (42.2%)	0.064	44.9%	45.0%	0.003
Peripheral Vasodilators, Any Use	2,588 (1.8%)	609 (2.3%)	0.036	1.9%	1.9%	0.003
Calcium Channel Blocker, Any use	42,031 (29.7%)	6,877 (26.4%)	0.074	29.2%	29.2%	0.001
Thiazide Diuretics, Any Use	29,529 (20.9%)	5,250 (20.2%)	0.018	20.8%	20.5%	0.007
Combination Diuretics, Any Use	15,462 (10.9%)	2,920 (11.2%)	0.009	11.0%	10.8%	0.005
Potassium Sparing Diuretic, Any Use	7,904 (5.6%)	1,391 (5.3%)	0.011	5.6%	5.7%	0.004
Loop Diuretic, Any Use	22,259 (15.7%)	3,593 (13.8%)	0.055	15.5%	15.8%	0.011
Other Diuretics, Any Use	3,033 (2.1%)	453 (1.7%)	0.029	2.1%	2.1%	0.001
Anticoagulant Use						
No Fill	121,331 (85.8%)	22,111 (84.9%)	0.026	85.7%	85.4%	0.006
1 Fill	3,757 (2.7%)	532 (2.0%)	0.041	2.6%	2.6%	0.001
≥2 Fills	16,294 (11.5%)	3,396 (13.0%)	0.046	11.8%	12.0%	0.007
Opioid Use						
No Fill	83,205 (58.9%)	17,178 (66.0%)	0.147	60.0%	59.9%	0.001
1 Fill	23,310 (16.5%)	3,958 (15.2%)	0.035	16.3%	16.2%	0.001
≥2 Fills	34,867 (24.7%)	4,903 (18.8%)	0.142	23.8%	23.9%	0.003
Nicotine or Varenicline, Any Use	672 (0.5%)	106 (0.4%)	0.010	0.5%	0.5%	0.002
Statin, Any Use	84,112 (59.5%)	14,652 (56.3%)	0.065	59.0%	58.8%	0.003
Diabetes	47,073 (33.3%)	7,530 (28.9%)	0.095	32.6%	32.9%	0.006
DPP-4i	6,099 (4.3%)	840 (3.2%)	0.057	4.1%	4.1%	0.001
GLP-1	1,446 (1.0%)	164 (0.6%)	0.043	1.0%	1.0%	0.003
Long-Acting Insulin						
No Fill	131,037 (92.7%)	24,626 (94.6%)	0.077	93.0%	92.8%	0.007
1 Fill	1,450 (1.0%)	183 (0.7%)	0.035	1.0%	1.0%	0.001
≥2 Fills	8,895 (6.3%)	1,230 (4.7%)	0.069	6.1%	6.2%	0.007
Short-Acting Insulin						
No Fill	135,998 (96.2%)	25,320 (97.2%)	0.059	96.4%	96.2%	0.007
1 Fill	1,505 (1.1%)	186 (0.7%)	0.037	1.0%	1.0%	0.002
≥2 Fills	3,879 (2.7%)	533 (2.0%)	0.046	2.6%	2.8%	0.007
SGLT-2i, Any Fill	1,050 (0.7%)	108 (0.4%)	0.043	0.7%	0.7%	0.002
Sulfonylureas, Any Fill	15,556 (11.0%)	2,366 (9.1%)	0.064	10.7%	10.7%	0.001
TZD, Any Fill	4,218 (3.0%)	784 (3.0%)	0.002	3.0%	3.0%	0.001

© 2023 Zhang J et al. *JAMA Network Open.*

Atherosclerosis or Peripheral Vascular Disease	38,823 (27.5%)	8,020 (30.8%)	0.074	28.0%	28.4%	0.008
Obesity	17,032 (12.0%)	1,941 (7.5%)	0.155	11.3%	11.5%	0.004

We assessed adequate covariate balance before and after IPTW using standardized mean differences, using a threshold of ≤0.1 to indicate adequate balance. The sum of the standardized weights in the AB group was 163,838, and the sum in the 5ARI group was 26,010.

© 2023 Zhang J et al. *JAMA Network Open.*

Table S12. Primary study results after 1-year of follow-up, limited to those new-use episodes among alpha-blockers that are attributed to only tamsulosin and silodosin, selective alpha-1A adrenergic receptor antagonists.

	Non-IPTW Estimates after PS Trimming				Inverse Probability of Treatment Weighted After PS Trimming			
**Study Outcome**	**Risk Among α-Blocker Initiators % (95% CI)**	**Risk Among 5α-Reductase Inhibitor Initiators % (95% CI)**	**Risk Difference Per 1,000 (95% CI)**	**Risk Ratio (95% CI)**	**Risk Among α-Blocker Initiators % (95% CI)**	**Risk Among 5α-Reductase Inhibitors % (95% CI)**	**Risk Difference (95% CI)**	**Risk Ratio (95% CI)**
Hospitalization for Heart Failure	4.01 (3.90, 4.13)	3.28 (3.06, 3.50)	7.35 (4.91, 9.79)	1.22 (1.14, 1.32)	3.93 (3.82, 4.04)	3.98 (3.69, 4.26)	-0.46 (-3.43, 2.52)	0.99 (0.92,1.07)
MACE Outcomes	9.37 (9.21, 9.53)	7.40 (7.09, 7.71)	19.68 (16.24, 23.11)	1.27 (1.21, 1.32)	9.20 (9.04, 9.35)	8.59 (8.19, 8.99)	6.04 (1.78,10.29)	1.07 (1.02, 1.12)
Composite MACE or Hospitalization for Heart Failure	11.63 (11.46, 11.81)	9.28 (8.93, 9.64)	23.52 (19.56, 27.48)	1.25 (1.20, 1.31)	11.43 (11.25, 11.60)	10.72 (10.28, 11.16)	7.05 (2.32, 11.78)	1.07 (1.02, 1.11)
Death from Any Cause	6.35 (6.22, 6.48)	4.97 (4.70, 5.23)	13.87 (10.99, 16.75)	1.28 (1.21, 1.35)	6.24 (6.11, 6.37)	5.86 (5.53, 6.19)	3.80 (0.33, 7.27)	1.07 (1.00, 1.13)

© 2023 Zhang J et al. *JAMA Network Open.*

Table S13. Descriptive statistics of the unweighted patient population with a BPH diagnosis in the 180 days prior to their new-use episode.

Covariate Value	Prevalence Among a-Blocker Initiators (N = 152,537) N (%)	Prevalence Among 5a-Reductase Inhibitor Initiators (N = 24,396) N (%)	SMD
Age, Median (IQR)	73.0 (69.0-79.0)	74.0 (70.0-80.0)	0.118
Calendar Year, Median (IQR)	2015.0 (2012.0-2017.0)	2013.0 (2010.0-2016.0)	0.350
Race/ Ethnicity[a]			0.072
Unknown	2,068 (1.4%)	273 (1.1%)	
Non-Hispanic, White	124,300 (81.5%)	20,240 (83.0%)	
Black or African American	8,516 (5.6%)	1,262 (5.2%)	
Other	1,727 (1.1%)	237 (1.0%)	
Asian/Pacific Islander	5,479 (3.6%)	769 (3.2%)	
Hispanic	9,911 (6.5%)	1,545 (6.3%)	
American Indian/Alaska Native	536 (0.4%)	70 (0.3%)	
Acute Urinary Retention	34,468 (22.6%)	4,358 (17.9%)	0.118
Coronary Heart Disease	62,064 (40.7%)	9,740 (39.9%)	0.016
Hospitalization due to Heart Failure	11,170 (7.3%)	1,347 (5.5%)	0.074
Chronic Kidney Disease	44,429 (29.1%)	5,847 (24.0%)	0.117
COPD	36,751 (24.1%)	5,042 (20.7%)	0.082
Hypercholesterolemia	74,214 (48.7%)	13,946 (57.2%)	0.171
Hospitalization due to MI	3,091 (2.0%)	404 (1.7%)	0.028
Hospitalization due to Stroke	5,184 (3.4%)	576 (2.4%)	0.062
Percutaneous Coronary Intervention	2,569 (1.7%)	417 (1.7%)	0.002
Coronary Artery Bypass Graft Surgery	1,523 (1.0%)	141 (0.6%)	0.048
Tobacco Use	28,270 (18.5%)	3,460 (14.2%)	0.118
ACE Inhibitor, Any Use	57,220 (37.5%)	8,677 (35.6%)	0.040
ARB, Any Use	31,442 (20.6%)	4,720 (19.3%)	0.032
Beta-Blocker, Any Use	68,629 (45.0%)	10,300 (42.2%)	0.056
Peripheral Vasodilators, Any Use	2,771 (1.8%)	567 (2.3%)	0.036
Calcium Channel Blocker, Any use	46,098 (30.2%)	6,469 (26.5%)	0.082
Thiazide Diuretics, Any Use	32,380 (21.2%)	4,935 (20.2%)	0.025
Combination Diuretics, Any Use	16,804 (11.0%)	2,741 (11.2%)	0.007
Potassium Sparing Diuretic, Any Use	8,428 (5.5%)	1,287 (5.3%)	0.011
Loop Diuretic, Any Use	23,438 (15.4%)	3,360 (13.8%)	0.045
Other Diuretics, Any Use	3,251 (2.1%)	413 (1.7%)	0.032
Anticoagulant Use			0.086
No Fill	131,578 (86.3%)	20,711 (84.9%)	
1 Fill	3,923 (2.6%)	502 (2.1%)	
≥2 Fills	17,036 (11.2%)	3,183 (13.0%)	
Opioid Use			0.134
No Fill	91,179 (59.8%)	16,099 (66.0%)	
1 Fill	24,555 (16.1%)	3,699 (15.2%)	

© 2023 Zhang J et al. *JAMA Network Open.*

≥2 Fills	36,803 (24.1%)	4,598 (18.8%)	
Nicotine or Varenicline, Any Use	696 (0.5%)	99 (0.4%)	0.008
Statin, Any Use	89,865 (58.9%)	13,762 (56.4%)	0.051
Diabetes	50,252 (32.9%)	7,037 (28.8%)	0.089
DPP-4i	6,339 (4.2%)	785 (3.2%)	0.050
GLP-1	1,509 (1.0%)	154 (0.6%)	0.040
Long-Acting Insulin			0.092
No Fill	141,597 (92.8%)	23,069 (94.6%)	
1 Fill	1,544 (1.0%)	163 (0.7%)	
≥2 Fills	9,396 (6.2%)	1,164 (4.8%)	
Short-Acting Insulin			0.064
No Fill	146,920 (96.3%)	23,716 (97.2%)	
1 Fill	1,572 (1.0%)	176 (0.7%)	
≥2 Fills	4,045 (2.7%)	504 (2.1%)	
SGLT-2i, Any Fill	1,082 (0.7%)	105 (0.4%)	0.037
Sulfonylureas, Any Fill	16,660 (10.9%)	2,222 (9.1%)	0.060
TZD, Any Fill	4,576 (3.0%)	722 (3.0%)	0.002
Atherosclerosis or Peripheral Vascular Disease	41,612 (27.3%)	7,438 (30.5%)	0.071
Obesity	17,754 (11.6%)	1,827 (7.5%)	0.141

This population does not exclude individuals in non-overlapping regions of the propensity score distribution, as propensity scores were not constructed in this population.

© 2023 Zhang J et al. *JAMA Network Open.*

Figure S10. Distribution of medication fill at an individual's index date into the cohort across calendar years. Individuals must have a diagnosis code for benign prostatic hyperplasia within 6 months prior to the index date.

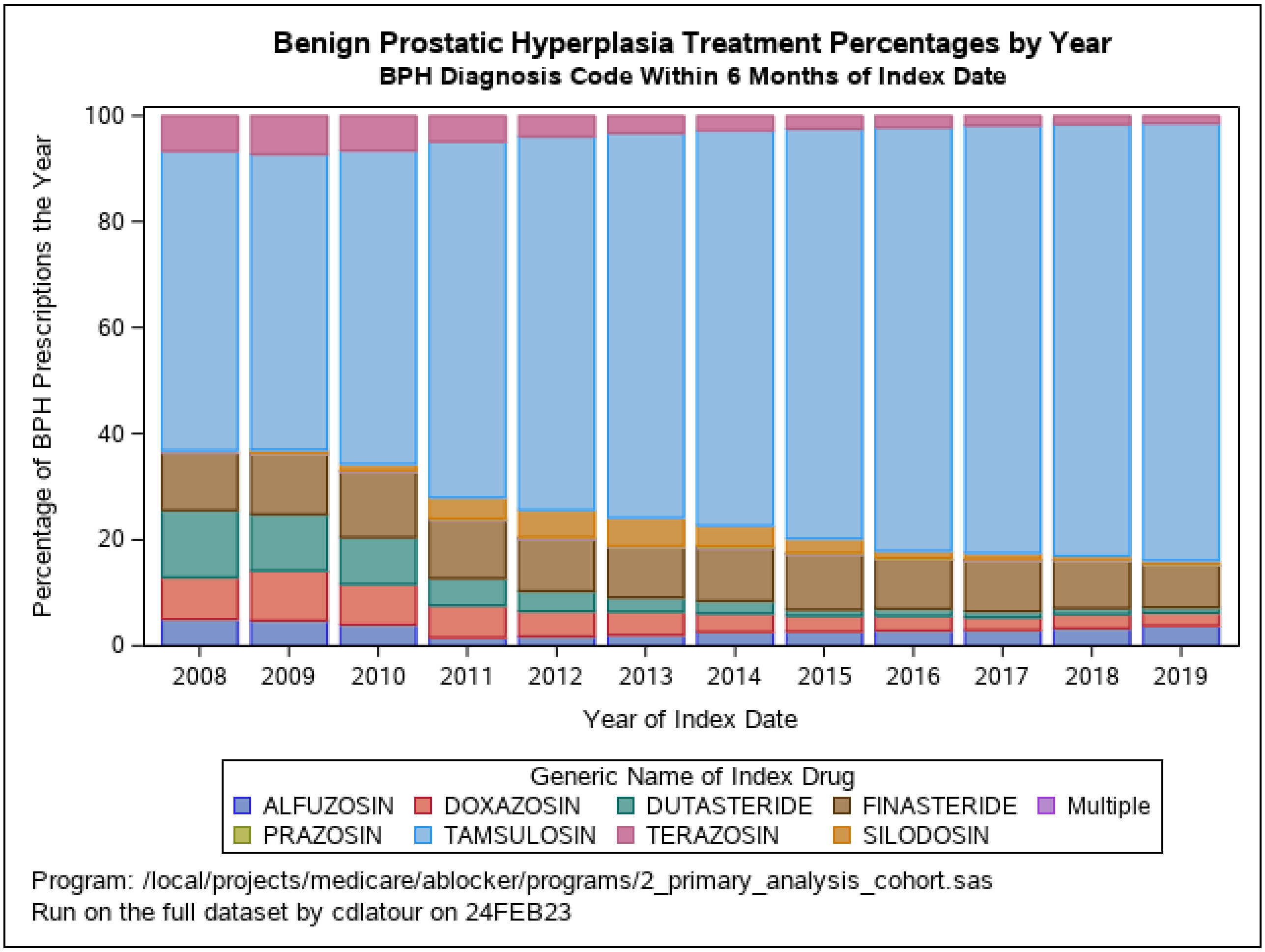

© 2023 Zhang J et al. *JAMA Network Open.*

Table S14. Primary study results after 1-year of follow-up, additionally controlling for indicators of severity of benign prostatic hyperplasia.

	Inverse Probability of Treatment Weighted			
**Study Outcome**	**Risk Among α-Blocker Initiators (95% CI)**	**Risk Among 5α-Reductase Inhibitors (95% CI)**	**Risk Difference Per 1,000 (95% CI)**	**Risk Ratio (95% CI)**
Hospitalization for Heart Failure	3.82 (3.72, 3.92)	3.95 (3.64, 4.25)	-1.26 (-4.44, 1.92)	0.97 (0.89, 1.05)
MACE Outcomes	8.92 (8.78, 9.06)	8.48 (8.06, 8.91)	4.41 (-0.01, 8.82)	1.05 (1.00, 1.11)
Composite MACE or Hospitalization for Heart Failure	11.11 (10.96, 11.27)	10.63 (10.16, 11.09)	4.89 (0.06, 9.72)	1.05 (1.00, 1.09)
Death from Any Cause	6.01 (5.89, 6.12)	5.83 (5.47, 6.19)	1.78 (-1.95, 5.50)	1.03 (0.97, 1.10)

Study results after controlling for days since first recorded BPH diagnosis (0 to <180 days, 180 to <365 days, ≥365 days) and the number of BPH diagnosis codes in the 12 months prior to new use of the study drug (categorical: 1, 2, ≥3).

© 2023 Zhang J et al. *JAMA Network Open.*

Supplemental Table S15. Study results after removing patients with a history of an anticoagulant fill within 1-year prior to their new-use episode (n=163,320).

	Inverse Probability of Treatment Weighted			
Study Outcome	Risk Among α-Blocker Initiators	Risk Among 5α-Reductase Inhibitors	Risk Difference Per 1,000 (95% CI)	Risk Ratio (95% CI)
Hospitalization for Heart Failure	2.86 (2.77, 2.95)	2.82 (2.55, 3.09)	0.41 (-2.49, 3.30)	1.02 (0.92, 1.13)
MACE Outcomes	8.03 (7.89, 8.18)	7.41 (7.01, 7.80)	6.28 (2.01, 10.56)	1.09 (1.02, 1.15)
Composite MACE or Hospitalization for Heart Failure	9.66 (9.50, 9.82)	8.95 (8.52, 9.39)	7.04 (2.31, 11.78)	1.08 (1.02, 1.14)
Death from Any Cause	5.32 (5.21, 5.44)	4.82 (4.51, 5.14)	5.00 (1.56, 8.44)	1.10 (1.03, 1.18)

All estimates are inverse probability of treatment weighted, using the primary confounder set.

© 2023 Zhang J et al. *JAMA Network Open.*

Supplemental Table S16. Study results restricting to new-use periods on or after October 1, 2015 (i.e., use of ICD-10 codes in Medicare data) (n = 77,302).

	Inverse Probability of Treatment Weighted			
**Study Outcome**	**Risk Among α-Blocker Initiators**	**Risk Among 5α-Reductase Inhibitors**	**Risk Difference Per 1,000 (95% CI)**	**Risk Ratio (95% CI)**
Hospitalization for Heart Failure	3.80<br>(3.65, 3.96)	3.94<br>(3.41, 4.47)	-1.38<br>(-6.74, 3.99)	0.97<br>(0.84, 1.11)
MACE Outcomes	8.40<br>(8.18, 8.62)	7.92<br>(7.23, 8.61)	4.77<br>(-2.36, 11.90)	1.06<br>(0.97, 1.16)
Composite MACE or Hospitalization for Heart Failure	10.53<br>(10.29, 10.77)	9.87<br>(9.11, 10.63)	6.58<br>(-1.32, 14.48)	1.07<br>(0.99, 1.16)
Death from Any Cause	5.37<br>(5.19, 5.55)	5.17<br>(4.58, 5.75)	2.02<br>(-4.03, 8.07)	1.04<br>(0.93, 1.17)

All estimates are inverse probability of treatment weighted, using the primary confounder set.

© 2023 Zhang J et al. *JAMA Network Open.*

Table S17. Study estimates for hospitalization for injury or poisoning as a negative control outcome.

	Inverse Probability of Treatment Weighted			
**Study Outcome**	**Risk Among α-Blocker Initiators**	**Risk Among 5α-Reductase Inhibitors**	**Risk Difference Per 1,000 (95% CI)**	**Risk Ratio (95% CI)**
Hospitalization for Pneumonia	5.35<br>(5.23, 5.46)	5.06<br>(4.75, 5.37)	2.87<br>(-0.42, 6.16)	1.06<br>(0.99, 1.13)

© 2023 Zhang J et al. *JAMA Network Open.*

Table S18. Results from the quantitative bias analysis.

Confounder	Differential or Nondifferential Misclassification by MACE Outcome	Sensitivity Among People with MACE Outcomes	New Study Estimate of the Risk Ratio, Adjusted for Confounder Misclassification[a]
**Obesity or Severe Obesity[b]**	Non-Differential	36%	1.07
	Higher sensitivity among those with MACE	41%	1.09
		46%	1.11
**Smoking/Tobacco Use[c]**	Non-Differential	30%	0.94
	Higher sensitivity among those with MACE	35%	0.97
		40%	1.03

[a] This was calculated via standardization.
[b] The sensitivity among people without MACE outcomes was 36%, and the specificity, regardless of MACE outcome, was set as 97%.
[c] The sensitivity among people without MACE outcomes was 30%, and the specificity, regardless of MACE outcome, was set as 100%.

© 2023 Zhang J et al. *JAMA Network Open.*

Table S19. Descriptive statistics of the study population restricted to only those individuals with ≥2 outpatient or ≥1 inpatient diagnosis code (any position) for BPH (n=82,626).

Confounder Variable	Pre-Weighting Cohort After PS Trimming of Non-Overlapping Regions			Cohort After Applying Inverse Probability of Treatment Weights		
	Prevalence Among a-Blocker Initiators (N = 72,450) N (%)	Prevalence Among 5a-Reductase Inhibitor Initiators (N = 14,176) N (%)	Absolute SMD	Prevalence Among a-Blocker Initiators %	Prevalence Among 5a-Reductase Inhibitor Initiators %	Absolute SMD
Age, Median (IQR)	74.0 (70.0-80.0)	75.0 (70.0-80.0)	0.065	74.0 (70.0-80.0)	74.0 (70.0-80.0)	0.012
Calendar Year, Median (IQR)	2014.0 (2011.0-2017.0)	2013.0 (2010.0-2016.0)	0.288	2014.0 (2011.0-2017.0)	2014.0 (2011.0-2017.0)	0.006
Race/Ethnicity						
American Indian/Alaska Native	232 (0.3%)	34 (0.2%)	0.015	0.3%	0.4%	0.009
Asian/Pacific Islander	2,635 (3.6%)	450 (3.2%)	0.026	3.6%	3.7%	0.005
Black or African American	4,470 (6.2%)	766 (5.4%)	0.033	6.1%	6.4%	0.014
Non-Hispanic, White	58,525 (80.8%)	11,660 (82.3%)	0.038	81.0%	80.4%	0.014
Other	852 (1.2%)	148 (1.0%)	0.013	1.2%	1.2%	0.000
Hispanic	4,848 (6.7%)	958 (6.8%)	0.003	6.7%	6.8%	0.004
Unknown	888 (1.2%)	160 (1.1%)	0.009	1.2%	1.2%	0.002
Acute Urinary Retention	23,438 (32.4%)	3,225 (22.7%)	0.216	30.8%	30.6%	0.003
Coronary Heart Disease	33,016 (45.6%)	5,942 (41.9%)	0.074	45.0%	45.3%	0.005
Hospitalization due to Heart Failure	8,124 (11.2%)	1,038 (7.3%)	0.134	10.6%	10.9%	0.011
Chronic Kidney Disease	25,684 (35.5%)	3,862 (27.2%)	0.178	34.1%	34.6%	0.010
COPD	19,805 (27.3%)	3,108 (21.9%)	0.126	26.5%	26.8%	0.007
Hypercholesterolemia	38,105 (52.6%)	8,269 (58.3%)	0.116	53.5%	53.8%	0.005
Hospitalization due to myocardial infarction	2,186 (3.0%)	288 (2.0%)	0.063	2.9%	3.0%	0.006
Hospitalization due to Stroke	3,756 (5.2%)	412 (2.9%)	0.116	4.8%	5.0%	0.010
Percutaneous Coronary Intervention	1,531 (2.1%)	269 (1.9%)	0.015	2.1%	2.2%	0.007
Coronary Artery Bypass Graft Surgery	1,059 (1.5%)	100 (0.7%)	0.073	1.3%	1.4%	0.004
Tobacco Use	15,478 (21.4%)	2,172 (15.3%)	0.157	20.4%	20.3%	0.001
ACE Inhibitor, Any Use	27,093 (37.4%)	4,956 (35.0%)	0.051	37.0%	36.9%	0.002

© 2023 Zhang J et al. *JAMA Network Open.*

ARB, Any Use	15,176 (20.9%)	2,765 (19.5%)	0.036	20.7%	21.1%	0.008
Beta-Blocker, Any Use	34,185 (47.2%)	6,062 (42.8%)	0.089	46.5%	46.5%	0.001
Peripheral Vasodilators, Any Use	1,401 (1.9%)	312 (2.2%)	0.019	2.0%	2.0%	0.004
Calcium Channel Blocker, Any use	22,810 (31.5%)	3,778 (26.7%)	0.107	30.7%	30.5%	0.004
Thiazide Diuretics, Any Use	14,863 (20.5%)	2,814 (19.9%)	0.017	20.4%	20.1%	0.008
Combination Diuretics, Any Use	7,721 (10.7%)	1,577 (11.1%)	0.015	10.7%	10.6%	0.003
Potassium Sparing Diuretic, Any Use	4,445 (6.1%)	762 (5.4%)	0.033	6.0%	6.1%	0.003
Loop Diuretic, Any Use	13,326 (18.4%)	2,083 (14.7%)	0.100	17.8%	18.2%	0.009
Other Diuretics, Any Use	1,691 (2.3%)	233 (1.6%)	0.049	2.2%	2.3%	0.004
Anticoagulant Use						
No Fill	60,982 (84.2%)	11,933 (84.2%)	0.000	84.2%	84.2%	0.001
1 Fill	2,613 (3.6%)	345 (2.4%)	0.069	3.4%	3.4%	0.002
≥2 Fills	8,855 (12.2%)	1,898 (13.4%)	0.035	12.4%	12.4%	0.000
Opioid Use						
No Fill	41,223 (56.9%)	9,168 (64.7%)	0.160	58.2%	58.2%	0.000
1 Fill	12,794 (17.7%)	2,284 (16.1%)	0.041	17.4%	17.3%	0.002
≥2 Fills	18,433 (25.4%)	2,724 (19.2%)	0.150	24.4%	24.5%	0.001
Nicotine or Varenicline, Any Use	327 (0.5%)	49 (0.3%)	0.017	0.4%	0.4%	0.003
Statin, Any Use	42,670 (58.9%)	7,906 (55.8%)	0.063	58.4%	58.3%	0.002
Diabetes	25,206 (34.8%)	4,166 (29.4%)	0.116	33.9%	34.3%	0.008
DPP-4i	3,214 (4.4%)	456 (3.2%)	0.064	4.2%	4.3%	0.001
GLP-1	666 (0.9%)	86 (0.6%)	0.036	0.9%	0.9%	0.005
Long-Acting Insulin						
No Fill	66,815 (92.2%)	13,419 (94.7%)	0.099	92.6%	92.6%	0.001
1 Fill	942 (1.3%)	99 (0.7%)	0.061	1.2%	1.2%	0.004
≥2 Fills	4,693 (6.5%)	658 (4.6%)	0.080	6.2%	6.3%	0.003
Short-Acting Insulin						
No Fill			0.059	96.0%	95.9%	0.002
1 Fill	69,360 (95.7%)	13,766 (97.1%)	0.037	1.3%	1.3%	0.001
≥2 Fills	993 (1.4%)	117 (0.8%)	0.046	2.8%	2.8%	0.003
SGLT-2i, Any Fill	455 (0.6%)	59 (0.4%)	0.029	0.6%	0.6%	0.001
Sulfonylureas, Any Fill	8,054 (11.1%)	1,262 (8.9%)	0.074	10.8%	10.7%	0.001
TZD, Any Fill	2,170 (3.0%)	386 (2.7%)	0.016	3.0%	3.0%	0.002

© 2023 Zhang J et al. *JAMA Network Open.*

Atherosclerosis or Peripheral Vascular Disease	23,405 (32.3%)	4,667 (32.9%)	0.013	32.4%	32.8%	0.009
Obesity	8,743 (12.1%)	1,130 (8.0%)	0.137	11.4%	11.6%	0.005

The sum of the standardized inverse probability of treatment weights among those treated with alpha-blockers was 72,453; the sum among those treated with 5-alpha reductase inhibitors was 14,175.

© 2023 Zhang J et al. *JAMA Network Open.*

Table S20. Study results among those patients with ≥2 outpatient or ≥1 inpatient diagnosis code for benign prostatic hyperplasia (n = 82,626).

	Inverse Probability of Treatment Weighted			
**Study Outcome**	**Risk Among α-Blocker Initiators**	**Risk Among 5α-Reductase Inhibitors**	**Risk Difference Per 1,000 (95% CI)**	**Risk Ratio (95% CI)**
Hospitalization for Heart Failure	4.53 (4.38, 4.68)	4.87 (4.43, 5.31)	-3.43 (-8.07, 1.21)	0.93 (0.85, 1.02)
MACE Outcomes	10.84 (10.62, 11.07)	10.23 (9.65, 10.80)	6.15 (0.13, 12.17)	1.06 (1.00, 1.12)
Composite MACE or Hospitalization for Heart Failure	13.35 (13.11, 13.59)	12.68 (12.05, 13.30)	6.69 (0.23, 13.16)	1.05 (1.00, 1.11)
Death from Any Cause	7.55 (7.37, 7.74)	6.98 (6.47, 7.49)	5.77 (0.40, 11.13)	1.08 (1.00, 1.17)

All estimates are inverse probability of treatment weighted, using the primary confounder set.

© 2023 Zhang J et al. *JAMA Network Open.*

Table S21. Descriptive statistics of the study population restricted to only those individuals with at least in in-patient hospitalization for myocardial infarction, stroke, or heart failure within 1 year prior to cohort entry.

	**Pre-Weighting Cohort After Trimming Individuals in Non-Overlapping Regions of the Propensity Score Distribution**			**Cohort After Applying Stabilized Inverse Probability of Treatment Weights**		
	**Prevalence Among a-Blocker Initiators (N = 17,606)**	**Prevalence Among 5a-Reductase Inhibitor Initiators (N = 2,085)**		**Prevalence Among a-Blocker Initiators**	**Prevalence Among 5a-Reductase Inhibitor Initiators**	
**Confounder Variable**	**N (%)**	**N (%)**	**SMD**	**%**	**%**	**SMD**
Age, Median (IQR)	77.0 (71.0-82.0)	79.0 (73.0-84.0)	0.202	77.0(72.0-83.0)	77.0(72.0-83.0)	0.025
Calendar Year, Median (IQR)	2014.0 (2011.0-2017.0)	2013.0 (2010.0-2016.0)	0.250	2014.0(2011.0-2017.0)	2014.0(2011.0-2017.0)	0.022
Race/Ethnicity[a]						
Unknown	NTSR	NTSR	0.053	0.7%	0.6%	0.008
Non-Hispanic, White	14,397 (81.8%)	1,734 (83.2%)	0.037	81.9%	81.2%	0.018
Black or African American	1,421 (8.1%)	180 (8.6%)	0.020	8.1%	8.2%	0.002
Other	NTSR	NTSR	0.070	NTSR	NTSR	0.000
Asian/Pacific Islander	382 (2.2%)	45 (2.2%)	0.001	2.2%	2.1%	0.005
Hispanic	1,051 (6.0%)	102 (4.9%)	0.048	5.9%	6.6%	0.031
American Indian/Alaska Native	NTSR	NTSR	0.008	NTSR	NTSR	0.001
Acute Urinary Retention	8,011 (45.5%)	785 (37.6%)	0.160	44.7%	44.7%	0.000
Coronary Heart Disease	14,285 (81.1%)	1,726 (82.8%)	0.043	81.3%	81.4%	0.003
Chronic Kidney Disease	11,439 (65.0%)	1,263 (60.6%)	0.091	64.5%	64.4%	0.001
COPD	9,071 (51.5%)	1,057 (50.7%)	0.017	51.4%	50.8%	0.012
Hypercholesterolemia	9,770 (55.5%)	1,276 (61.2%)	0.116	56.1%	56.5%	0.007
Percutaneous Coronary Intervention	1,535 (8.7%)	231 (11.1%)	0.079	9.0%	9.3%	0.010
Coronary Artery Bypass Graft Surgery	963 (5.5%)	85 (4.1%)	0.065	5.3%	5.5%	0.006
Tobacco Use	6,630 (37.7%)	683 (32.8%)	0.103	37.1%	36.2%	0.020
ACE Inhibitor, Any Use	8,620 (49.0%)	1,011 (48.5%)	0.009	48.9%	48.9%	0.001
Angiotensin II receptor blocker, Any Use	4,010 (22.8%)	427 (20.5%)	0.056	22.5%	23.3%	0.018
Beta-Blocker, Any Use	13,344 (75.8%)	1,541 (73.9%)	0.043	75.6%	76.5%	0.021
Peripheral Vasodilators, Any Use	368 (2.1%)	52 (2.5%)	0.027	2.1%	2.0%	0.010
Calcium Channel Blocker, Any use	6,961 (39.5%)	685 (32.9%)	0.139	38.8%	39.4%	0.011
Thiazide Diuretics, Any Use	2,987 (17.0%)	302 (14.5%)	0.068	16.7%	16.8%	0.002
Combination Diuretics, Any Use	1,371 (7.8%)	147 (7.1%)	0.028	7.7%	7.9%	0.006
Potassium Sparing Diuretic, Any Use	2,545 (14.5%)	279 (13.4%)	0.031	14.3%	14.5%	0.005
Loop Diuretic, Any Use	9,525 (54.1%)	1,131 (54.2%)	0.003	54.1%	54.5%	0.009

© 2023 Zhang J et al. *JAMA Network Open.*

Other Diuretics, Any Use	1,145 (6.5%)	121 (5.8%)	0.029	6.4%	6.7%	0.012
Anticoagulant Use						
No Fill	12,810 (65.1%)	11,512 (65.4%)	1,298 (62.3%)	65.0%	64.3%	0.015
1 Fill	1,624 (8.2%)	1,471 (8.4%)	153 (7.3%)	8.2%	8.1%	0.007
≥2 Fills	5,257 (26.7%)	4,623 (26.3%)	634 (30.4%)	26.7%	27.6%	0.021
Opioid Use						
No Fill	8,141 (46.2%)	1,091 (52.3%)	0.122	46.9%	47.0%	0.002
1 Fill	3,157 (17.9%)	370 (17.7%)	0.005	17.9%	18.0%	0.003
≥2 Fills	6,308 (35.8%)	624 (29.9%)	0.126	35.2%	35.0%	0.005
Nicotine or Varenicline, Any Use	98 (0.6%)	13 (0.6%)	0.009	0.6%	0.6%	0.000
Statin, Any Use	12,687 (72.1%)	1,441 (69.1%)	0.065	71.7%	71.6%	0.002
Diabetes	8,877 (50.4%)	943 (45.2%)	0.104	49.9%	50.1%	0.004
DPP-4i	1,092 (6.2%)	109 (5.2%)	0.042	6.1%	5.9%	0.008
GLP-1	204 (1.2%)	18 (0.9%)	0.030	1.1%	1.2%	0.004
Long-Acting Insulin						
No Fill	14,591 (82.9%)	1,803 (86.5%)	0.100	83.3%	82.9%	0.010
1 Fill	590 (3.4%)	66 (3.2%)	0.010	3.3%	3.1%	0.014
≥2 Fills	2,425 (13.8%)	216 (10.4%)	0.105	13.4%	14.0%	0.018
Short-Acting Insulin						
No Fill	15,681 (89.1%)	1,902 (91.2%)	0.072	89.3%	89.0%	0.010
1 Fill	683 (3.9%)	61 (2.9%)	0.053	3.8%	4.1%	0.015
≥2 Fills	1,242 (7.1%)	122 (5.9%)	0.049	6.9%	7.0%	0.002
SGLT-2i, Any Fill	NTSR	NTSR	0.016	NTSR	NTSR	0.001
Sulfonylureas, Any Fill	2,926 (16.6%)	309 (14.8%)	0.049	16.4%	16.7%	0.006
TZD, Any Fill	547 (3.1%)	67 (3.2%)	0.006	3.1%	3.3%	0.012
Atherosclerosis or Peripheral Vascular Disease	9,922 (56.4%)	1,317 (63.2%)	0.139	57.1%	57.5%	0.008
Obesity	3,552 (20.2%)	359 (17.2%)	0.076	19.9%	19.4%	0.011

Abbreviations: NTSR = Number too small to report; SMD = Standardized mean difference.
The sum of the standardized inverse probability of treatment weights among individuals in the alpha-blocker treatment group was 17,608; the sum among individuals in the 5-alpha reductase inhibitor treatment group was 2,076. If one treatment group had a number that was too small to report, the other group was marked as having a number too small to report in order to retain category-level SMD values.

© 2023 Zhang J et al. *JAMA Network Open.*

Table S22. Study results among those patients with a history of hospitalization for heart failure, myocardial infarction, or stroke within 1 year prior to new-use of the study medications (n = 19,691).

	Non-IPTW Estimates				IPTW Estimates			
**Study Outcome**	**Risk Among α-Blocker Initiators % (95% CI)**	**Risk Among 5α-Reductase Inhibitor Initiators % (95% CI)**	**Risk Difference Per 1,000 (95% CI)**	**Risk Ratio (95% CI)**	**Risk Among α-Blocker Initiators**	**Risk Among 5α-Reductase Inhibitors**	**Risk Difference Per 1,000 (95% CI)**	**Risk Ratio (95% CI)**
Hospitalization for HF	17.81 (17.23, 18.39)	18.20 (16.5, 19.91)	-3.94 (-22.02, 14.13)	0.98 (0.89, 1.08)	17.83 (17.25, 18.41)	18.79 (16.96, 20.61)	-9.57 (-28.53, 9.38)	0.95 (0.86, 1.05)
MACE Outcomes	26.94 (26.30, 27.58)	27.31 (25.37, 29.25)	-3.73 (-24.57, 17.11)	0.99 (0.91, 1.07)	36.52 (35.79, 37.25)	36.41 (34.13, 38.7)	1.06 (-22.9, 25.02)	1.00 (0.94, 1.07)
Composite MACE or Hospitalization for HF	36.51 (35.78, 37.24)	35.91 (33.71, 38.09)	6.02 (-17.46, 29.50)	1.02 (0.95, 1.09)	36.52 (35.79, 37.25)	36.41 (34.13, 38.7)	1.06 (-22.9, 25.02	1.00 (0.94, 1.07)
Death from Any Cause	19.80 (19.22, 20.38)	20.65 (18.85, 22.45)	-8.43 (-27.57, 10.7)	0.96 (0.87, 1.05)	19.86 (19.29, 20.43)	21.05 (19.06, 23.04)	-11.91 (-32.56, 8.75)	0.94 (0.86, 1.04)

Abbreviations: HF = Heart failure; MACE = Major adverse cardiovascular events

© 2023 Zhang J et al. *JAMA Network Open.*

Table S23. Study results among the primary study population, additionally adjusting for dual eligibility for Medicaid and receipt of the Medicare Part D low-income subsidy (n = 82,626).

	Inverse Probability of Treatment Weighted			
**Study Outcome**	**Risk Among α-Blocker Initiators**	**Risk Among 5α-Reductase Inhibitors**	**Risk Difference Per 1,000 (95% CI)**	**Risk Ratio (95% CI)**
Hospitalization for Heart Failure	3.83 (3.73, 3.93)	3.85 (3.57, 4.14)	-0.23 (-3.21, 2.75)	0.99 (0.92, 1.07)
MACE Outcomes	8.94 (8.80, 9.08)	8.41 (8.00, 8.82)	5.24 (1.05, 9.43)	1.06 (1.01, 1.12)
Composite MACE or Hospitalization for Heart Failure	11.13 (10.98, 11.28)	10.48 (10.04, 10.92)	6.48 (1.91, 11.05)	1.06 (1.02, 1.11)
Death from Any Cause	6.02 (5.90, 6.13)	5.75 (5.41, 6.10)	2.65 (-0.89, 6.20)	1.05 (0.98, 1.11)

All estimates are inverse probability of treatment weighted, using the primary confounder set.

© 2023 Zhang J et al. *JAMA Network Open.*

Discussion S1. Discussion on the limitations of hospitalization for injury or poisoning as a negative control outcome analysis.

As a *post hoc* sensitivity analysis, we considered hospitalization for injury or poisoning as a negative control outcome. Notably, for this outcome to be informative regarding the role of residual confounding, the following conditions would have to be met:[12]

1. There is no effect of ABs nor 5ARIs on the risk of the outcome.
2. The measured confounders from the primary analysis have the same magnitude and direction of effects on the risk of hospitalization for injury or poisoning as they do for the primary study outcomes.

However, it is challenging to be sure that hospitalization for injury or poisoning meets these requirements.

It is likely that the degree to which our negative control outcome deviates from these requirements determines how informative it is for our analyses. In this study, the effect estimates we estimated were small. In that setting, it is possible that even small deviations from these assumptions would impact the interpretation of this estimate. The RR and RD estimates from our negative control outcome are attenuated from the primary estimates, but they are not equivalent to the null estimates for either the RR or RD. Given this context and the inability to test these theoretical assumptions, we cannot make strong conclusions about the role of residual confounding in the primary analyses based upon these results.

© 2023 Zhang J et al. *JAMA Network Open.*

References

1. Birman-Deych E, Waterman AD, Yan Y, Nilasena DS, Radford MJ, Gage BF. Accuracy of ICD-9-CM Codes for Identifying Cardiovascular and Stroke Risk Factors. *Med Care*. 2005;43(5):480-485.
2. Kini V, Mosley B, Raghavan S, et al. Differences in high- and low-value cardiovascular testing by health insurance provider. *J Am Heart Assoc*. 2021;10(3):1-10. doi:10.1161/JAHA.120.018877
3. Suissa K, Schneeweiss S, Lin KJ, Brill G, Kim SC, Patorno E. Validation of obesity-related diagnosis codes in claims data. *Diabetes Obes Metab*. 2021;23(12):2623-2631. doi:10.1111/dom.14512
4. Desai RJ, Solomon DH, Shadick N, Iannaccone C, Kim SC. Identification of smoking using Medicare data - a validation study of claims-based algorithms. *Pharmacoepidemiol Drug Saf*. 2016;25(4):472-475. doi:10.1002/pds.3953
5. Howe CJ, Bailey ZD, Raifman JR, Jackson JW. Recommendations for Using Causal Diagrams to Study Racial Health Disparities. *Am J Epidemiol*. Published online August 2, 2022. doi:10.1093/aje/kwac140
6. Tennant PWG, Murray EJ, Arnold KF, et al. Use of directed acyclic graphs (DAGs) to identify confounders in applied health research: review and recommendations. *Int J Epidemiol*. 2021;50(2):620-632. doi:10.1093/ije/dyaa213
7. Faurot KR, Jonsson Funk M, Pate V, et al. Using claims data to predict dependency in activities of daily living as a proxy for frailty. *Pharmacoepidemiol Drug Saf*. 2015;24(1):59-66. doi:10.1002/pds.3719
8. Faurot K, Duchesneau E, Shmuel S, et al. Translation of a Medicare claims-based frailty algorithm from ICD-9-CM to ICD-10-CM. *Innov Aging*. 2022;6(S1):330.
9. Tielemans SMAJ, de Melker HE, Hahné SJM, et al. Non-specific effects of measles, mumps, and rubella (MMR) vaccination in high income setting: population based cohort study in the Netherlands. *BMJ*. Published online August 30, 2017:j3862. doi:10.1136/bmj.j3862
10. Centers for Medicare & Medicaid Services. *General Equivalence Mappings: ICD-9-CM to and from ICD-10_CM and ICD-10-PCS.*; 2009.
11. Lash T, Fox MP, Fink AK. *Applying Quantitative Bias Analysis to Epidemiologic Data*. Springer Science + Business Media, LLC; 2009.
12. Lipsitch M, Tchetgen Tchetgen E, Cohen T. Negative Controls: A Tool for Detecting Confounding and Bias in Observational Studies. *Epidemiology*. 2010;21(3):383-388. doi:10.1097/EDE.0b013e3181d61eeb

© 2023 Zhang J et al. *JAMA Network Open.*
