# Supplementary material for: Cardiovascular Outcomes of α-Blockers vs 5-α Reductase Inhibitors for Benign Prostatic Hyperplasia
Source: JAMA Netw Open. 2023 Nov 14;6(11):e2343299. doi: 10.1001/jamanetworkopen.2023.43299 (PMC10646730; doi:10.1001/jamanetworkopen.2023.43299)
Supplement: Supplement 3. — Data Sharing Statement [file jamanetwopen-e2343299-s003.pdf]

Data Sharing Statement

Zhang. Cardiovascular Outcomes of α-Blockers vs 5-α Reductase Inhibitors for Benign Prostatic Hyperplasia. *JAMA Netw Open*. Published online November 15, 2023. doi:10.1001/jamanetworkopen.2023.43299

Data
Data available: Yes
Data types: Deidentified participant data
How to access data: These data are not publicly available but can be obtained from the Centers for Medicare and Medicaid Services through an appropriate data use agreement and sufficient funds to cover the cost of the data access.
When available: With publication

Supporting Documents
Document types: Statistical/analytic code
How to access documents: https://github.com/chasedlatour/AB-v-5ARI-Medicare
When available: With publication

Additional Information
Who can access the data: Anyone requesting the data.
Types of analyses: any purpose
Mechanisms of data availability: These data are not publicly available but can be obtained from the Centers for Medicare and Medicaid Services through an appropriate data use agreement and sufficient funds to cover the cost of the data access.
Any additional restrictions: no
